# Structural Simplification of Bedaquiline: the Discovery of 3‐(4‐(*N*,*N*‐Dimethylaminomethyl)phenyl)quinoline‐Derived Antitubercular Lead Compounds

**DOI:** 10.1002/cmdc.201600441

**Published:** 2016-10-28

**Authors:** Chunxian He, Laura Preiss, Bin Wang, Lei Fu, Hui Wen, Xiang Zhang, Huaqing Cui, Thomas Meier, Dali Yin

**Affiliations:** ^1^ State Key Laboratory of Bioactive Substances and Function ofNatural MedicineInstitute of Materia MedicaPeking Union Medical College andChinese Academy of Medical SciencesBeijing100050China; ^2^ Beijing Key Laboratory of Active Substances Discovery and DrugabilityEvaluationInstitute of Materia MedicaPeking Union Medical College andChinese Academy of Medical SciencesBeijing100050China; ^3^ Department of Structural BiologyMax Planck Institute of BiophysicsMax-von-Laue-Str. 360438Frankfurt am MainGermany; ^4^ Department of Pharmacology, Beijing Tuberculosis and Thoracic TumorResearch InstituteBeijing Chest Hospital, Capital Medical University97 Ma Chang StreetBeijing101149China; ^5^ Department of Life SciencesImperial College LondonExhibition RoadLondonSW7 2AZUK

**Keywords:** ATP synthase, bedaquiline, multidrug resistance, *Mycobacterium tuberculosis*, pulmonary tuberculosis

## Abstract

Bedaquiline (BDQ) is a novel and highly potent last‐line antituberculosis drug that was approved by the US FDA in 2013. Owing to its stereo‐structural complexity, chemical synthesis and compound optimization are rather difficult and expensive. This study describes the structural simplification of bedaquiline while preserving antitubercular activity. The compound's structure was split into fragments and reassembled in various combinations while replacing the two chiral carbon atoms with an achiral linkage instead. Four series of analogues were designed; these candidates retained their potent antitubercular activity at sub‐microgram per mL concentrations against both sensitive and multidrug‐resistant (MDR) *Mycobacterium tuberculosis* strains. Six out of the top nine MIC‐ranked candidates were found to inhibit mycobacterial ATP synthesis activity with IC_50_ values between 20 and 40 μm, one had IC_50_>66 μm, and two showed no inhibition, despite their antitubercular activity. These results provide a basis for the development of chemically less complex, lower‐cost bedaquiline derivatives and describe the identification of two derivatives with antitubercular activity against non‐ATP synthase related targets.

## Introduction

Tuberculosis (TB) is a serious threat to human health,^1^, ^2^, ^3^ causing 1.5 million deaths in 2013.^4^, ^5^ The emergence and transmission of multidrug‐resistant (MDR) and extensively drug‐resistant (XDR) TB strains is a particular challenge for global TB prevention and treatment.^3^ Bedaquiline (**1**) is the first novel US Food and Drug Administration (FDA)‐approved anti‐TB drug for the treatment of MDR‐TB cases in the past 40 years.^6^, ^7^, ^8^, ^9^, ^10^, ^11^ In vitro studies demonstrated potent inhibition of mycobacterial growth by bedaquiline, against both drug‐sensitive and drug‐resistant mycobacteria, with a minimum inhibitory concentration (MIC) of 0.06 μg mL^−1^.^9^ Bedaquiline also exhibits excellent clinical efficacy for the treatment of TB patients, particularly those with MDR‐TB infections.^9^ It has a remarkably long half‐life, which was attributed to a high log*P* value (7.52) and cationic, amphiphilic properties resulting in tissue accumulation.^8^, ^12^ Adverse side effects of bedaquiline, such as phospholipidosis and cardiovascular risks, may relate to these molecular features. In particular, an *N*‐desmethyl metabolite (“M2”) was reported to be more toxic but less bactericidal.^8^, ^13^ At the molecular level the compound was shown to inhibit the mycobacterial F_1_F_o_ ATP synthase by binding to its membrane‐embedded F_o_ rotor ring, a ring‐shaped assembly of identical c‐subunit copies.^6^, ^14^ Here, the half‐maximal inhibitory concentration (IC_50_) of bedaquiline, efficiently inhibiting the mycobacterial ATP synthase, was reported to be remarkably low (25 nm). Furthermore, the mode of action of bedaquiline is highly target specific,^14^ as the IC_50_ values for human, bovine, and mouse mitochondrial ATP synthases were found to be 20 000‐fold higher.^10^, ^14^, ^15^

Besides these obvious assets of bedaquiline, its chemical complexity in harboring two adjacent chiral centers, makes the chemical synthesis of this new anti‐TB drug laborious and costly in the production process.^11^, ^16^ Many ongoing research programs are focused on the optimization of bedaquiline to decrease its structural complexity while maintaining its antitubercular activity.^17^, ^18^, ^19^, ^20^, ^21^, ^22^, ^23^, ^24^ Still, new bedaquiline analogues are required to deliver potential new leads.

The aim of this study was therefore to simplify the chemical synthesis of bedaquiline to work toward new, alternative leads. Once these simplified scaffolds are achieved, they will be used to improve pharmacokinetic properties to decrease the adverse side effects of bedaquiline. Based on the previously reported relationship of structure and antitubercular activity of various diarylquinoline analogues,^9^ we dissected bedaquiline into various fragments and reorganized them to obtain novel antitubercular agents with simplified scaffolds. The MIC values of the new compounds were determined, and the best nine candidates were tested for their inhibitory activities against ATP synthesis inhibition in mycobacteria.

## Results and Discussion

### Design strategy

**Figure 1:**
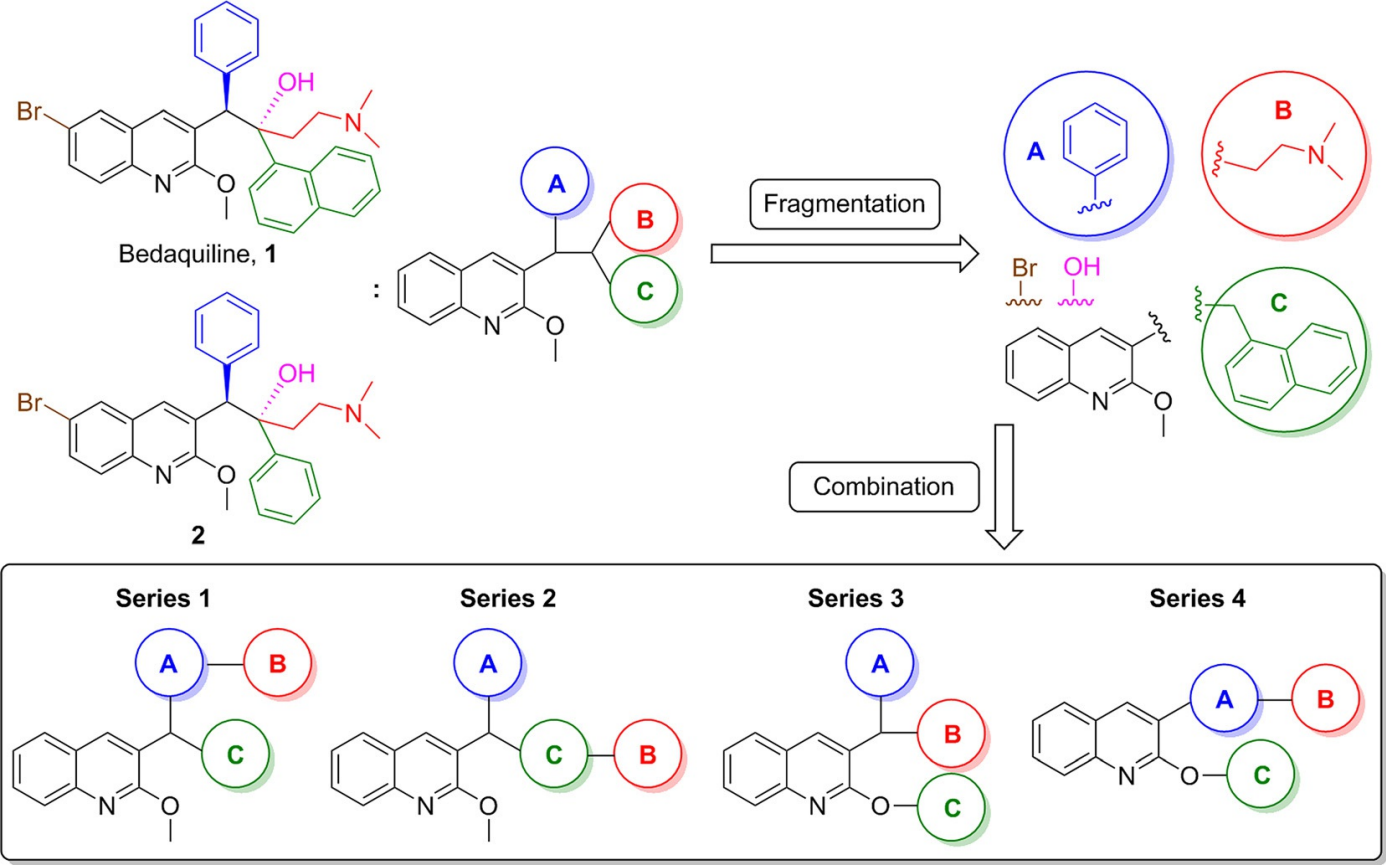
Strategy of bedaquiline simplification. Bedaquiline was split into the core quinoline moiety, three major and two minor fragments (bromo and hydroxy groups). The major fragments comprise the phenyl ring (fragment A), the side chain dimethylaminoethyl (fragment B), and the naphthylmethyl ring (fragment C). These major fragments were reattached to the core quinoline moiety to design four series of new analogues while gradually replacing the chiral linkage.

Bedaquiline (**1**) contains two adjacent chiral carbon atoms that bridge three aryl rings and a dimethylaminoethyl moiety (Figure 1). The structure and antitubercular activity relationship of diarylquinoline analogues as well as the importance and contribution of each fragment of bedaquiline to its antitubercular activity have been quantitatively evaluated.^9^ It was shown that the activity of bedaquiline is closely related to the spatial distribution of its segments. Some fragments, such as the quinolino and dimethylamino groups, are necessary to maintain its activity, whereas other groups such as the bromo and hydroxy groups are less important. Therefore, we split bedaquiline into the core quinoline moiety, three major fragments (phenyl ring, naphthylmethyl, and dimethylaminoethyl), and two minor fragments (bromo and hydroxy) (Figure 1). We then reassembled the quinoline core with three major fragments to design four new series of scaffolds. Notably, previous structure–activity relationship (SAR) studies of bedaquiline and its analogue **2** suggested that the naphthylmethyl group can be replaced with a substituted benzyl ring.^9^ Thus, in our chemical optimization, we also used the substituted benzyl ring instead of a naphthylmethyl group to design some new compounds. In addition, the minor bromo and hydroxy groups were added to the final compounds, as desired.

In series 1, fragments A and C were attached to the tertiary carbon atom at the 3‐position of the quinoline moiety, and fragment B was attached to fragment A. In series 2, fragments A and C were attached to the tertiary carbon at the 3‐position, while fragment B was attached to fragment C. In series 3, fragments A and B were attached to the tertiary carbon at the 3‐position, while fragment C was moved down to the 2‐oxyl position. Finally, in series 4 fragment A was attached to the 3‐position of the quinoline moiety, fragment B was attached to fragment A, and fragment C was moved down to the 2‐oxyl position. From series 1 to 4, we systematically simplified the structure of bedaquiline to remove the two chiral carbons and to understand the structure antimycobacterial activity relationship of these new analogues.

### First round of optimization

#### Compounds of series 1

**Scheme 1:**
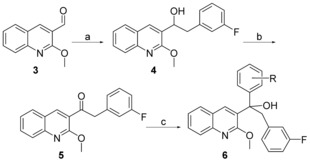
Synthesis of compound **6**. *Reagents and conditions*: a) (3‐fluorobenzyl)magnesium bromide, THF, RT, 2 h, 66.5 %; b) Dess–Martin periodinane, CH_2_Cl_2_, RT, 3 h, 94.8 %; c) substituted phenylmagnesium bromide, THF, RT, 2 h, 56.1–86.9 %.

**Scheme 2:**
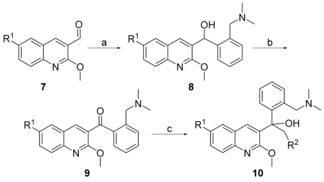
Synthesis of compound **10**. *Reagents and conditions*: a) (2‐((dimethylamino)methyl)phenyl)magnesium bromide, THF, RT, 2 h, 72.1–83.8 %; b) TBAP/NMO, 1,4‐dioxane, RT, 15 h, 77.2–87.5 %; c) Grignard reagent, THF, RT, 2 h, 38.1–56.2 %.

The synthesis of compound **6** (Scheme 1) began with a nucleophilic addition of (3‐fluorobenzyl)magnesium bromide to 2‐methoxyquinoline‐3‐carbaldehyde to give **4**. Intermediate **4** was oxidized with Dess–Martin periodinane to produce **5**,^25^ which was reacted with an appropriate Grignard reagent to give **6**. Compound **10** (Scheme 2) was obtained by reacting 2‐methoxyquinoline‐3‐carbaldehyde with (2‐((dimethylamino)methyl)phenyl)magnesium bromide to produce intermediate **8**. Oxidizing **8** with TBAP/NMO in 1,4‐dioxane afforded **9**,^26^ which was then treated with a Grignard reagent to give **10**.

In series 1 (Table 1), we designed compounds, in which fragment B was attached to fragment A. In total, eight compounds were synthesized, which provided primary information about the SAR of the compounds in series 1. An antitubercular activity screening revealed that compound **6 a** has the most potent antitubercular activity (1 μg mL^−1^) in this series of compounds, while the growth of mammalian cells was inhibited with CC_50_>64 μg mL^−1^. An identical molecule, **10 a**, harboring a bromide, is less active, suggesting that it is not required to preserve antitubercular activity. In addition, relative to compound **10 a**, compound **10 b** has an additional methylene unit extension between fragment C and the other segments. However, compound **10 b** shows less potency against the growth of mycobacterium. Thus, the greater distance between fragment C and the main scaffold likely disfavors its antitubercular activity. Other analogues from this series with similar structures exhibited only weak activity against mycobacteria, indicating that the arrangement of the fragments in series 1 is not suitable to achieve the desired antimycobacterial activity. Note that all candidates of this series still contain one chiral carbon, and racemic mixtures were used to generate CC_50_ and MIC values.

**Table 1:** In vitro antitubercular activity of series 1 compounds.^[a]^

	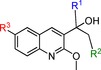							
Compd	R^1^	R^2^	R^3^		MIC		CC_50_^[b]^	
					μg mL^−1^	μm	μg mL^−1^	μm
**6 a**	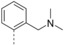	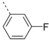	H		1.0	2.3	>64	>148.8
**6 b**	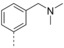	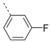	H		6.8	15.8	7.6	17.6
**6 c**	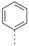	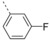	H		>32	>85.8	26	69.7
**6 d**	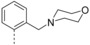	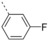	H		>32	>67.8	>64	>135.6
**10 a**	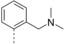	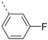	Br		16	31.5	>64	>126.0
**10 b**	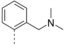	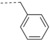	Br		>32	>63.4	59	117.0
**10 c**	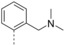	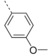	Br		>32	>61.4	1.4	2.7
**10 d**	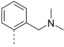	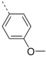	OCH_3_		>32	>67.8	>64	>135.6

[a] Reference compound: bedaquiline MIC=0.060 μg mL^−1^ (0.1 μm). [b] Concentration at which the growth of Vero cells is inhibited by 50 %.

#### Compounds of series 2

**Scheme 3:**
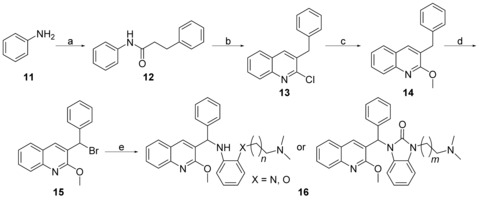
Synthesis of N‐linked compounds **16**. *Reagents and conditions*: a) hydrocinnamoyl chloride, Et_3_N, CH_2_Cl_2_, RT, 5 h, 93.6–98.2 %; b) POCl_3_, DMF, 100 °C, 16 h, 46.0–82.2 %; c) NaOMe/MeOH, reflux, 6 h, 98.2–99.3 %; d) NBS/CCl_4_, reflux, 3 h; e) 1. substituted aniline, K_2_CO_3_, DMF, RT, 2 h, or 2. multistep (see Supporting Information for details).

Aniline (**11**) reacted with hydrocinnamoyl chloride to afford **12**, which was then treated with phosphoryl chloride and DMF at 100 °C to provide **13** (Scheme 3). The reaction of **13** with sodium methoxide produced **14** via a S_N_2 reaction. Next, **14** was activated by *N*‐bromosuccinimide (NBS) to make **15**. The intermediate **15** was treated with substituted aniline and potassium carbonate at room temperature, or followed by a reaction with various aminoalkyl bromides to produce **16**. The synthesis of the derivatives required minor modifications (Supporting Information).

In series 2 (Table 2), fragment B was attached to fragment C. The nitrogen or oxygen atoms located close to fragment C were placed to mimic the hydroxy group of bedaquiline. A total of six compounds were synthesized in this series. The activity screening revealed that most of the compounds in series 2 exhibit moderate antitubercular activity. Compound **16 e**, however, was nearly inactive, which might have been caused by the very short distance between the terminal amine and fragment C of the molecule. In addition, we can also conclude from series 2 that the bromide is not important to retain the antitubercular activity of this compound type. Furthermore, there is one chiral carbon left in the structures of all candidates in this series, and the racemic mixtures were used to generate CC_50_ and MIC values. As none of the compounds from series 2 exhibited sufficient antimycobacterial activity in the very low‐micromolar range, these compounds were not chosen for further chemical optimization.

**Table 2:** In vitro antitubercular activity of series 2 compounds.^[a]^

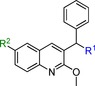							
Compd	R^1^	R^2^		MIC		CC_50_^[b]^	
				μg mL^−1^	μm	μg mL^−1^	μm
**16 a**	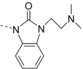	H		7.7	17.0	14	30.9
**16 b**	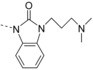	H		6.4	13.7	12	25.7
**16 c**	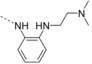	H		3.8	8.9	8.9	20.8
**16 d**	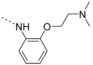	Br		4.2	8.3	30	59.3
**16 e**	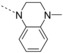	Br		>32	>67.5	>64	>135.0
**16 f**	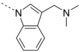	Br		2.0	4.0	9.2	18.4

[a] Reference compound: bedaquiline MIC=0.060 μg mL^−1^ (0.1 μm). [b] Concentration at which the growth of Vero cells is inhibited by 50 %.

#### Compounds of series 3

**Scheme 4:**
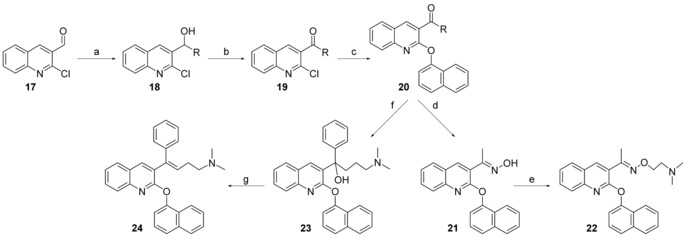
Synthesis of series 3 compounds. *Reagents and conditions*: (a) methylmagnesium iodide or phenylmagnesium bromide, THF, 0 °C, 76.4–78.5 %; b) Dess–Martin periodinane, CH_2_Cl_2_, RT, 92.7–95.5 %; c) 1‐naphthol, K_2_CO_3_, DMSO, 100 °C, 86.9–92.9 %; d) hydroxylamine hydrochloride, Et_3_N, EtOH, RT, 84.6 %; e) 2‐dimethylaminoethyl chloride hydrochloride, NaH, THF, 66.8 %; f) (3‐(dimethylamino)propyl)magnesium chloride, 95.7 %; g) HCl/EtOH, reflux, 72.7 %.

The preparation of compounds **22**–**24** (Scheme 4) started from 2‐chloroquinoline‐3‐carbaldehyde (**17**) with methylmagnesium iodide or phenylmagnesium bromide to provide secondary alcohol intermediates **18**. The oxidation of two **18** analogues with Dess–Martin periodinane produced **19**, which was then reacted with 1‐naphthol to give **20**. The synthesis of **22** was completed by the condensation of **20** with hydroxylamine hydrochloride, and dimethylaminoethyl chloride successively. The treatment of **20** with (3‐(dimethylamino)propyl)magnesium chloride gave **23**. Compound **24** was obtained by heating **23** under acidic conditions (25 % *w*/*w* hydrochloric acid in alcohol).

In series 3 (Table 3) fragments A and B were joined with a methylene linker forming a planar configuration. In addition fragment C was added to the quinoline core by an ether bond. This structure allowed us to remove the two chiral carbons. According to this strategy, we synthesized compounds **22** and **24**. Compound **23** was synthesized for structure–activity comparison. An antitubercular evaluation showed that all three compounds (**22**–**24**) from this series exert a reasonably potent activity against mycobacteria. This suggests that placing fragment C in the 2‐methoxyl position maintains the antitubercular activity. The comparison of compounds **23** and **24** further reveals that the planar configuration between fragments A and B does not affect the antitubercular activity. It also indicates that the hydroxy group is not important for the antitubercular activity of this new scaffold. Moreover, none of the compounds from series 3 contains the bromide. Hence it does not affect the antitubercular activity of this series of compounds, in agreement with the SAR information from series 1 and 2.

**Table 3:** In vitro antitubercular activity of series 3 compounds.^[a]^

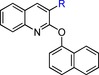						
Compd	R		MIC		CC_50_^[b]^	
			μg mL^−1^	μm	μg mL^−1^	μm
**22**	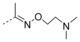		4.0	10.0	11	27.5
**23**	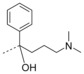		2.0	4.3	4.4	9.5
**24**	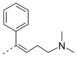		1.8	4.1	10	22.5

[a] Reference compound: bedaquiline MIC=0.060 μg mL^−1^ (0.1 μm). [b] Concentration at which the growth of Vero cells is inhibited by 50 %.

#### Compounds of series 4

**Scheme 5:**
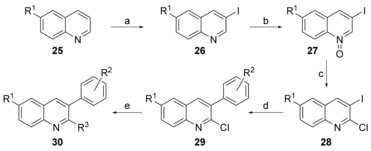
Synthesis of series 4 compounds. *Reagents and conditions*: a) *N*‐iodosuccinimide, AcOH, 100 °C, 28 h, 47.4–46.9 %; b) *m*CPBA, CHCl_3_, 3 h, RT, 90.6–92.7 %; c) POCl_3_, CHCl_3_, 3 h, reflux, 72.9–85.7 %; d) 4‐(dimethylamino)methylphenylboronic acid, Pd(PPh_3_)_4_, Na_2_CO_3_, toluene/H_2_O, 14 h, 76.3–91.5 %; e) benzyl alcohol/phenol/thiophenol, NaH, THF, RT, 6 h, 68.0–99.3 %.

The design of series 4 compounds was based on the finding that the planar configuration of compound **24** (series 3) showed no negative effect on the antitubercular activity. Thus, we moved the phenyl ring (fragment A) to the double bond position between the quinoline and basic amino groups, but kept fragment C attached to the 2‐position of the quinoline. The synthesis of 3‐aromatic analogues **30** is shown in Scheme 5. The synthesis of key substituted quinoline intermediates **26** started from substituted quinoline **25**. The reaction of **25** and *N*‐iodosuccinimide gave 3‐iodoquinoline **26**, which was then treated with *meta*‐chloroperoxybenzoic acid (*m*CPBA) to produce **27**, and chlorinated with phosphoryl chloride to give 2‐chloro‐3‐iodoquinoline **28**. Target compounds **30** were prepared via Suzuki–Miyaura coupling of compound **28** with the corresponding substituted boric acid or boronic acid pinacol cyclic ester to obtain **29**.^27^ Subsequently, the chloro moiety was exchanged with benzyl alcohol, aromatic alcohol, phenol, thiophenol, or aromatic amine by a nucleophilic substitution to obtain **30**.

In total, 14 compounds were synthesized for series 4 (**30 a**–**30 n**; Table 4), all of which contain no more chiral centers. Intriguingly, series 4 compounds were generally potent against mycobacteria with MIC values of ∼1 μg mL^−1^, whereas their CC_50_ values were 10‐fold higher on average. Comparison of the structures and antitubercular activities of series 4 compounds shows that the length of the terminal amine chain can be 1–2 carbons (compounds **30 a**, **30 b**) while retaining antitubercular activity. Further, the presence of the terminal basic nitrogen atom was essential to sustain the antitubercular activity (compounds **30 h**, **30 m**), whereas the bromo group was not (compounds **30 e**, **30 f**).

**Table 4:** In vitro antitubercular activity of series 4 compounds.^[a]^

	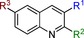							
Compd	R^1^	R^2^	R^3^		MIC		CC_50_^[b]^	
					μg mL^−1^	μm	μg mL^−1^	μm
**30 a**	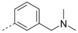	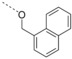	Br		1.0	2.0	10	20.1
**30 b**	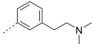	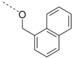	Br		2.0	3.9	3.8	7.4
**30 c**	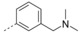	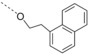	Br		1.8	3.5	12	23.4
**30 d**	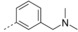	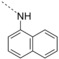	Br		1.6	3.3	25	51.9
**30 e**	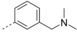	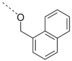	OMe		1.8	4.0	12	26.7
**30 f**	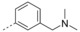	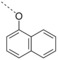	H		2.0	4.9	15	37.1
**30 g**	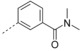	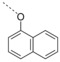	H		>32	76.5	>64	>153.1
**30 h**	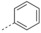	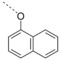	Br		>32	>75.1	>64	>150.2
**30 i**	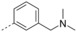	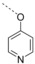	Br		>32	>73.7	>64	>147.5
**30 j**	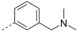	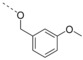	Br		1.6	3.3	4.4	9.2
**30 k**	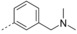	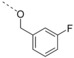	Br		1.9	4.0	11	23.6
**30 l**	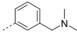	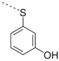	Br		1.0	2.2	11	23.6
**30 m**	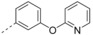	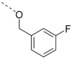	Br		>32	>63.8	>64	>127.8
**30 n**	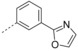	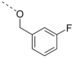	Br		>32	>67.5	3.9	8.2

[a] Reference compound: bedaquiline MIC=0.060 μg mL^−1^ (0.1 μm). [b] Concentration at which the growth of Vero cells is inhibited by 50 %.

### Second round of optimization

**Figure 2:**
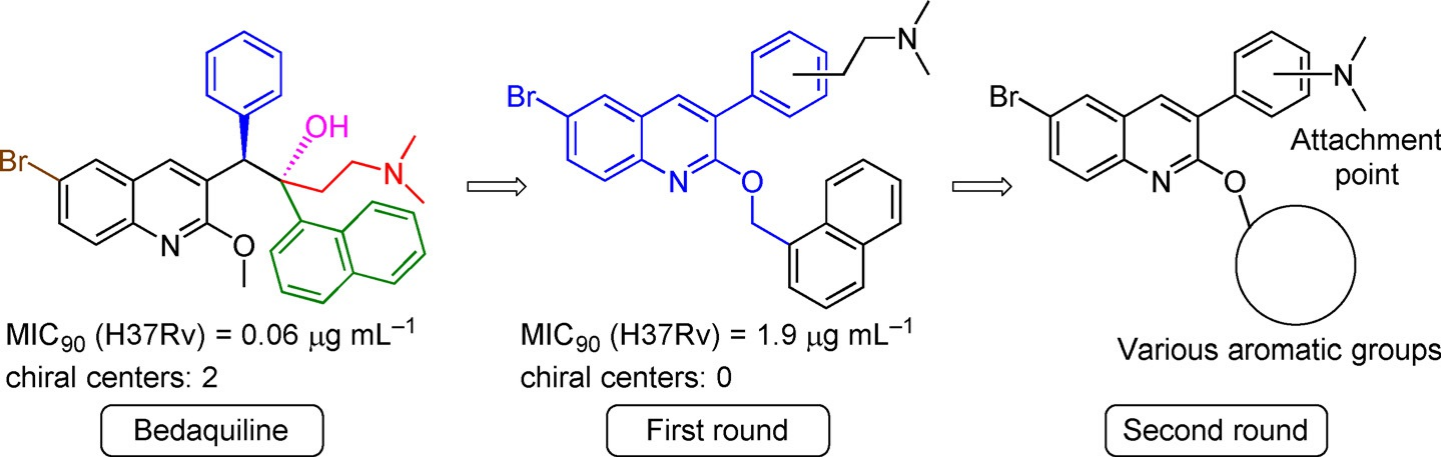
Scheme for the second round of compound optimization. Starting from bedaquiline, both chiral carbon atoms in the first round of optimization were removed, and a new scaffold of 2‐oxy‐3‐phenyl‐6‐bromoquinoline with potent anti‐TB activity was obtained. In the second round of optimization, the substituents of this new scaffold were changed to improve the anti‐TB activity of these new compounds.

Based on the results obtained from series 4, we explored the effect of 2‐aryl substituents and the spacer length of the terminal amine group in our new scaffold of 2‐aryl‐3‐(dimethylamino‐substituted)phenylquinoline on antitubercular activity (Figure 2).

**Scheme 6:**
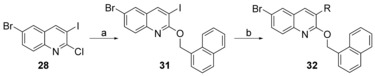
Synthesis of compound **32**. *Reagents and conditions*: a) 1‐naphthalenemethanol, NaH, THF, RT, 6 h, 93.4 %; b) for boric acid: Pd(PPh_3_)_4_, Na_2_CO_3_, toluene/H_2_O, 14 h, for boronic acid pinacol cyclic ester: Pd(dppf)Cl_2_⋅CH_2_Cl_2_, Na_2_CO_3_, toluene/H_2_O, 14 h, 41.4–86.8 %.

In the first group of compounds (Table 5), we investigated the position at which the basic nitrogen containing side chain was attached to the phenyl ring as well as the lateral chain length. We assembled a small library of 6‐bromo‐2‐((naphth‐1‐yl)methyl)oxy‐3‐substituted phenylquinoline analogues (**32**). The synthetic route is outlined in Scheme 6. The antitubercular screening assay showed that: 1) the *para*‐substituted *N*,*N*‐dimethylaminomethylphenyl ring has the most potent antitubercular activity for this scaffold, and 2) the terminal nitrogen atom is necessary for antitubercular activity. Other substituents decreased the antitubercular activity of this scaffold significantly. Compounds **32 a**, **32 d**, and **32 e** showed antitubercular activities (MIC) of 0.43, 0.47, and 0.44 μg mL^−1^, and cytotoxicity (CC_50_) against mammalian cells of 13, 12, and 3.9 μg mL^−1^, respectively.

**Table 5:** In vitro antitubercular activity of compounds from the second round of optimization.^[a]^

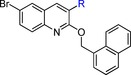						
Compd	R		MIC		CC_50_^[b]^	
			μg mL^−1^	μm	μg mL^−1^	μm
**32 a**	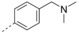		0.43	0.87	13	26.1
**32 b**	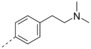		1.9	3.7	6.3	12.3
**32 c**	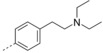		1.7	3.2	4.1	7.6
**32 d**	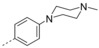		0.47	0.87	12	22.3
**32 e**	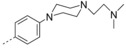		0.44	0.73	3.9	6.6
**32 f**	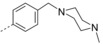		2.2	3.9	4.8	8.6
**32 g**	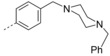		1.5	2.3	>64	>101.9
**32 h**	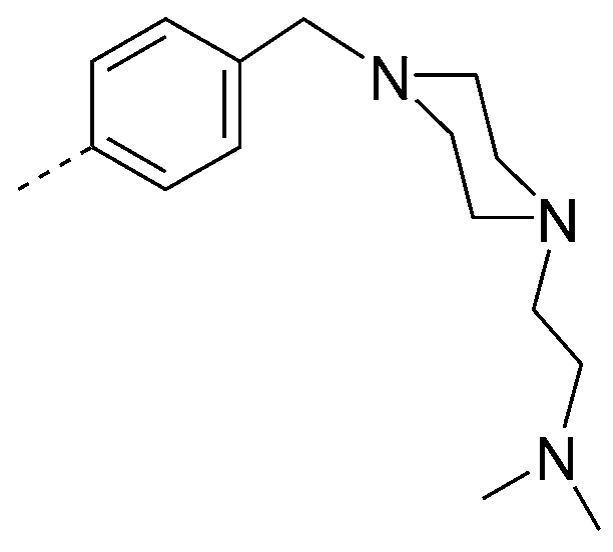		0.61	1.0	4.6	7.5
**32 i**	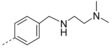		3.4	6.3	3.8	7.0
**32 j**	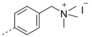		10	15.6	36	56.3
**32 k**	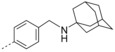		0.73	1.2	53	87.9
**32 l**	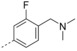		0.84	1.6	40	77.7
**32 m**	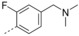		1.4	2.7	14	27.1
**32 n**	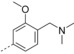		0.79	1.5	3.7	7.0
**32 o**	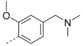		0.67	1.3	12	22.7
**32 p**	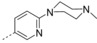		0.51	0.94	13	24.1
**32 q**	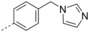		1.1	2.1	17	32.7
**32 r**	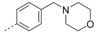		3.1	5.7	>64	>118.7
**32 s**	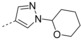		>32	>62.3	>64	>124.5
**32 t**	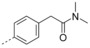		>32	>60.9	>64	>121.9
**32 u**	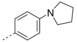		>32	>62.9	>64	>125.7
**32 v**	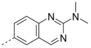		>32	>59.8	44	82.2

[a] Reference compound: bedaquiline MIC=0.060 μg mL^−1^ (0.1 μm). [b] Concentration at which the growth of Vero cells is inhibited by 50 %.

**Scheme 7:**
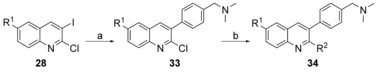
Synthesis of compound **34**. *Reagents and conditions*: a) (4‐((dimethylamino)methyl)phenyl)boronic acid, Pd(PPh_3_)_4_, Na_2_CO_3_, toluene/H_2_O, 14 h, 81.5–85.8 %; b) NaH, THF, RT, 6 h, 34.4–91.3 %.

Next, we substituted the naphthalen‐1‐ylmethanol ring with various aryl groups that have different physicochemical properties, while keeping 4‐(*N*,*N*‐dimethylaminomethyl)phenyl group as the 3‐substituent. A small group of analogues **34** were synthesized (Scheme 7). Antitubercular screening showed activity of several derivatives with MIC∼0.4 μg mL^−1^ (Table 6). The arylmethyloxy group at C2 of the quinolone was substituted with a variety of aromatic groups having different physicochemical properties, such as a *para*‐chlorophen‐1‐ylmethanol ring (**34 i**, MIC=0.55 μg mL^−1^) or a pyridin‐4‐ylmethanol ring (**34 m**, MIC=0.43 μg mL^−1^). These substituents maintain the antitubercular activity, and may provide a method for optimizing the physicochemical properties of the final compounds.

**Table 6:** In vitro antitubercular activity of compounds from the second round of optimization.^[a]^

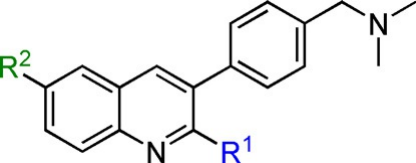							
Compd	R^1^	R^2^		MIC		CC_50_^[b]^	
				μg mL^−1^	μm	μg mL^−1^	μm
**34 a**	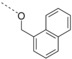	H		0.62	1.5	4.0	9.6
**34 b**	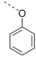	Br		1.6	3.7	11	25.4
**34 c**	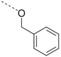	Br		2.5	5.6	7.8	17.4
**34 d**	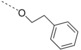	Br		2.0	4.3	12	26.0
**34 e**	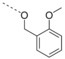	Br		0.81	1.7	6.2	12.9
**34 f**	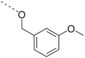	Br		0.58	1.2	4.4	9.2
**34 g**	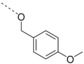	Br		5.2	10.9	5.6	11.7
**34 h**	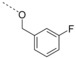	Br		0.72	1.5	7.4	15.9
**34 i**	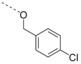	Br		0.55	1.1	11	22.9
**34 j**	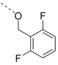	Br		1.5	3.1	4.2	8.7
**34 k**	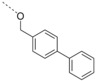	Br		0.89	1.7	14	26.8
**34 l**	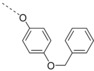	Br		0.79	1.5	5.0	9.3
**34 m**	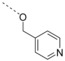	Br		0.43	0.95	3.0	6.7
**34 n**	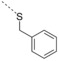	Br		0.71	1.5	9.7	20.9
**34 o**	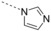	Br		1.6	3.9	12	29.4
**34 p**	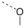	Br		5.9	15.9	17	45.8

[a] Reference compound: bedaquiline MIC=0.060 μg mL^−1^ (0.1 μm). [b] Concentration at which the growth of Vero cells is inhibited by 50 %.

### Inhibition of ATP synthesis activity by selected compounds in mycobacteria

**Figure 3:**
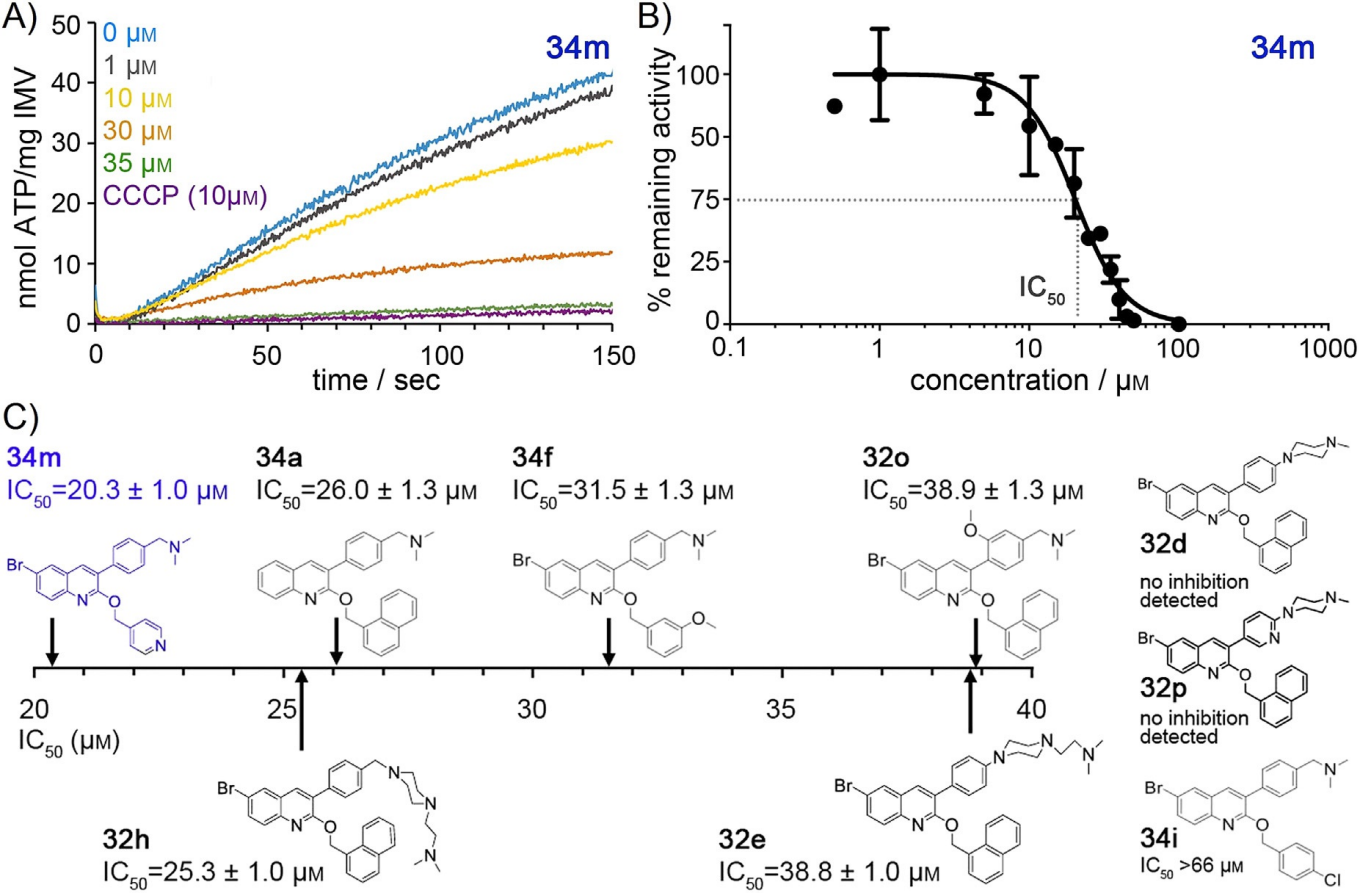
Inhibition of ATP synthesis in mycobacteria by selected lead compounds. The top nine candidates, selected by their MIC values, were tested for their ATP synthesis inhibitory activity using *Mycobacterium phlei* inverted membrane vesicles. The corresponding IC_50_ values were determined in a concentration‐dependent series of inhibition measurements. A) ATP synthesis activities were recorded using a luminescence‐based activity assay.^14^ The increasing luminescence signal, indicating continuous ATP synthesis at various concentrations (shown in different colors) of compound **34 m**, is shown exemplarily. As a control, the uncoupler carbonyl cyanide *m*‐chlorophenyl hydrazone (CCCP) is plotted (purple trace). B) Concentration‐dependent inhibition of ATP synthesis and determination of IC_50_ for **34 m**. The rates for ATP synthesis were calculated and plotted versus the concentration of compound **34 m** to determine the concentration required to inhibit 50 % of remaining activity (IC_50_). Error bars represent the standard deviation of at least three biological replicates. C) IC_50_ values determined for the tested compounds. Whereas six compounds showed inhibition of ATP synthesis activity with IC_50_ values between 20 and 40 μm (**34 m**, **32 h**, **34 a**, **34 f**, **32 e**, and **32 o**) and one >66 μm (**34 i**), two compounds (**32 d** and **32 p**) showed no measurable effect up to 100 μm.

The design of the new antimycobacterial lead compounds was inspired by bedaquiline, which is known to inhibit mycobacterial ATP synthase with an IC_50_ value in the low nanomolar range (Supporting Information Figure S1).^14^ To determine if the new compounds share this property, nine candidates with MIC values in the sub‐microgram per mL range were selected (Table S1) and tested for their ability to inhibit the ATP synthesis activity of *Mycobacterium phlei* inverted membrane vesicles (IMVs) (Figure 3 A). A concentration range of 0–100 μm was assayed for each compound. The calculated activities were used to determine an IC_50_ value for each of the candidates (Figure 3 B). Six of the compounds (**34 m**, **32 h**, **34 a**, **34 f**, **32 e**, and **32 o**) inhibited ATP synthesis with IC_50_ values between 20 and 40 μm (Figure S2). One candidate (**34 i**) showed a slight inhibitory effect; however, the IC_50_ value could not be calculated due to the low solubility of the compound. The best candidate was compound **34 m** with an IC_50_ value of 20.3±1 μm (Figure 3 C) and an improved log*P* of 5.55 (Table S1), which may also be beneficial from a pharmacokinetic point of view and which may help decrease some of the adverse side effects of bedaquiline.^8^, ^12^ Remarkably, two compounds (**32 d** and **32 p**) did not affect ATP synthesis at all, while still displaying potent antimycobacterial activity (MIC_90_: 0.47 and 0.51 μg mL^−1^, respectively; Figure S2). As a control, inhibition by bedaquiline was assayed, exhibiting an IC_50_ value <10 nm (Figure S1).

All seven tested compounds, which inhibited ATP synthesis activity, share a structural feature with bedaquiline, that is, the presence of a dimethylamino (DMA) moiety. This structural part of bedaquiline represents the most important structural feature in the interaction with the ATP synthase c‐ring and is hence a decisive element of the molecule's inhibitory power.^14^ In the lead compounds described herein, the DMA moieties are all attached to longer aryl side chains than the ethyl chain present in bedaquiline. Therefore, the subtle but exact structural placement of this critical moiety is impaired, which explains the higher IC_50_ values measured for these derivatives. In agreement with that notion are the two candidates (**32 d** and **32 p**), which show no influence on ATP synthesis activity and lack this structural element completely. The fact that these compounds still show specific killing activity on mycobacteria (Table S1) implies that they target and affect other cellular processes or proteins in mycobacterial cells. Future work needs to be carried out to identify these potentially interesting targets.

### In vitro anti‐TB activities against drug‐resistant clinical isolates of *M. tuberculosis*

To further explore the effect of selected compounds on drug‐resistant clinical isolates of *M. tuberculosis* and compare it with the effect on the drug‐sensitive H37Rv strain, we performed MIC determinations with two representative compounds that showed ATP synthesis inhibitory activity (**34 m** and **32 e**) and two candidates that showed only marginal and no effect on the ATP synthase, **34 i** and **32 d**, respectively (Figure 3 C). The in vitro inhibitory activities of these four compounds were screened against two drug‐resistant clinical isolates (isolates 12513 and 6133), which are both resistant to isoniazid (INH) and rifampicin (RFP).^28^ The MIC values against the drug‐sensitive H37Rv strain of *M. tuberculosis* were also determined again (Table 7). Compounds **34 m** and **32 e** exhibited similar MIC values for both the drug‐resistant and drug‐sensitive strains. Interestingly, for compound **34 i** that had only marginal effects on ATP synthesis, the MICs for the drug‐resistant strains 6133 and 12153 were 5.4‐ and 7.9‐fold higher. A similar effect was observed for compound **32 d**, which had no measureable impact on ATP synthesis: the MIC was found to be 7.4‐fold higher for strain 6133 than for the drug‐sensitive H37Rv strain. The measured effects for **34 i** and **32 d** indicate that these compounds target alternative sites or processes in the mycobacterial cell; these results encourage future work to identify these novel targets and to validate them in greater detail as potential new sites for mycobacterial inhibitors.

**Table 7:** In vitro antitubercular activities (MIC values) against drug‐resistant clinical isolates of *M. tuberculosis*.

Compd:	**34 m**			**32 e**			**34 i**			**32 d**			INH			RFP	
	μg mL^−1^	μm		μg mL^−1^	μm		μg mL^−1^	μm		μg mL^−1^	μm		μg mL^−1^	μm		μg mL^−1^	μm
12153^[a]^	0.48	1.07		0.94	1.57		2.62	5.45		0.51	0.94		>40	>291		2.11	2.56
6133^[a]^	0.55	1.22		0.69	1.15		1.83	3.81		3.42	6.35		>40	>291		>40	>48
H37R_V_	0.43	0.95		0.44	0.73		0.33	0.68		0.46	0.85		0.05	0.36		0.05	0.06

[a] Strain number of in vitro cultured clinical isolate of MDR *M. tuberculosis*.

## Conclusions

In this study we describe the chemical simplification of bedaquiline, while largely retaining the potent antitubercular activity. Inspired by the SAR of bedaquiline, we designed a set of new lead compounds using a fragment‐based approach. The bedaquiline molecule was first split into its various functional groups and recombined in a way that decreased or completely removed the initial stereochemical complexity. The approach promises to significantly decrease production costs and make it accessible for a broader variety of chemical laboratories and clinics. Initially, four series of compounds were designed, synthesized, and evaluated for their antitubercular activity. Among the initial four series of compounds, series 4, incorporating a 2‐aryl moiety and a 3‐(4‐(*N*,*N*‐dimethylamino)methyl)phenyl group, was found to contain the most potent analogues with MIC values <1 μg mL^−1^. Further optimization of this scaffold, modifying the C2 and C6 positions of the quinoline, yielded several potent inhibitors with MIC<0.6 μg mL^−1^. The most potent candidates were selected and assayed for their capacity to inhibit ATP synthesis activity in mycobacterial membranes. Our biochemical analysis reveals that some of the newly synthesized compounds showed a direct impact on mycobacterial ATP synthesis, with IC_50_ values between 20 and 40 μm, while others did not affect ATP synthesis itself. The latter compounds are promising new candidates that are able to target other cellular processes in mycobacterial cells. This study provides the basis for the development of novel, chemically simplified, low‐cost bedaquiline derivatives to fight drug‐resistant *M. tuberculosis* strains.

## Experimental Section

**Chemistry**: All reagents and anhydrous solvents are commercially available and were used without further purification. Temperatures are given in degrees Celsius (°C). NMR spectra were recorded on Bruker AVIIIHD spectrometers using TMS as internal standard. Chemical shifts (*δ*) are reported in ppm, and coupling constants (*J*) are given in hertz (Hz). The following multiplicity abbreviations are used: (s) singlet, (d) doublet, (t) triplet, (q) quartet, (m) multiplet, and (br) broad. ESI‐HRMS data were measured on a Thermo Exactive Orbitrap Plus spectrometer. All reactions were monitored by TLC. Column chromatography was carried out with silica gel (200–300 mesh size). Flash column chromatography was performed on a Biotage Isolera One instrument. Purity was determined by LC–MS and NMR spectroscopy. All final compounds are >95 % pure. See the Supporting Information for detailed synthesis and characterization of all intermediates and final compounds.

**6‐Bromo‐3‐(4‐((dimethylamino)methyl)phenyl)‐2‐(naphthalen‐1‐ylmethoxy)quinoline (32 a)**: A mixture of 6‐bromo‐3‐iodo‐2‐(naphthalen‐1‐ylmethoxy)quinoline **31** (100 mg, 0.20 mmol), tetrakis(triphenylphosphine)palladium (12 mg, 0.01 mmol), Na_2_CO_3_ (43 mg, 0.41 mmol) and 3‐((dimethylamino)methyl)phenylboronic acid (39 mg, 0.22 mmol) in the mixture of toluene (4 mL) and water (2 mL) was stirred at 80 °C for 10 h. Water (10 mL) was added and the mixture was extracted with CH_2_Cl_2_ (3×10 mL), dried with Na_2_SO_4_ and concentrated. The residue was purified by flash column chromatography with petroleum ether/ethyl acetate/triethylamine (40:15:1) to afford **32 a** as a white solid (87 mg, 87.3 %); mp: 85–86 °C. ^1^H NMR (400 MHz, [D_6_]acetone): *δ*=8.23 (d, *J*=8.2 Hz, 1 H), 8.18 (s, 1 H), 8.11 (s, 1 H), 7.94 (d, *J*=7.8 Hz, 1 H), 7.88 (d, *J*=8.4 Hz, 1 H), 7.85 (d, *J*=8.9 Hz, 1 H), 7.78 (dd, *J=*8.8, 1.6 Hz, 1 H), 7.72 (d, *J*=6.9 Hz, 1 H), 7.61–7.56 (m, 3 H), 7.54 (t, *J=*6.8 Hz, 1 H), 7.46 (t, *J*=7.6 Hz, 1 H), 7.29 (d, *J*=7.9 Hz, 2 H), 6.07 (s, 2 H), 3.38 (s, 2 H), 2.17 ppm (s, 6 H); ^13^C NMR (100 MHz, [D_6_]acetone): *δ*=160.22, 145.21, 140.31, 138.10, 135.47, 134.74, 133.66, 133.26, 132.68, 130.60, 130.07, 129.66, 129.64, 129.43, 129.33, 128.30, 128.11, 128.00, 127.13, 126.73, 126.15, 124.94, 117.89, 67.02, 64.33, 45.56 ppm; HRMS (ESI‐TOF, *m*/*z*): calcd for C_29_H_26_ON_2_Br [*M*+H]^+^ 497.1223, found: 497.1223.

**6‐Bromo‐3‐(4‐(4‐methylpiperazin‐1‐yl)phenyl)‐2‐(naphthalen‐1‐ylmethoxy)quinoline (32 d)**: The procedure used to synthesize **32 a** was repeated using **31** and (4‐(4‐methylpiperazin‐1‐yl)phenyl)boronic acid to afford compound **32 d** as a white solid in 79.1 % yield; mp: 132–133 °C. ^1^H NMR (400 MHz, [D_6_]acetone): *δ*=8.26 (d, *J=*8.5 Hz, 1 H), 8.14 (s, 1 H), 8.10 (d, *J=*2.2 Hz, 1 H), 7.96 (d, *J=*7.6 Hz, 1 H), 7.91 (d, *J=*8.3 Hz, 1 H), 7.84 (d, *J=*8.9 Hz, 1 H), 7.80–7.71 (m, 2 H), 7.65–7.59 (m, 1 H), 7.59–7.52 (m, 3 H), 7.49 (dd, *J=*8.2, 7.1 Hz, 1 H), 6.95–6.85 (m, 2 H), 6.09 (s, 2 H), 3.24–3.13 (m, 4 H),2.51–2.41 (m, 4 H), 2.24 ppm (s, 3 H); ^13^C NMR (100 MHz, [D_6_]acetone): *δ*=160.43, 152.09, 144.80, 136.96, 134.77, 133.80, 132.80, 132.73, 131.02, 130.39, 129.62, 129.61, 129.46, 128.44, 128.25, 128.15, 127.19, 126.81, 126.75, 126.22, 124.95, 117.79, 115.59, 66.92, 55.79, 48.99, 46.39 ppm; HRMS (ESI‐TOF, *m*/*z*): calcd for C_31_H_29_ON_3_Br [*M*+H]^+^ 538.1485, found 538.1482.

**6‐Bromo‐3‐(4‐((4‐(2‐(dimethylamino)ethyl)piperazin‐1‐yl)methyl)phenyl)‐2‐(naphthalen‐1‐ylmethoxy)quinoline (32 h)**: The procedure used to synthesize **32 a** was repeated using **31** and (4‐((4‐(2‐(dimethylamino)ethyl)piperazin‐1‐yl)methyl)phenyl)boronic acid to afford compound **32 h** as a yellow wax‐like solid in 67.2 % yield; mp: 95–96 °C. ^1^H NMR (400 MHz, [D_6_]acetone): *δ*=8.24 (d, *J*=8.2 Hz, 1 H), 8.20 (s, 1 H), 8.12 (s, 1 H), 7.95 (d, *J*=7.8 Hz, 1 H), 7.89 (d, *J*=8.3 Hz, 1 H), 7.86 (d, *J*=9.0 Hz, 1 H), 7.79 (d, *J*=8.9 Hz, 1 H), 7.73 (d, *J*=6.9 Hz, 1 H), 7.66–7.50 (m, 4 H), 7.47 (t, *J*=7.7 Hz, 1 H), 7.30 (d, *J*=7.9 Hz, 2 H), 6.08 (s, 2 H), 3.45 (s, 2 H), 2.63–2.27 (m, 12 H), 2.16 ppm (s, 6 H); ^13^C NMR (150 MHz, [D_6_]acetone): *δ*=160.26, 145.24, 139.71, 138.14, 135.50, 134.77, 133.68, 133.30, 132.71, 130.62, 130.11, 129.69, 129.68, 129.45, 128.35, 128.15, 128.03, 127.15, 126.76, 126.18, 124.99, 117.92, 67.05, 63.11, 58.02, 57.42, 54.47, 54.03, 46.09 ppm; HRMS (ESI‐TOF, *m*/*z*): calcd for C_35_H_38_ON_4_^81^Br [*M*+H]^+^ 611.2203, found 611.2185.

**6‐Bromo‐3‐(4‐(((3s,5s,7s)‐adamantan‐1‐ylamino)methyl)phenyl)‐2‐(naphthalen‐1‐ylmethoxy)quinoline (32 k)**: The procedure used to synthesize **32 a** was repeated using **31** and (4‐(((3s,5s,7s)‐adamantan‐1‐ylamino)methyl)phenyl)boronic acid to afford compound **32 k** as a yellow solid in 55.1 % yield; mp: 72–73 °C. ^1^H NMR (400 MHz, [D_6_]acetone): *δ*=8.25 (d, *J*=8.1 Hz, 1 H), 8.18 (s, 1 H), 8.12 (d, *J*=1.6 Hz, 1 H), 7.95 (d, *J*=8.0 Hz, 1 H), 7.90 (d, *J*=8.4 Hz, 1 H), 7.86 (d, *J*=8.9 Hz, 1 H), 7.78 (dd, *J*=8.8, 1.8 Hz, 1 H), 7.74 (d, *J*=6.8 Hz, 1 H), 7.61 (t, *J*=7.6 Hz, 1 H), 7.58–7.51 (m, 3 H), 7.48 (t, *J*=7.6 Hz, 1 H), 7.34 (d, *J*=7.9 Hz, 2 H), 6.08 (s, 2 H), 3.74 (s, 2 H), 2.77 (br s, 1 H), 1.92–1.45 ppm (m, 15 H); ^13^C NMR (150 MHz, [D_6_]acetone): *δ*=160.33, 145.20, 143.69, 138.07, 134.90, 134.79, 133.70, 133.24, 132.76, 130.60, 129.97, 129.70, 129.69, 129.47, 128.64, 128.50, 128.26, 128.06, 127.20, 126.78, 126.21, 124.98, 117.90, 67.07, 51.12, 45.09, 43.65, 37.56, 30.59 ppm; HRMS (ESI‐TOF, *m*/*z*): calcd for C_37_H_36_ON_2_^81^Br [*M*+H]^+^ 605.1985, found 605.1.992

**6‐Bromo‐3‐(4‐((dimethylamino)methyl)‐3‐fluorophenyl)‐2‐(naphthalen‐1‐ylmethoxy)quinoline (32 l)**: The procedure used to synthesize **32 b** was repeated using **31** and (4‐((dimethylamino)methyl)‐3‐fluorophenyl)boronic acid pinacol ester to afford compound **32 l** as a white solid in 71.4 % yield; mp: 106–107 °C. ^1^H NMR (400 MHz, [D_6_]acetone): *δ*=8.30–8.21 (m, 2 H), 8.14 (s, 1 H), 7.96 (d, *J*=7.8 Hz, 1 H), 7.91 (d, *J*=8.3 Hz, 1 H), 7.88 (d, *J*=9.2 Hz, 1 H), 7.82 (d, *J*=8.9 Hz, 1 H), 7.75 (d, *J*=6.9 Hz, 1 H), 7.60 (t, *J*=6.9 Hz, 1 H), 7.55 (t, *J*=6.9 Hz, 1 H), 7.51–7.44 (m, 2 H), 7.43–7.34 (m, 2 H), 6.10 (s, 2 H), 3.44 (s, 2 H), 2.19 ppm (s, 6 H); ^13^C NMR (150 MHz, [D_6_]acetone): *δ*=162.47, 160.85, 159.97, 145.43, 138.56, 137.69, 137.63, 134.80, 133.65, 133.56, 132.76, 131.95, 131.92, 130.77, 129.79, 129.72, 129.48, 128.33, 127.88, 127.26, 126.97, 126.96, 126.79, 126.53, 126.43, 126.18, 125.88, 125.86, 124.94, 118.05, 117.01, 116.85, 67.20, 56.63, 56.62, 45.45 ppm; HRMS (ESI‐TOF, *m*/*z*): calcd for C_29_H_25_ON_2_^81^BrF [*M*+H]^+^ 517.1108, found 517.1117.

**6‐Bromo‐3‐(4‐((dimethylamino)methyl)‐3‐methoxyphenyl)‐2‐(naphthalen‐1‐ylmethoxy)quinoline (32 n)**: The procedure used to synthesize **32 b** was repeated using **31** and (4‐((dimethylamino)methyl)‐3‐methoxyphenyl)boronic acid pinacol ester to afford compound **32 n** as a white solid in 78.5 % yield; mp: 106–107 °C. ^1^H NMR (600 MHz, [D_6_]acetone): *δ*=8.26 (s, 1 H), 8.23 (d, *J*=8.3 Hz, 1 H), 8.14 (d, *J*=2.2 Hz, 1 H), 7.97 (d, *J*=7.5 Hz, 1 H), 7.93 (d, *J*=8.3 Hz, 1 H), 7.88 (d, *J*=8.8 Hz, 1 H), 7.80 (dd, *J*=8.8, 2.3 Hz, 1 H), 7.75 (d, *J*=6.8 Hz, 1 H), 7.62–7.57 (m, 1 H), 7.57–7.53 (m, 1 H), 7.49 (dd, *J*=8.2, 7.0 Hz, 1 H), 7.34 (d, *J*=7.6 Hz, 1 H), 7.22–7.15 (m, 2 H), 6.06 (s, 2 H), 3.35 (s, 2 H), 3.27 (s, 3 H), 2.16 ppm (s, 6 H); ^13^C NMR (150 MHz, [D_6_]acetone): *δ*=160.26, 157.97, 145.24, 138.11, 136.43, 134.82, 133.66, 133.33, 132.91, 130.67, 130.46, 129.90, 129.69, 129.53, 128.70, 128.21, 128.07, 128.04, 127.44, 126.80, 126.27, 124.92, 121.98, 117.92, 112.64, 67.25, 57.55, 55.24, 45.74 ppm; HRMS (ESI‐TOF, *m*/*z*): calcd for C_30_H_28_O_2_N_2_Br [*M*+H]^+^ 527.1329, found 527.1323.

**6‐Bromo‐3‐(4‐((dimethylamino)methyl)‐2‐methoxyphenyl)‐2‐(naphthalen‐1‐ylmethoxy)quinoline (32 o)**: The procedure used to synthesize **32 b** was repeated using **31** and (4‐((dimethylamino)methyl)‐2‐methoxyphenyl)boronic acid pinacol ester to afford compound **32 o** as a yellow solid in 71.8 % yield; mp: 65–66 °C. ^1^H NMR (600 MHz, [D_6_]acetone): *δ*=8.12 (dd, *J*=8.0, 1.0 Hz, 1 H), 8.08 (d, *J*=2.2 Hz, 1 H), 8.04 (s, 1 H), 7.91 (dd, *J*=7.5, 1.9 Hz, 1 H), 7.85 (d, *J*=6.1 Hz, 1 H), 7.84 (d, *J*=6.7 Hz, 1 H), 7.77 (dd, *J*=8.9, 2.3 Hz, 1 H), 7.63 (dd, *J*=6.9, 0.6 Hz, 1 H), 7.57–7.49 (m, 2 H), 7.42 (dd, *J*=8.2, 7.1 Hz, 1 H), 7.23 (d, *J*=7.5 Hz, 1 H), 6.98 (s, 1 H), 6.92–6.86 (m, 1 H), 3.56 (s, 3 H), 3.39 (s, 2 H), 2.19 ppm (s, 6 H); ^13^C NMR (150 MHz, [D_6_]acetone): *δ*=161.08, 158.17, 145.43, 142.42, 138.88, 134.66, 133.89, 133.09, 132.58, 131.46, 130.44, 129.72, 129.36, 129.33, 127.76, 127.42, 127.06, 126.62, 126.50, 126.12, 124.88, 124.83, 121.30, 117.64, 111.83, 66.56, 64.71, 55.65, 45.62 ppm; HRMS (ESI‐TOF, *m*/*z*): calcd for C_30_H_28_O_2_N_2_Br [*M*+H]^+^ 527.1329, found 527.1325.

**6‐Bromo‐3‐(6‐(4‐methylpiperazin‐1‐yl)pyridin‐3‐yl)‐2‐(naphthalen‐1‐ylmethoxy)quinoline (32 p)**: The procedure used to synthesize **32 b** was repeated using **31** and (6‐(4‐methylpiperazin‐1‐yl)pyridin‐3‐yl)boronic acid pinacol ester to afford compound **32 p** as a white solid in 81.2 % yield; mp: 130–131 °C. ^1^H NMR (400 MHz, [D_6_]acetone): *δ*=8.42 (d, *J*=1.8 Hz, 1 H), 8.23 (d, *J*=8.2 Hz, 1 H), 8.18 (s, 1 H), 8.10 (d, *J*=1.8 Hz, 1 H), 7.96 (d, *J*=7.8 Hz, 1 H), 7.91 (d, *J*=8.3 Hz, 1 H), 7.87–7.79 (m, 2 H), 7.79–7.70 (m, 2 H), 7.64–7.52 (m, 2 H), 7.49 (t, *J*=7.6 Hz, 1 H), 6.70 (d, *J*=8.9 Hz, 1 H), 6.09 (s, 2 H), 3.53 (t, *J*=5.0 Hz, 4 H), 2.39 (t, *J*=5.0 Hz, 4 H), 2.23 ppm (s, 3 H); ^13^C NMR (150 MHz, [D_6_]acetone): *δ*=160.38, 159.64, 149.00, 144.96, 139.05, 136.75, 134.79, 133.67, 133.01, 132.78, 130.44, 129.76, 129.66, 129.50, 128.32, 128.18, 127.28, 126.79, 126.24, 125.84, 124.85, 121.44, 117.92, 106.54, 67.06, 55.58, 46.43, 45.55 ppm; HRMS (ESI‐TOF, *m*/*z*): calcd for C_30_H_28_^81^BrN_4_O [*M*+H]^+^ 541.1421, found 541.1427.

**3‐(4‐((Dimethylamino)methyl)phenyl)‐2‐(naphthalen‐1‐ylmethoxy)quinoline (34 a)**: To a solution of 2‐chloro‐3‐(4‐((dimethylamino)methyl)phenyl)quinoline (100 mg, 0.34 mmol) and 1‐naphthalenemethanol (68 mg, 0.43 mmol) in 4 mL DMF was added Cs_2_CO_3_ (140 mg, 0.43 mmol). After stirring at 80 °C for 8 h, water (20 mL) was added, and the mixture was extracted with CH_2_Cl_2_ (3×10 mL), dried with Na_2_SO_4_ and concentrated. The residue was purified by flash column chromatography with petroleum ether/ethyl acetate/triethylamine (40:15:1) to afford **34 a** as a white solid (128.9 mg, 91.2 %); mp: 93–95 °C. ^1^H NMR (600 MHz, [D_6_]acetone): *δ*=8.25 (d, *J*=8.4 Hz, 1 H), 8.21 (s, 1 H), 7.98–7.89 (m, 3 H), 7.88 (d, *J*=8.3 Hz, 1 H), 7.74 (d, *J*=6.9 Hz, 1 H), 7.69 (t, *J*=7.6 Hz, 1 H), 7.65–7.58 (m, 3 H), 7.55 (t, *J*=7.4 Hz, 1 H), 7.46 (t, *J*=7.6 Hz, 2 H), 7.29 (d, *J*=7.9 Hz, 2 H), 6.09 (s, 2 H), 3.39 (s, 2 H), 2.17 ppm (s, 6 H); ^13^C NMR (150 MHz, [D_6_]acetone): *δ*=159.80, 146.56, 139.94, 139.07, 136.01, 134.76, 133.94, 132.73, 130.31, 130.09, 129.56, 129.43, 129.32, 128.62, 128.04, 127.65, 127.22, 127.11, 126.72, 126.70, 126.18, 125.30, 124.99, 66.77, 64.35, 45.55 ppm; HRMS (ESI‐TOF, *m*/*z*): calcd for C_29_H_27_N_2_O [*M*+H]^+^ 419.2118, found 419.2110.

**6‐Bromo‐3‐(4‐((dimethylamino)methyl)phenyl)‐2‐((2‐methoxybenzyl)oxy)quinoline (34 e)**: The procedure used to synthesize **34 a** was repeated using **33 a** and 2‐methoxybenzyl alcohol to afford compound **34 e** as a yellow wax‐like solid in 81.5 % yield; mp: 96–97 °C. ^1^H NMR (600 MHz, [D_6_]acetone): *δ*=8.20 (s, 1 H), 8.11 (d, *J*=2.2 Hz, 1 H), 7.81 (d, *J*=8.8 Hz, 1 H), 7.76 (dd, *J*=8.9, 2.2 Hz, 1 H), 7.73–7.64 (m, 2 H), 7.44 (dd, *J*=7.5, 1.4 Hz, 1 H), 7.40 (d, *J*=8.2 Hz, 2 H), 7.29 (td, *J*=8.2, 1.7 Hz, 1 H), 7.03 (d, *J*=8.1 Hz, 1 H), 6.90 (td, *J*=7.4, 0.7 Hz, 1 H), 5.60 (s, 2 H), 3.91 (s, 3 H), 3.44 (s, 2 H), 2.21 ppm (s, 6 H); ^13^C NMR (150 MHz, [D_6_]acetone): *δ*=160.48, 158.37, 145.31, 140.42, 137.92, 135.64, 133.20, 130.59, 130.14, 129.93, 129.87, 129.64, 129.41, 128.28, 127.97, 126.10, 121.04, 117.77, 111.32, 64.49, 64.44, 55.81, 45.61 ppm; HRMS (ESI‐TOF, *m*/*z*): calcd for C_26_H_26_BrN_2_O_2_ [*M*+H]^+^ 477.1172, found 477.1166.

**6‐Bromo‐3‐(4‐((dimethylamino)methyl)phenyl)‐2‐((3‐methoxybenzyl)oxy)quinoline (34 f)**: The procedure used to synthesize **34 a** was repeated using **33 a** and 3‐methoxybenzyl alcohol to afford compound **34 f** as a colorless wax‐like solid in 92.4 % yield; mp: 52–53 °C. ^1^H NMR (600 MHz, [D_6_]acetone): *δ*=8.20 (s, 1 H), 8.11 (d, *J*=2.1 Hz, 1 H), 7.80 (d, *J*=8.8 Hz, 1 H), 7.76 (dd, *J*=8.9, 2.2 Hz, 1 H), 7.69 (d, *J*=8.2 Hz, 2 H), 7.42 (d, *J*=8.2 Hz, 2 H), 7.26 (t, *J*=7.9 Hz, 1 H), 7.09 (d, *J*=1.7 Hz, 1 H), 7.06 (d, *J*=7.5 Hz, 1 H), 6.84 (dd, *J*=8.1, 2.4 Hz, 1 H), 5.58 (s, 2 H), 3.76 (s, 3 H), 3.45 (s, 2 H), 2.22 ppm (s, 6 H); ^13^C NMR (150 MHz, [D_6_]acetone): *δ*=160.75, 160.22, 145.22, 140.45, 139.79, 138.06, 135.60, 133.26, 130.60, 130.20, 130.14, 129.61, 129.46, 128.24, 127.98, 120.55, 117.88, 114.23, 113.68, 68.46, 64.42, 55.45, 45.61 ppm; HRMS (ESI‐TOF, *m*/*z*): calcd for C_26_H_26_BrN_2_O_2_ [*M*+H]^+^ 477.1172, found 477.1167.

**6‐Bromo‐3‐(4‐((dimethylamino)methyl)phenyl)‐2‐((3‐fluorobenzyl)oxy)quinoline (34 h)**: The procedure used to synthesize **34 a** was repeated using **33 a** and 3‐fluorobenzyl alcohol to afford compound **34 h** as a yellow solid in 85.4 % yield; mp: 82–83 °C. ^1^H NMR (600 MHz, [D_6_]acetone): *δ*=8.21 (s, 1 H), 8.12 (d, *J*=2.1 Hz, 1 H), 7.79 (d, *J*=8.8 Hz, 1 H), 7.76 (dd, *J*=8.9, 2.1 Hz, 1 H), 7.71–7.65 (m, 2 H), 7.43 (d, *J*=8.3 Hz, 2 H), 7.39 (td, *J*=7.9, 5.9 Hz, 1 H), 7.33 (d, *J*=7.7 Hz, 1 H), 7.30–7.24 (m, 1 H), 7.09–7.01 (m, 1 H), 5.62 (s, 2 H), 3.46 (s, 2 H), 2.22 ppm (s, 6 H); ^13^C NMR (150 MHz, [D_6_]acetone): *δ*=164.48, 162.86, 160.04, 145.14, 141.25, 141.20, 140.52, 138.17, 135.50, 133.33, 131.10, 131.04, 130.63, 130.13, 129.62, 129.48, 128.21, 128.04, 124.33, 124.31, 118.00, 115.27, 115.19, 115.12, 115.05, 67.79, 67.78, 64.42, 45.60 ppm; HRMS (ESI‐TOF, *m*/*z*): calcd for C_25_H_23_BrFN_2_O [*M*+H]^+^ 465.0972, found 465.0974.

**6‐Bromo‐2‐((4‐chlorobenzyl)oxy)‐3‐(4‐((dimethylamino)methyl)phenyl)quinoline (34 i)**: The procedure used to synthesize **34 a** was repeated using **33 a** and 4‐chlorobenzyl alcohol to afford compound **34 i** as a yellow solid in 82.4 % yield; mp: 120–121 °C. ^1^H NMR (600 MHz, [D_6_]acetone): *δ*=8.21 (s, 1 H), 8.12 (d, *J*=2.0 Hz, 1 H), 7.79 (d, *J*=8.8 Hz, 1 H), 7.76 (dd, *J*=8.9, 2.1 Hz, 1 H), 7.70–7.64 (m, 2 H), 7.56–7.52 (m, 2 H), 7.42 (d, *J*=8.2 Hz, 2 H), 7.40–7.35 (m, 2 H), 5.59 (s, 2 H), 3.45 (s, 2 H), 2.22 ppm (s, 6 H); ^13^C NMR (150 MHz, [D_6_]acetone): *δ*=160.08, 145.14, 140.53, 138.16, 137.29, 135.50, 133.83, 133.32, 130.63, 130.55, 130.11, 129.61, 129.48, 129.25, 128.19, 128.03, 117.97, 67.83, 64.43, 45.63 ppm; HRMS (ESI‐TOF, *m*/*z*): calcd for C_25_H_23_^81^BrClN_2_O [*M*+H]^+^ 483.0656, found 483.0639.

**2‐([1,1′‐Biphenyl]‐4‐ylmethoxy)‐6‐bromo‐3‐(4‐((dimethylamino)methyl)phenyl)quinoline (34 k)**: The procedure used to synthesize **34 a** was repeated using **33 a** and [1,1′‐biphenyl]‐4‐ylmethanol to afford compound **34 k** as a yellow solid in 89.8 % yield; mp: 96–97 °C. ^1^H NMR (600 MHz, [D_6_]acetone): *δ*=8.22 (s, 1 H), 8.13 (d, *J*=2.2 Hz, 1 H), 7.82 (d, *J*=8.9 Hz, 1 H), 7.77 (dd, *J*=8.9, 2.2 Hz, 1 H), 7.70 (d, *J*=8.2 Hz, 2 H), 7.68–7.63 (m, 4 H), 7.61 (d, *J*=8.3 Hz, 2 H), 7.48–7.43 (t, *J*=7.8 Hz, 2 H), 7.42 (d, *J*=8.2 Hz, 2 H), 7.35 (t, *J*=7.4 Hz, 1 H), 5.66 (s, 2 H), 3.45 (s, 2 H), 2.21 ppm (s, 6 H); ^13^C NMR (150 MHz, [D_6_]acetone): *δ*=160.27, 145.22, 141.46, 141.26, 140.48, 138.11, 137.44, 135.59, 133.28, 130.62, 130.14, 129.70, 129.63, 129.47, 129.38, 128.25, 128.21, 128.02, 127.71, 127.69, 117.89, 68.36, 64.44, 45.62 ppm; HRMS (ESI‐TOF, *m*/*z*): calcd for C_31_H_28_ON_2_Br [*M*+H]^+^ 523.1380, found 523.1389.

**2‐(4‐(Benzyloxy)phenoxy)‐6‐bromo‐3‐(4‐((dimethylamino)methyl)phenyl)quinoline (34 l)**: The procedure used to synthesize **34 a** was repeated using **33 a** and 4‐(benzyloxy)phenol to afford compound **34 l** as a yellow solid in 79.9 % yield; mp: 103–104 °C. ^1^H NMR (600 MHz, [D_6_]acetone): *δ*=8.31 (s, 1 H), 8.15 (s, 1 H), 7.77 (d, *J*=7.8 Hz, 2 H), 7.71 (dd, *J*=8.9, 1.3 Hz, 1 H), 7.55 (d, *J*=8.9 Hz, 1 H), 7.51 (d, *J*=7.6 Hz, 2 H), 7.46 (d, *J*=7.9 Hz, 2 H), 7.41 (t, *J*=7.5 Hz, 2 H), 7.34 (t, *J*=7.3 Hz, 1 H), 7.20 (d, *J*=8.8 Hz, 2 H), 7.08 (d, *J*=8.8 Hz, 2 H), 5.14 (s, 2 H), 3.47 (s, 2 H), 2.22 ppm (s, 6 H); ^13^C NMR (150 MHz, [D_6_]acetone): *δ*=160.75, 156.90, 148.20, 144.98, 140.61, 138.87, 138.41, 135.53, 133.41, 130.58, 130.17, 129.79, 129.61, 129.29, 128.65, 128.49, 128.36, 123.79, 118.44, 116.20, 70.85, 64.43, 45.64 ppm; HRMS (ESI‐TOF, *m*/*z*): calcd for C_31_H_28_BrN_2_O_2_ [*M*+H]^+^ 539.1329, found 539.1334.

**6‐Bromo‐3‐(4‐((dimethylamino)methyl)phenyl)‐2‐(pyridin‐4‐ylmethoxy)quinoline (34 m)**: The procedure used to synthesize **34 a** was repeated using **33 a** and 4‐pyridylcarbinol to afford compound **34 m** as a white solid in 84.7 % yield; mp: 86–87 °C. ^1^H NMR (600 MHz, [D_6_]acetone): *δ*=8.54 (dd, *J*=4.4, 1.5 Hz, 2 H), 8.24 (s, 1 H), 8.13 (s, 1 H), 7.76 (d, *J*=1.3 Hz, 2 H), 7.71 (d, *J*=8.2 Hz, 2 H), 7.47 (d, *J*=8.0 Hz, 2 H), 7.44 (d, *J*=5.9 Hz, 2 H), 5.65 (s, 2 H), 3.49 (s, 2 H), 2.24 ppm (s, 6 H); ^13^C NMR (150 MHz, [D_6_]acetone): *δ*=159.83, 150.65, 147.16, 145.07, 140.45, 138.30, 135.47, 133.38, 130.65, 130.16, 129.62, 129.58, 128.17, 128.09, 122.65, 118.11, 66.97, 64.37, 45.55 ppm; HRMS (ESI‐TOF, *m*/*z*): calcd for C_24_H_23_BrN_3_O [*M*+H]^+^ 448.1019, found 448.1016.

**2‐(Benzylthio)‐6‐bromo‐3‐(4‐((dimethylamino)methyl)phenyl)quinoline (34 n)**: The procedure used to synthesize **34 a** was repeated using **33 a** and benzyl mercaptan to afford compound **34 n** as a yellow solid in 71.4 % yield; mp: 67–68 °C. ^1^H NMR (600 MHz, [D_6_]acetone): *δ*=8.13 (s, 1 H), 7.98 (d, *J*=8.9 Hz, 1 H), 7.96 (s, 1 H), 7.83 (d, *J*=8.9 Hz, 1 H), 7.49 (d, *J*=7.8 Hz, 2 H), 7.47 (d, *J*=8.0 Hz, 2 H), 7.43 (d, *J*=7.9 Hz, 2 H), 7.28 (t, *J*=7.5 Hz, 2 H), 7.20 (t, *J*=7.3 Hz, 1 H), 4.56 (s, 2 H), 3.46 (s, 2 H), 2.21 ppm (s, 6 H); ^13^C NMR (150 MHz, [D_6_]acetone): *δ*=160.10, 146.51, 141.09, 139.18, 136.75, 136.21, 135.16, 133.62, 130.81, 130.39, 130.21, 130.04, 129.67, 129.19, 128.33, 127.81, 119.27, 64.41, 45.64, 35.22 ppm; HRMS (ESI‐TOF, *m*/*z*): calcd for C_25_H_24_BrN_2_S [*M*+H]^+^ 463.0844, found 463.0844.

**Minimum inhibitory concentration and cytotoxicity assays**: The assays were performed following our previously published protocols.^28^, ^29^, ^30^

**Luminescence‐based ATP synthesis inhibition assays and calculation of IC_50_ values**: *Mycobacterium phlei* cells were grown and inverted membrane vesicles (IMVs) were prepared as described.^14^ IMVs were frozen and stored in liquid nitrogen until further use. To assay ATP synthesis activity, an aliquot of IMVs were thawed on ice, and 4 mm malonate was added. After 1 h of incubation, 25 μg total protein in the IMVs were mixed with 270 μL reaction buffer (20 mm Tricine/KOH pH 7.5, 0.1 m NaCl, 5 mm KPi, 5 mm MgCl_2_), 50 μm ADP, 50 ng luciferase (Roche), 166 μm luciferin, and 2 μL DMSO to a total volume of 300 μL. The addition of inhibitors was varied in the concentration range of 0.5–100 μm. After incubation for 10 min at room temperature the ATP synthesis reaction was started by the addition of 10 mm succinate (pH 7.8). The synthesis of ATP was monitored by an increasing luminescence signal using a Sirius L single‐tube Luminometer (Berthold). As an internal standard, 8 nm ATP was added after each reaction. The data were evaluated and the inhibitory concentrations of 50 % (IC_50_) were calculated using Microsoft Excel and GraphPad Prism (version 7.0a).

**Notes**: The authors declare no competing financial interest.

## Supporting information

As a service to our authors and readers, this journal provides supporting information supplied by the authors. Such materials are peer reviewed and may be re‐organized for online delivery, but are not copy‐edited or typeset. Technical support issues arising from supporting information (other than missing files) should be addressed to the authors.

Supplementary
