# Supplementary material for: Structural Simplification of Bedaquiline: the Discovery of 3‐(4‐(N,N‐Dimethylaminomethyl)phenyl)quinoline‐Derived Antitubercular Lead Compounds
Source: ChemMedChem. 2016 Oct 28;12(2):106–19. doi: 10.1002/cmdc.201600441 (PMC5298006; doi:10.1002/cmdc.201600441)
Supplement: Supplementary file 1 — Supplementary [file CMDC-12-106-s001.pdf]

CHEMMEDCHEM
Chemistry & Drug Discovery

Supporting Information

Structural Simplification of Bedaquiline: the Discovery of 3-(4-(*N*,*N*-Dimethylaminomethyl)phenyl)quinoline-Derived Antitubercular Lead Compounds

Chunxian He,[a, b] Laura Preiss,[c] Bin Wang,[d] Lei Fu,[d] Hui Wen,[b] Xiang Zhang,[b] Huaqing Cui,*[b] Thomas Meier,*[c, e] and Dali Yin*[a]

cmdc_201600441_sm_miscellaneous_information.pdf

TABLE OF CONTENT

SUPPLEMENTARY MATERIALS AND METHODS

- Bacterial strains
- MIC Determination

SUPPLEMENTARY FIGURES AND TABLES

- Figure S1: Concentration dependent inhibition of mycobacterial ATP synthesis by bedaquiline measured in *Mycobacterium phlei* inverted membrane vesicles.
- Figure S2: Concentration dependent inhibition of mycobacterial ATP synthesis by selected lead compounds measured in *Mycobacterium phlei* inverted membrane vesicles.
- Table S1: Chemical and biological characteristics of the most potent lead compounds described in this work.

CHEMICAL SYNTHESES AND CHARACTERIZATION

- General Information
- Schemes: Synthesis and Chemical Information Data

SUPPLEMENTARY MATERIALS AND METHODS

Bacterial strains

The *M. tuberculosis* strains used in these studies included the laboratory strain H37Rv (ATCC 27294; American Type Culture Collection, Rockville, MD), which was obtained from the State Laboratory of Tuberculosis Reference of China.

MIC Determination[1]

The MIC was defined as the lowest concentration causing a 90% decrease of fluorescence when compared to the mean of compound free control samples. MICs against replicating *M. tuberculosis* were determined by the microplate Alamar blue assay (MABA). Bedaquiline was included as positive control. The compound stock solutions were 64 μg/ml. For the most active compounds, the tested range of concentrations was lowered to 0.015 μg/ml. *M. tuberculosis* H37Rv was grown to late log phase (70 to 100 Klett units) in Difco Middlebrook 7H9 Broth (catalog no. 271310) supplemented with 0.2% (vol/vol) glycerol, 0.05% Tween 80, and 10% (vol/vol) albumin-dextrose-catalase (BBL Middlebrook ADC Enrichment, catalog no. 212352) (7H9-ADC-TG). Cultures were centrifuged, washed twice, and resuspended in PBS (phosphate-buffered saline) buffer. Suspensions were then passed through an 8 μm-pore-size filter to remove clumps, and aliquots were frozen at -80°C. Twofold dilutions of riminophenazine analogs were prepared in 7H9-ADC-TG in a volume of 100 μl in 96-well, black, clear-bottom microplates (BD Biosciences, Franklin Lakes, NJ). *M. tuberculosis* (100 μl containing 2×105 CFU) was added, yielding a final testing volume of 200μl. The plates were incubated at 37°C; on day 7 of the incubation, 12.5 μl of 20% Tween 80 and 20 μl of Alamar blue were added to all wells. After an incubation at 37°C for 16 to 24 h the fluorescence was determined with an excitation wavelength of 530 nm and an emission wavelength of 590 nm.

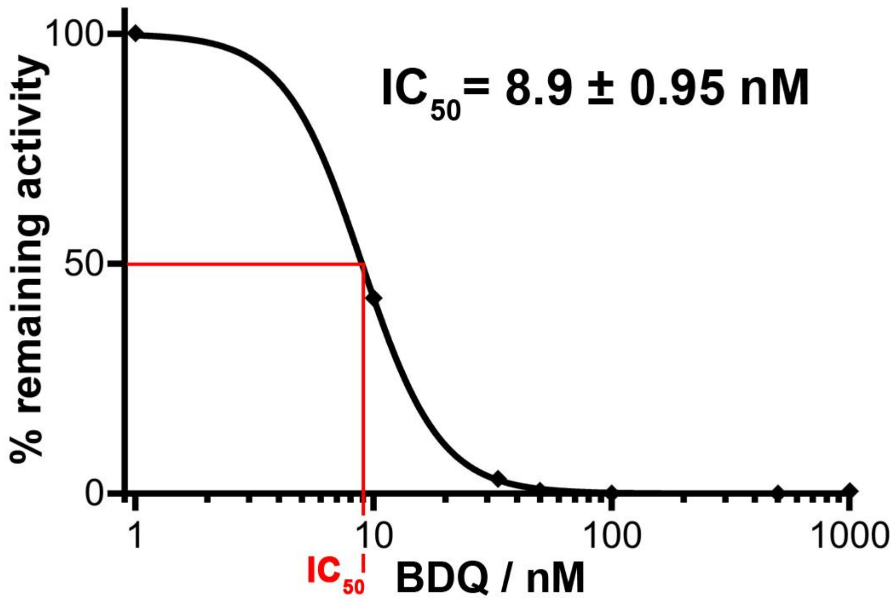

Figure S1. Concentration dependent inhibition of mycobacterial ATP synthesis by bedaquiline measured in *Mycobacterium phlei* inverted membrane vesicles. Bedaquiline was assayed for its ability to inhibit the ATP synthesis activity in inverted membrane vesicles from *M. phlei* and to determine its IC_{50}. Bedaquiline concentrations ranging from 0-1000 nM were tested using a luminescence based activity assay (see Methods). The graph illustrates the decrease of ATP synthesis activity as a function of increasing bedaquiline concentrations. The error for the IC_{50} value represents the standard error, as determined by Graphpad Prism.

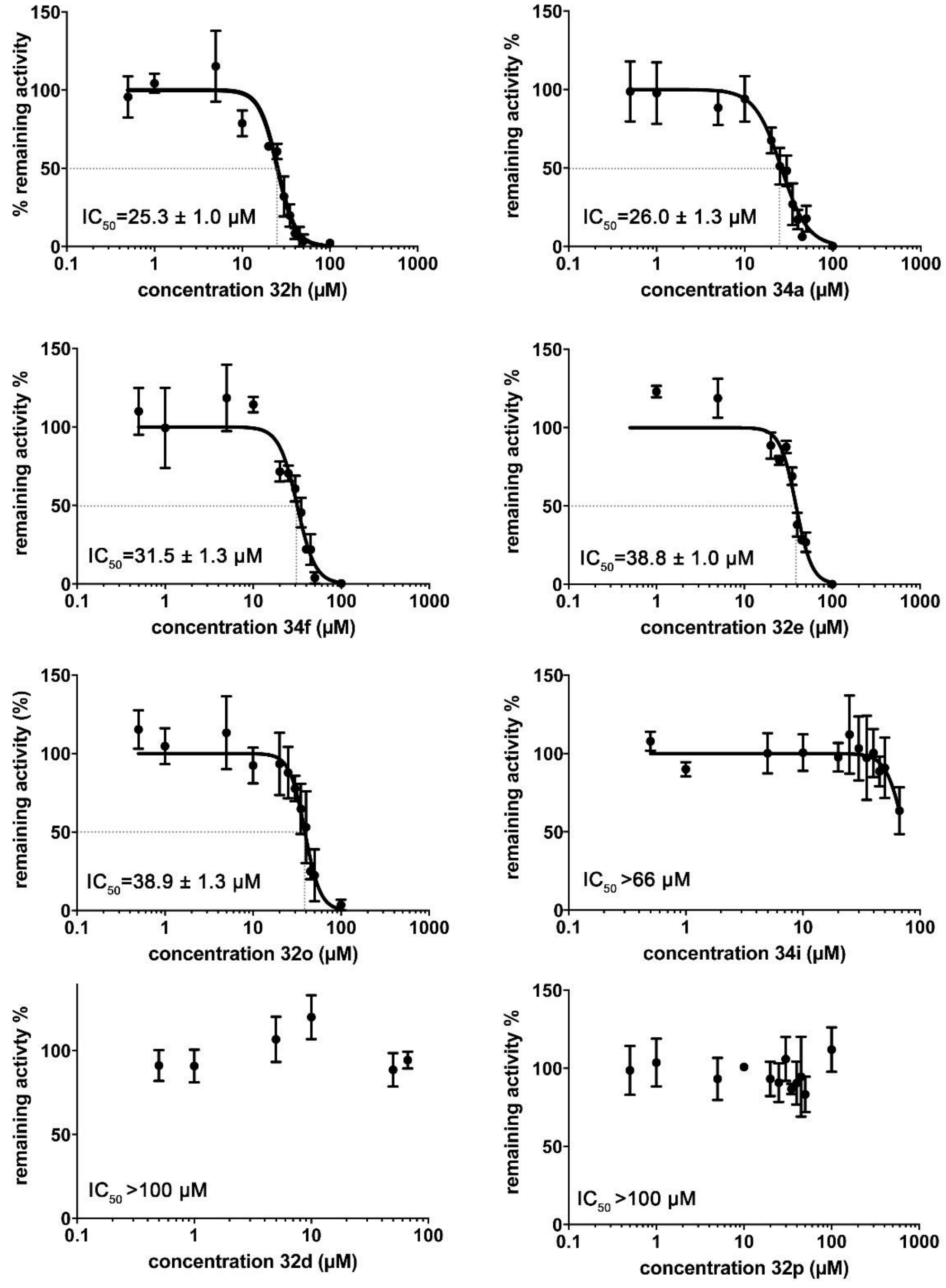

Figure S2. Concentration dependent inhibition of mycobacterial ATP synthesis by selected lead compounds measured in *Mycobacterium phlei* inverted membrane vesicles. Eight compounds, all showing antimycobacterial activity with MIC_{90} values in the sub- µg/ml range (Table S1), were assayed at a concentration range between 0.5 and 100 µM for their ability to inhibit the mycobacterial ATP synthesis activity using a luminescence based assay and *M. phlei* inverted membrane vesicles. The curves illustrate the decrease of ATP synthesis activity as a function of increasing inhibitor concentrations. Error bars represent the standard deviation of at least three biological replicates. The IC_{50} error values represent the standard errors, as determined by Graphpad Prism.

Table S1: Chemical and biological characteristics of the most potent lead compounds described in this work.

Compound	MIC (μg/ml)	IC_{50} (μM)[a]	CC_{50} (μg/ml)[b]	LogP[c]
32d	0.47	>100	12	7.98
32e	0.44	38.8	3.9	7.98
32h	0.62	25.3	4.6	7.64
32o	0.67	38.9	12	7.76
32p	0.51	>100	13	7.36
34a	0.62	26.0	4.0	7.05
34f	0.58	31.5	4.4	6.76
34i	0.55	>66	11	7.44
34m	0.43	20.3	3.0	5.55

[a] IC_{50} refers to the concentration, at which the ATP synthesis activity was reduced by 50%; [b]CC_{50} refers to the concentration, at which the growth of Vero cells was inhibited by 50%; [c]LogP, logarithm of the partition coefficient; values were calculated by using ChemBioDraw (Adept Scientific).

CHEMICAL SYNTHESES AND CHARACTERIZATION

General description

All anhydrous solvents and Grignard Reagent were purchased from J&K Chemical Ltd. All other reagents and solvents used in this study were commercially available and used without further purification. The ^{1}H NMR, ^{13}C NMR were recorded on Mercury-300, Mercury-400 or Bruker-AV600 spectrometer using TMS as an internal standard. The following multiplicity abbreviations are used: (s) singlet, (d) doublet, (t) triplet, (q) quartet, (m) multiplet, and (br) broad. ESI-HRMS data were measured on Thermo Exactive Orbitrap plus spectrometer. Melting points were determined on a Yanaco MP-J3 microscope melting point apparatus. Flash column chromatography was performed on a Biotage Isolera one. All final compounds have a purity of >95%, which was tested HPLC, LCMS and NMR spectroscopy.

First round of optimization

Series 1

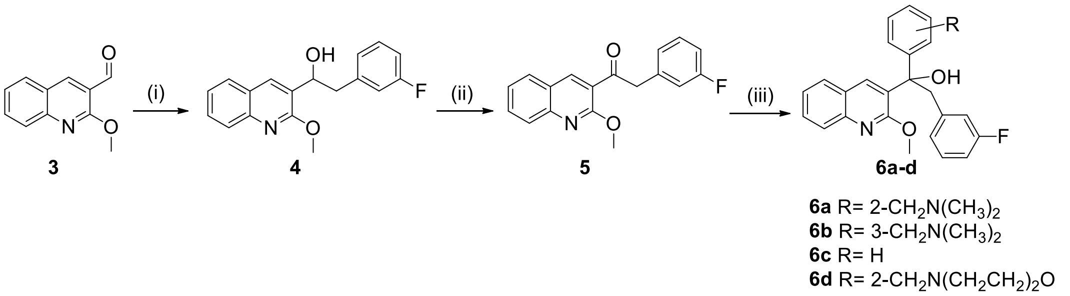

Scheme 1. Synthesis of compounds 6a-d. Reagents and conditions: (i) (3-fluorobenzyl)magnesium bromide, THF, RT, 2 h; (ii) DMP, CH_2Cl_2, RT, 3 h; (iii) phenyl magnesium bromide, THF, RT, 2 h.

2-(3-Fluorophenyl)-1-(2-methoxyquinolin-3-yl)ethanol (4)

The (3-fluorobenzyl)magnesium bromide (8.0 mmol) prepared in sodium-dried ether was added dropwise to the 2-methoxyquinoline-3-carbaldehyde **3** (1.1 g, 5.88 mmol) in anhydrous tetrahydrofuran at 0 °C under Ar, and then stirred at room temperature for 1 h. Then the reaction was quenched by saturated NH_4Cl (aq), extracted by dichloromethane (20 mL×3), dried over anhydrous Na_2SO_4, filtered and concentrated. The residue was purified by flash column chromatography with petroleum ether/ethyl acetate (3 : 1) to afford a white solid (1.15g, 66.5%). ^{1}H NMR (400 MHz, Acetone-d_6) δ 8.21 (s, 1 H), 7.82 (t, J = 9.2 Hz, 1 H), 7.62 (t, J = 7.6 Hz, 1 H), 7.41 (t, J = 7.6 Hz, 1 H), 7.26 (q, J = 7.2 Hz, 1 H), 7.09−7.02 (m, 2

H), 6.94 (t, J = 8.4 Hz, 1 H), 5.24 (q, J = 4.0 Hz, 1 H), 4.53 (d, J = 4.8 Hz, 1 H), 4.40 (s, 3 H), 3.22 (dd, J = 13.6, 3.2 Hz, 1 H), 2.91 (dd, J = 13.6, 4.0 Hz, 1 H). HRMS (ESI, m/z)$[M+H]^+$: 298.1150.

2-(3-Fluorophenyl)-1-(2-methoxyquinolin-3-yl)ethanone (5)

To a solution of 2-(3-fluorophenyl)-1-(2-methoxyquinolin-3-yl)ethanol **4** (1.00g, 3.36 mmol) in 15 mL of anhydrous dichloromethane, Dess-Martin periodinane (2.14g, 5.05 mmol) was added. The resulting mixture was stirred at room temperature for 1 h and then was quenched with saturated aqueous sodium thiosulfate (30 mL) and saturated aqueous sodium bicarbonate (30 mL). The organic layer was separated and the aqueous phase was extracted with dichloromethane (20 mL×2). The combined organic layers were dried over anhydrous Na_2SO_4, filtered and concentrated. The residue was purified by flash column chromatography with petroleum ether/ethyl acetate (20 : 1) to afford a yellow product (0.94g, 94.8%). ^1H NMR (300 MHz, acetone-d_6) δ 8.53 (s, 1 H), 7.99 (d, J = 8.1 Hz, 1 H), 7.85 (d, J = 7.8 Hz, 1 H), 7.77 (t, J = 7.8 Hz, 1 H), 7.49 (t, J = 7.5 Hz, 1 H), 7.35 (q, J = 7.5 Hz, 1 H), 7.16–7.08 (m, 2 H), 7.00 (t, J = 8.7 Hz, 1 H), 4.47 (s, 2 H), 4.18 (s, 3 H). HRMS (ESI, m/z)$[M+H]^+$: 296.1124.

General Procedure for the Synthesis of Compounds ***6a-d:***

The corresponding phenyllithium prepared by bromobenzene and n-butyllithium in sodium-dried ether was added dropwise to the 2-(3-fluorophenyl)-1-(2-methoxyquinolin-3-yl) ethanone in anhydrous tetrahydrofuran at 0 °C and stirred for 1 h at room temperature. Subsequently, the reaction was quenched by saturated NH_4Cl (aq), extracted by dichloromethane, dried over anhydrous Na_2SO_4, filtered and concentrated. The residue was purified by flash column chromatography to obtain the desired product.

1-(2-((Dimethylamino)methyl)phenyl)-2-(3-fluorophenyl)-1-(2-methoxyquinolin-3-yl)ethanol (6a)

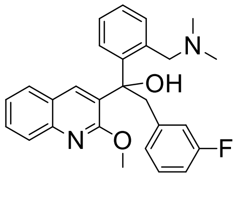

The residue was purified by silica gel column chromatography with dichloromethane/methanol (20: 1). White solid (78.8%). Mp 235−236ºC. ^{1}H NMR (400 MHz, Acetone-d_6) δ 9.12 (s, 1 H), 8.10 (s, 1 H), 7.88 (d, J = 8.0 Hz, 1 H), 7.73 (dd, J = 8.0, 4.4 Hz, 2 H), 7.58 (t, J = 7.6 Hz, 1 H), 7.44 (t, J = 8.0 Hz, 1 H), 7.35 (t, J = 7.6 Hz, 1 H), 7.23 (t, J = 7.6 Hz, 1 H), 7.11 (d, J = 6.4 Hz, 1 H), 6.97−6.90 (m, 1 H), 6.80 (d, J = 10.8 Hz, 1 H), 6.74 (t, J = 8.4 Hz, 1 H), 6.62 (d, J = 7.6 Hz, 1 H), 3.98 (d, J = 12.4 Hz, 1 H), 3.74 (s, 3 H), 3.60 (d, J = 12.0 Hz, 1 H), 3.10 (d, J = 12.4 Hz, 1 H), 2.59 (d, J = 12.0 Hz, 1 H), 2.07 (s, 6 H). ^{13}C NMR (150 MHz, Acetone-d_6) δ 163.56, 161.95, 159.94, 147.82, 146.71, 142.07, 142.02, 137.09, 136.11, 133.66, 131.30, 129.81, 129.16, 129.11, 128.53, 128.37, 128.29, 127.83, 127.82, 127.42, 127.33, 125.59, 124.98, 118.75, 118.61, 113.15, 113.01, 76.86, 63.47, 53.16, 45.40, 43.92. HRMS (ESI-TOF, m/z): calcd for $C_{27}H_{28}FN_2O_2$ $[M+H]^+$ 431.2129; found: 431.2109.

1-(3-((Dimethylamino)methyl)phenyl)-2-(3-fluorophenyl)-1-(2-methoxyquinolin-3-yl)ethanol (6b)

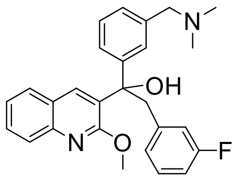

The residue was purified by silica gel column chromatography with dichloromethane/methanol (15: 1). White solid (80.7%). Mp 120−121ºC.^{1}H NMR (400 MHz, Acetone-d_6) δ 8.37 (s, 1 H), 7.81 (d, J = 8.0 Hz, 1 H), 7.75 (d, J = 8.3 Hz, 1 H), 7.60 (t, J = 7.6 Hz, 1 H), 7.44−7.36 (m, 2 H), 7.27 (d, J = 7.6 Hz, 1 H), 7.19 (t, J = 7.5 Hz, 1 H), 7.14 (d, J = 7.4 Hz, 1 H), 7.03 (q, J = 7.6 Hz, 1 H), 6.89 (d, J = 10.5 Hz, 1 H), 6.84 (d, J = 7.6 Hz, 1 H), 6.77 (t, J = 8.6 Hz, 1 H), 4.93 (s, 1 H), 4.13 (d, J = 13.2 Hz, 1 H), 3.90 (s, 3 H), 3.65 (d, J = 13.2 Hz, 1 H), 3.32 (s, 2 H), 2.11 (s, 6 H). ^{13}C NMR (100 MHz, Acetone-d_6) δ 164.11, 161.71, 159.96, 147.25, 146.46, 141.66, 141.58, 139.60, 136.32, 131.01, 129.94, 129.57, 129.49, 128.75, 128.21, 128.14, 127.60, 127.54, 127.51, 127.32, 126.11, 125.81, 125.00, 118.35, 118.14, 113.40, 113.19, , 77.19, 64.92, 53.38, 45.47, 45.17. HRMS (ESI-TOF, m/z): calcd for $C_{27}H_{28}FN_2O_2$ $[M+H]^+$ 431.2129; found: 431.2126.

2-(3-Fluorophenyl)-1-(2-methoxyquinolin-3-yl)-1-phenylethanol (6c)

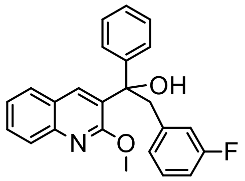

The residue was purified by silica gel column chromatography with petroleum ether /dichloromethane (2: 1). White solid (59.1%). Mp 107−108ºC.^{1}H NMR (500 MHz, Acetone-d_6) δ 8.36 (s, 1 H), 7.81 (d, J = 8.0 Hz, 1 H), 7.75 (d, J = 8.3 Hz, 1 H), 7.60 (td, J = 7.8, 1.0 Hz, 1 H), 7.47-7.40 (m, 2 H), 7.39 (t, J = 7.6 Hz, 1 H), 7.26 (t, J = 7.5 Hz, 2 H), 7.19 (t, J = 7.2 Hz, 1 H), 7.04 (q, J = 7.8 Hz, 1 H), 6.91 (d, J = 10.6 Hz, 1 H), 6.85 (d, J = 7.6 Hz, 1 H), 6.78 (t, J = 8.6 Hz, 1 H), 4.92 (s, 1 H), 4.15 (d, J = 13.3 Hz, 1 H), 3.90 (s, 3 H), 3.65 (d, J = 13.3 Hz, 1 H). ^{13}C NMR (125 MHz, Acetone-d_6) δ 163.87, 161.94, 159.90, 147.43, 146.45, 141.64, 141.57, 136.43, 130.84, 129.93, 129.58, 129.51, 128.72, 128.42, 127.53, 127.49, 127.47, 127.30, 127.08, 126.08, 124.99, 118.30, 118.13, 113.38, 113.22, , 77.20, 53.35, 45.04. HRMS (ESI-TOF, m/z): calcd for $C_{24}H_{21}FNO_2$ [M+H]$^+$ 374.1551; found: 374.1543.

2-(3-Fluorophenyl)-1-(2-methoxyquinolin-3-yl)-1-(3-(morpholinomethyl)phenyl)ethanol (6d)

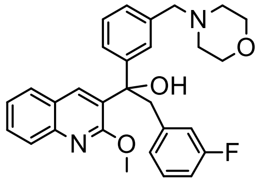

The residue was purified by silica gel column chromatography with dichloromethane/methanol (20: 1). White solid (86.9%). Mp 272−273ºC. ^{1}H NMR (400 MHz, $CDCl_3$) δ 8.67 (s, 1 H), 7.94 (s, 1 H), 7.78 (t, J = 7.3 Hz, 2 H), 7.59−7.51 (m, 2 H), 7.40 (t, J = 7.6 Hz, 1 H), 7.31 (t, J = 7.5 Hz, 1 H), 7.19 (t, J = 7.4 Hz, 1 H), 7.01 (d, J = 7.4 Hz, 1 H), 6.89 (q, J = 7.5 Hz, 1 H), 6.79 (d, J = 10.3 Hz, 1 H), 6.72 (t, J = 8.5 Hz, 1 H), 6.54 (d, J = 7.5 Hz, 1 H), 3.88 (d, J = 12.4 Hz, 1 H), 3.76−3.61 (m, 8 H), 3.07 (d, J = 12.5 Hz, 1 H), 2.61 (d, J = 12.5 Hz, 1 H), 2.30 (br s, 2 H), 2.19 (br s, 2 H). ^{13}C NMR (100 MHz, $CDCl_3$) δ 163.24, 160.82, 159.09, 146.82, 145.79, 140.26, 140.18, 136.02, 133.24, 133.06, 130.22, 129.09, 128.29, 128.21, 128.05, 127.29, 127.25, 126.72, 126.70, 126.62, 126.54, 124.59, 124.18, 118.22, 118.01, 112.79, 112.58, 77.21, 76.22, 66.47, 62.35, 52.94, 44.69. HRMS (ESI-TOF, m/z): calcd for $C_{29}H_{30}FN_2O_3$ [M+H]$^+$ 473.2235; found: 473.2235.

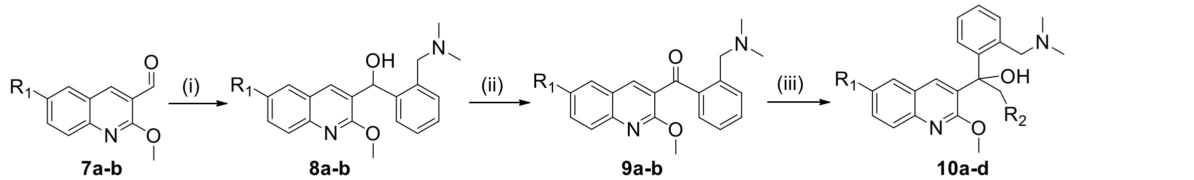

7a-b 8a-b 9a-b 10a-d

7a R_1= Br **7b** R_1= OCH_3

10a R_1= Br, R_2= 3-fluorophenyl
10b R_1= Br, R_2= benzyl
10c R_1= Br, R_2= 4-methoxyphenyl
10d R_1= OCH_3, R_2= 4-methoxyphenyl

Scheme 2. Synthesis of compounds 10a-d. Reagents and conditions: (i) (2-((dimethylamino)methyl)phenyl)magnesium bromide, THF, RT, 2 h; (ii) TBAP/NMO, 1, 4-Dioxane, RT, 15 h; (iii) appropriate Grignard reagent, THF, RT, 2 h.

(6-Bromo-2-methoxyquinolin-3-yl) (2-((dimethylamino)methyl)phenyl)methanol (8a)

To a solution of 6-bromo-2-methoxyquinoline-3-carbaldehyde **7a** (530 mg, 2.00 mmol) in anhydrate THF (5 mL) was added (3-((dimethylamino)methyl)phenyl)magnesium bromide (3.00 mmol in anhydrate THF 10 mL) at 0 °C and stirred for 1 h at room temperature. Subsequently, the reaction was quenched by saturated NH_4Cl (aq), extracted by dichloromethane, dried over anhydrous Na_2SO_4, filtered and concentrated. The residue was purified by flash column chromatography with dichloromethane/methanol (25:1) to afford **8a** as a white solid (670 mg, 83.8%). ^{1}H NMR (400 MHz, Acetone-d_6) δ 8.45 (s, 1 H), 8.17 (s, 1 H), 7.80–7.76 (m, 2 H), 7.31 (d, J = 7.3 Hz, 1 H), 7.23 (td, J = 7.4, 1.4 Hz, 1 H), 7.18 (td, J = 7.5, 1.5 Hz, 2 H), 6.82 (d, J = 7.6 Hz, 1 H), 6.17 (s, 1 H), 4.05 (d, J = 12.4 Hz, 1 H), 3.85 (s, 3 H), 3.26 (d, J = 12.4 Hz, 1 H), 2.29 (s, 6 H). HRMS (ESI, m/z): calcd for $C_{20}H_{22}BrN_2O_2$ $[M+H]^+$: 401.0859; found 401.0865.

(2, 6-Dimethoxyquinolin-3-yl) (2-((dimethylamino)methyl)phenyl)methanol (8b)

The procedure used to synthesize **8a** was repeated using 2, 6-dimethoxy-quinoline-3-carbaldehyde **7b** and (3-((dimethylamino)methyl)phenyl)magnesium to afford compound **8b** as a yellow solid in 72.1% yield. ^{1}H NMR (400 MHz, Acetone-d_6) δ 8.38 (s, 1 H), 7.73 (d, J = 9.0 Hz, 1 H), 7.45 (s, 1 H), 7.36 (s, 1 H), 7.29 (d, J = 7.7 Hz, 2 H), 7.19 (dt, J = 20.2, 7.1 Hz, 2 H), 6.82 (d, J = 7.3 Hz, 1 H), 6.18 (s, 1 H), 4.05 (d, J = 12.3 Hz, 1 H), 3.93 (s, 3 H), 3.81 (s, 3 H), 3.24 (d, J = 12.3 Hz, 1 H), 2.29 (s, 6 H). HRMS (ESI, m/z) : calcd for $C_{21}H_{25}N_2O_3$ $[M+H]^+$: 353.1860; found 353.1861.

(6-Bromo-2-methoxyquinolin-3-yl) (2-((dimethylamino)methyl)phenyl)methanone (9a)

To a solution of (6-bromo-2-methoxyquinolin-3-yl) (2-((dimethylamino)methyl)phenyl) methanol **8a** (100 mg, 0.25 mmol) in anhydrous dioxane (10 mL) was added tetrapropylammoniumperruthenate (9 mg, 0.03 mmol) and 4-Methylmorpholine N-oxide (59 mg, 0.50 mmol) and stirred at room temperature overnight. The mixture was then poured to water (10 mL), extracted with dichloromethane (10 mL×3). The combined organic layers were dried over anhydrous Na_2SO_4, filtered and concentrated. The residue was purified by flash column chromatography with dichloromethane/methanol (20:1) to afford a yellow oil (434 mg, 87.5%). ^{1}H NMR (400 MHz, Acetone-d_6) δ 8.26 (s, 1 H), 8.17 (d, J = 2.2 Hz, 1 H), 7.84 (dd, J = 8.9, 2.2 Hz, 1 H), 7.78 (d, J = 8.9 Hz, 1 H), 7.48 (td, J = 7.4, 1.9 Hz, 1 H), 7.46−7.36 (m, 3 H), 3.94 (s, 3 H), 3.39 (s, 2 H), 1.94 (s, 6 H). HRMS (ESI, m/z) : calcd for $C_{20}H_{20}BrN_2O_2$ [M+H]$^+$: 399.0703; found 399.0709.

(2, 6-Dimethoxyquinolin-3-yl) (2-((dimethylamino)methyl)phenyl)methanone (9b)

The procedure used to synthesize **9a** was repeated using (2, 6-dimethoxyquinolin-3-yl) (2-((dimethylamino)methyl)phenyl)methanol **8b**, tetrapropylammoniumperruthenate and 4-Methylmorpholine N-oxide to afford compound **9b** as a yellow solid in 77.2% yield. ^{1}H NMR (400 MHz, Acetone-d_6) δ 8.21 (s, 1 H), 7.75 (dt, J = 8.3, 1.0 Hz, 1 H), 7.51−7.43 (m, 1 H), 7.43−7.30(m, 5 H), 3.90 (s, 3 H), 3.89 (s, 3 H), 3.39 (s, 2 H), 1.95 (s, 6 H). HRMS (ESI, m/z) : calculated for $C_{21}H_{23}N_2O_3$ [M+H]$^+$: 351.1703; found 351.1767.

1-(6-Bromo-2-methoxyquinolin-3-yl)-1-(2-((dimethylamino)methyl)phenyl)-2-(3-fluorophenyl)ethanol (10a)

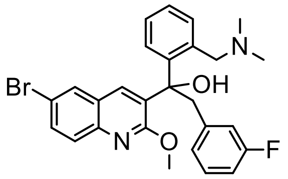

To a solution of (6-bromo-2-methoxyquinolin-3-yl) (2-((dimethylamino)methyl) phenyl)methanone **9a** (50 mg, 0.13 mmol) in anhydro ether (2 mL) was added (3-fluorobenzyl)magnesium bromide (2.0 mmol) and stirred for 2 h at room temperature. The reaction was then quenched by saturated NH_4Cl (10mL), extracted by dichloromethane (10 mL×3). The combined organic layers were dried over anhydrous Na_2SO_4, filtered and concentrated. The residue was purified by flash column chromatography with

dichloromethane/methanol (20:1) and followed by crystallization in ether to afford a white solid (36 mg, 56.2%). Mp 220−221°C. ^{1}H NMR (600 MHz, Acetone-d_6) δ 9.23 (s, 1 H), 8.09 (s, 1 H), 7.98 (d, J = 1.4 Hz, 1 H), 7.88 (d, J = 8.0 Hz, 1 H), 7.72−7.64 (m, 2 H), 7.44 (td, J = 7.8, 1.4 Hz, 1 H), 7.25 (td, J = 7.4, 1.1 Hz, 1 H), 7.12 (dd, J = 7.5, 1.3 Hz, 1 H), 6.95 (td, J = 7.9, 6.3 Hz, 1 H), 6.82−6.78 (m, 1 H), 6.78−6.72 (m, 1 H), 6.60 (d, J = 7.6 Hz, 1 H), 3.96 (d, J = 12.4 Hz, 1 H), 3.75 (s, 3 H), 3.59 (d, J = 12.3 Hz, 1 H), 3.11 (d, J = 12.3 Hz, 1 H), 2.62 (d, J = 12.4 Hz, 1 H), 2.09 (s, 6 H). ^{13}C NMR (125 MHz, Acetone-d_6) δ 163.57, 161.97, 160.39, 147.45, 145.43, 141.88, 141.83, 136.25, 136.13, 133.68, 132.90, 132.74, 130.37, 129.38, 129.23, 129.18, 128.59, 128.34, 127.76, 127.55, 127.05, 118.74, 118.60, 117.59, 113.25, 113.12, 76.90, 63.44, 53.38, 45.36, 43.95.

HRMS (ESI-TOF, m/z): calcd for $C_{27}H_{27}BrFN_2O_2$ $[M+H]^+$ 509.1235; found: 509.1238.

1-(6-Bromo-2-methoxyquinolin-3-yl)-1-(2-((dimethylamino)methyl)phenyl)-3-phenylpropan-1-ol (10b)

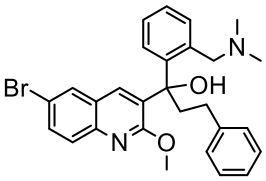

The procedure used to synthesize **10a** was repeated using **9a** and phenethylmagnesium bromide to afford **10b** as a white solid in 38.1% yield. Mp 195−196 ºC. ^{1}H NMR (400 MHz, Acetone-d_6) δ 9.23 (s, 1 H), 8.76 (s, 1 H), 8.28 (d, J = 2.0 Hz, 1 H), 7.79 (d, J = 8.0 Hz, 1 H), 7.74 (dd, J = 8.9, 2.1 Hz, 1 H), 7.70 (d, J = 8.8 Hz, 1 H), 7.39 (t, J = 7.1 Hz, 1 H), 7.21 (t, J = 7.4 Hz, 3 H), 7.18−7.06 (m, 4 H), 3.65 (s, 3 H), 3.26 (d, J = 12.3 Hz, 1 H), 2.93−2.83 (m, 2 H), 2.70 (d, J = 12.3 Hz, 1 H), 2.59 (td, J = 12.6, 11.7, 3.3 Hz, 1 H), 2.22 (td, J = 12.2, 11.3, 3.7 Hz, 1 H), 2.16 (s, 6 H). ^{13}C NMR (125 MHz, Acetone-d_6) δ 160.54, 147.29, 145.61, 144.07, 136.13, 136.08, 133.65, 133.56, 132.96, 130.64, 129.44, 129.24, 129.10, 128.86, 128.41, 127.46, 127.32, 126.30, 117.71, 76.50, 63.44, 53.23, 44.02, 41.97, 31, 18. HRMS (ESI-TOF, m/z): calcd for $C_{28}H_{30}BrN_2O_2$ $[M+H]^+$ 505.1485; found: 505.1483.

1-(6-Bromo-2-methoxyquinolin-3-yl)-1-(2-((dimethylamino)methyl)phenyl)-2-(4-methoxyphenyl)ethanol (10c)

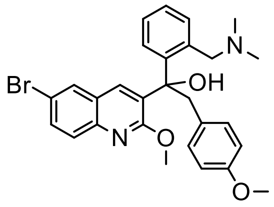

The procedure used to synthesize **10a** was repeated using **9a** and (4-methoxybenzyl) magnesium bromide to afford **10c** as a white solid in 40.0% yield. Mp 232−233 ºC. ^{1}H NMR (400 MHz, Acetone-*d*$_6$) δ 9.03 (s, 1 H), 8.06 (s, 1 H), 7.96 (s, 1 H), 7.87 (d, *J* = 7.8 Hz, 1 H), 7.68 (s, 2 H), 7.42 (t, *J* = 7.3 Hz, 1 H), 7.23 (t, *J* = 7.3 Hz, 1 H), 7.10 (d, *J* = 7.3 Hz, 1 H), 6.78 (d, *J* = 8.5 Hz, 2 H), 6.54 (d, *J* = 8.6 Hz, 2 H), 3.86 (d, *J* = 12.5 Hz, 1 H), 3.74 (s, 3 H), 3.62 (s, 3 H), 3.50 (d, *J* = 12.5 Hz, 1 H), 3.08 (d, *J* = 12.3 Hz, 1 H), 2.60 (d, *J* = 12.4 Hz, 1 H), 2.08 (s, 6 H). ^{13}C NMR (100 MHz, Acetone-*d*$_6$) δ 160.53, 158.87, 147.85, 145.37, 136.27, 136.22, 133.59, 133.27, 132.87, 132.75, 130.63, 130.35, 129.38, 128.49, 128.39, 127.37, 127.14, 117.48, 113.20, 77.02, 63.51, 55.23, 53.33, 44.91, 43.98. HRMS (ESI-TOF, m/z): calcd for $C_{28}H_{30}{}^{81}BrN_2O_3$ $[M+H]^+$ 523.1414; found: 523.1402.

1-(2, 6-Dimethoxyquinolin-3-yl)-1-(2-((dimethylamino)methyl)phenyl)-2-(4-methoxyphenyl) ethanol (10d)

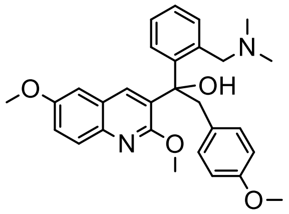

The procedure used to synthesize **10a** was repeated using **9b** and (4-methoxybenzyl) magnesium bromide to afford **10d** as a white solid in 41.7% yield. Mp 215−216 ºC. ^{1}H NMR (400 MHz, Acetone-*d*$_6$) δ 8.91 (s, 1 H), 8.02 (s, 1 H), 7.86 (d, *J* = 8.0 Hz, 1 H), 7.65 (d, *J* = 9.0 Hz, 1 H), 7.41 (t, *J* = 7.6 Hz, 1 H), 7.25−7.18 (m, 2 H), 7.16 (d, *J* = 2.3 Hz, 1 H), 7.08 (d, *J* = 7.4 Hz, 1 H), 6.80 (d, *J* = 8.3 Hz, 2 H), 6.55 (d, *J* = 8.4 Hz, 2 H), 3.88 (d, *J* = 12.4 Hz, 1 H), 3.85 (s, 3 H), 3.69 (s, 3 H), 3.63 (s, 3 H), 3.49 (d, *J* = 12.4 Hz, 1 H), 3.08 (d, *J* = 12.2 Hz, 1 H), 2.57 (d, *J* = 12.3 Hz, 1 H), 2.07 (s, 6 H). ^{13}C NMR (100 MHz, Acetone-*d*$_6$) δ 158.80, 158.67, 157.21, 148.30, 141.96, 136.37, 136.23, 133.56, 132.93, 131.87, 130.89, 128.60, 128.40, 128.38, 127.20, 126.34, 121.14, 113.17, 107.15, 76.98, 63.52, 55.81, 55.22, 52.94, 44.92, 43.99. HRMS (ESI-TOF, m/z): calcd for $C_{29}H_{33}N_2O_4$ $[M+H]^+$ 473.2435; found: 473.2437.

Series 2

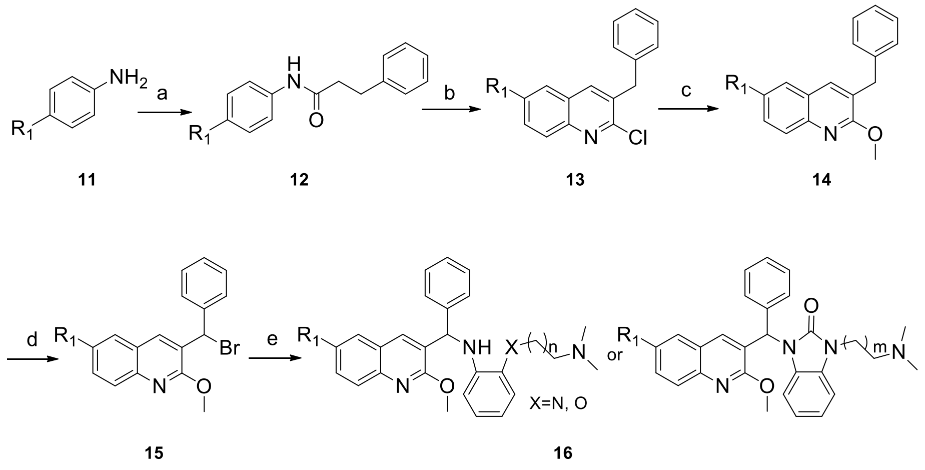

Scheme 3. Synthesis scheme of *N*-linked compounds 17. Reagents and conditions: (a) acylchloride, Et_3N, CH_2Cl_2, RT, 5 h; (b) $POCl_3$, DMF, 100°C, 16 h; (c) NaOMe/MeOH, reflux, 6 h; (d) NBS/CCl_4, reflux, 3 h; (e) 1: substituted aniline, K_2CO_3, DMF, RT, 2h; or 2: multistep (see the following information).

*General Procedure for the Synthesis of Compounds **12a-b**:*

Pyridine (1.5eq) was added to a solution of aniline in the solvent of anhydrous dichloromethane, and the mixture was cooled in the ice bath. Then hydrocinnamoyl chloride (1.0 eq) was added dropwise. After stirring at room temperature for 2 h, the reaction was quenched by water. The organic layers were washed with 2 mol·L^{-1} hydrochloric acid (50 mL×2), followed with saturated sodium bicarbonate solution (50 mL×2) and dried over anhydrous Na_2SO_4, then filtered and concentrated. The residue was recrystallized from petroleum ether and ethyl acetate to afford a white solid.

N, 3-Diphenylpropanamide (12a). White solid (93.6%). ^{1}H NMR (300 MHz, Acetone-d_6) δ 9.08 (br s, 1 H), 7.64 (d, J = 7.8 Hz, 2 H), 7.31−7.18 (m, 6 H), 7.18−7.12 (m, 1 H), 7.03 (d, J = 7.5 Hz, 1 H), 3.00 (d, J = 7.5 Hz, 2 H), 2.67 (d, J = 7.5 Hz, 2 H). HRMS (ESI, m/z)[M+H]$^+$: 226.1230.

N-(4-Bromophenyl)-3-phenylpropanamide (12b). White solid (98.2%). ^{1}H NMR (300 MHz, Acetone-d_6) δ 9.22 (br s, 1 H), 7.62 (d, J = 6.3 Hz, 2 H), 7.44 (d, J = 6.6 Hz, 2 H), 7.26 (s, 4 H), 7.18 (br s, 1 H), 2.99 (t, J = 6.0 Hz, 2 H), 2.68 (t, J = 5.7 Hz, 2 H). HRMS (ESI, m/z)[M+H]$^+$: 304.0343.

General Procedure for the Synthesis of Compounds ***13a-b****:*

Phosphorus oxychloride was added drop wise to ice cooled N, N-dimethylformamide (0.3 mol, 2.3 ml). After 30 minutes of stirring, N, 3-diphenylpropanamide was added. The mixture was then stirred at 80 °C for 18 h and poured into ice-water. The solution was extracted by dichloromethane (10 mL×3) and the combined organic layers washed by saturated sodium bicarbonate, dried over anhydrous Na_2SO_4, filtered and concentrated. The residue was purified by flash column chromatography with petroleum ether/ethyl acetate (20: 1) to afford a white solid.

3-Benzyl-2-chloroquinoline (13a). White solid (82.2%). ^1H NMR (300 MHz, Acetone-d_6) δ 8.19 (s, 1 H), 7.94 (d, J = 8.4 Hz, 2 H), 7.79 (d, J = 7.5 Hz, 1 H), 7.62 (t, J = 7.2 Hz, 1 H), 7.40−7.30 (m, 4 H), 7.27 (t, J = 6.6 Hz, 1 H), 4.29 (s, 2 H). HRMS (ESI, m/z)$[M+H]^+$: 254.0719.

3-Benzyl-6-bromo-2-chloroquinoline (13b). White solid (46.0%). ^1H NMR (300 MHz, Acetone-d_6) δ 8.17 (s, 2 H), 7.81 (s, 2 H), 7.38−7.28 (m, 5 H), 4.28 (s, 2 H). HRMS (ESI, m/z)$[M+H]^+$: 331.9840.

General Procedure for the Synthesis of Compounds ***14a-b****:*

To a solution of 2-chloroquinoline in methanol sodium methoxide-methanol solution was added (1.1eq) and heated to reflux at 90 °C for 6 h. The mixture was concentrated and poured to water, then extracted with dichloromethane (10 mL×3). The combined organic layers were dried over anhydrous Na_2SO_4, filtered and concentrated. The residue was purified by flash column chromatography with petroleum ether/ethyl acetate (20: 1) to afford a white solid.

3-Benzyl-2-methoxyquinoline (14a). White solid (99.3%). ^1H NMR (300 MHz, Acetone-d_6) δ 7.89 (s, 1 H), 7.82−7.74 (m, 2 H), 7.61 (t, J = 7.8 Hz, 1 H), 7.34 (t, J = 7.2 Hz, 1 H), 7.33−7.27 (m, 3 H), 7.23 (d, J = 3.6 Hz, 1 H), 4.06 (5H, s). HRMS (ESI, m/z)$[M+H]^+$: 250.1244.

3-Benzyl-6-bromo-2-methoxyquinoline (14b). White solid (98.2%).^1H NMR (300 MHz, Acetone-d_6) δ 7.97 (s, 1 H), 7.87 (s, 1 H), 7.71−7.65 (m, 2 H), 7.35−7.27 (m, 4 H), 7.26−7.20 (m, 1 H), 4.06 (s, 2 H), 4.05 (s, 3 H). HRMS (ESI, m/z)$[M+H]^+$: 328.0348.

1-(2-(Dimethylamino)ethyl)-3-((2-methoxyquinolin-3-yl) (phenyl)methyl)-1H-benzo[d]imidazol-2 (3 H)-one (16a)

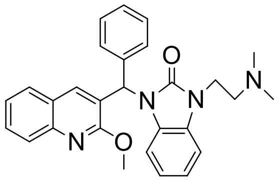

To a solution of 3-benzyl-2-methoxyquinoline (**14a**) (4.00 g, 16.05 mmol) in carbon tetrachloride (30 mL) NBS (0.19g, 0.8 mmol) and benzoyl peroxide were added. After heating to reflux at 60 °C for 2 h, the mixture were filtered and concentrated. Then the residue was added to a mixture of anhydrous potassium carbonate (6.65 g, 48.15 mmol), o-phenylenediamine (3.47 g, 32.1 mmol) in DMF and stirred at 50 °C for 3 h. Water (300 mL) was added to quench the reaction. The mixture was extracted by dichloromethane (10 mL×3) and the combined organic layers were concentrated. The residue was purified by flash column chromatography with petroleum ether/ethyl acetate (3: 1) to afford N1-((2-methoxyquinolin-3-yl) (phenyl)methyl)benzene-1, 2-diamine as a white solid (3.95 g, 69.3%). ^{1}H NMR (300 MHz, Acetone-d_6) δ 8.27 (s, 1 H), 7.81−7.75 (m, 2 H), 7.62 (d, J = 7.5 Hz, 1 H), 7.50 (d, J = 7.5 Hz, 2 H), 7.40−7.33 (m, 3 H), 7.25 (t, J = 6.3 Hz, 1 H), 6.72 (d, J = 6.6 Hz, 1 H), 6.50−6.43 (m, 2 H), 6.39 (d, J = 7.2 Hz, 1 H), 5.95 (d, J = 5.2 Hz, 1 H), 4.57 (br s, 1 H), 4.28 (br s, 2 H), 4.04 (s, 3 H). HRMS (ESI, m/z)$[M+H]^+$: 356.2112.

To a solution of N1-((2-methoxyquinolin-3-yl) (phenyl)methyl)benzene-1, 2-diamine (547 mg, 1.54mmol) in dichloromethane (10 mL) triethylamine (311 mg, 3.08 mmol) was added and the mixture was cooled in an ice bath. Then triphosgene (160 mg, 0.54 mmol) was added dropwise. After stirring for 2h, the reaction was quenched by H_2O (20mL). The organic layers were washed with 2 mol·L^{-1} hydrochloric acid (20 mL) and saturated sodium bicarbonate solution (30 mL) subsequently and dried over anhydrous Na_2SO_4, then filtered and concentrated. The residue was recrystallized from petroleum ether and ethyl acetate to afford 1-((2-methoxyquinolin-3-yl) (phenyl)methyl)-1H-benzo[d]imidazol-2(3H)-one as a white solid (430 mg, 73.3%). ^{1}H NMR (300 MHz, DMSO-d_6) δ 11.07 (s, 1 H), 7.82−7.74 (m, 3 H), 7.67 (d, J = 7.5 Hz, 1 H), 7.45−7.35 (m, 4 H), 7.26 (d, J = 6.6 Hz, 2 H), 7.06−6.96 (m, 2 H), 6.93 (d, J = 7.5 Hz, 1 H), 6.75 (d, J = 7.5 Hz, 1 H), 6.52 (d, J = 8.4 Hz, 1 H), 3.88 (s, 3 H). HRMS (ESI, m/z)$[M+H]^+$: 382.1615.

2-bromo-N, N-dimethylethanamine (118 mg, 0.79 mmol) was added to a mixture of 1-((2-methoxyquinolin-3-yl)(phenyl)methyl)-1H-benzo[d]imidazol-2(3H)-one (200 mg, 0.53 mmol) and NaH (14 mg, 0.57 mmol) in DMF and stirred at room temperature for 30 min. Then water was added to quench the reaction. The mixture was extracted by dichloromethane (10 mL×3) and the combined organic layers were concentrated and purified by flash column chromatography with dichloromethane/methanol (25: 1) to afford **16a** as a white solid (44.2%). Mp 132−133 °C. ^{1}H NMR (400 MHz, Acetone-d_6) δ 7.87 (s, 1 H), 7.81 (d, J = 8.4 Hz, 1 H), 7.14 (t, J = 8.0 Hz, 1 H), 7.66 (t, J = 7.6 Hz, 1 H), 7.44−7.35(m, 4 H), 7.33 (d, J = 7.2 Hz, 2 H), 7.19 (d, J = 8.0 Hz, 1 H), 7.14 (s, 1 H), 7.00 (t, J = 8.0 Hz, 1 H), 7.79 (t, J = 8.0 Hz, 1 H), 6.66 (t, J = 8.0 Hz, 1 H), 4.06−3.98 (m, 2 H), 3.92 (s, 3 H), 2.68−2.62 (m, 2 H), 2.25 (s, 6 H). ^{13}C NMR (100 MHz, Acetone-d_6) δ 160.86, 154.75, 146.85, 138.78, 138.74, 130.69, 130.48, 130.13, 129.40, 128.85, 128.61, 128.55, 127.49, 125.64, 125.06, 124.36, 121.63, 121.30, 110.17, 108.76, 57.55, 56.00, 53.87, 45.75, 39.87. HRMS (ESI-TOF, m/z): calcd for $C_{28}H_{29}N_4O_2$ [M+H]$^+$ 453.2496; found: 453.2498.

1-(3-(Dimethylamino)propyl)-3-((2-methoxyquinolin-3-yl) (phenyl)methyl)-1H-benzo[d]imidazol-2(3H)-one (16b)

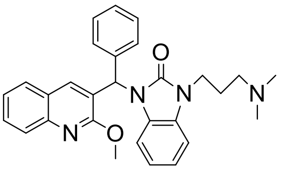

The procedure used to synthesize **16a** was repeated using **1-((2-methoxyquinolin-3-yl) (phenyl)methyl)-1H-benzo[d]imidazol-2 (3 H)-one** and 3-bromo-N, N-dimethylpropan-1-amine as starting material to afford **16b** as a white solid (37.6%). Mp 120−121 °C. ^{1}H NMR (300 MHz, Acetone-d_6) δ 7.87 (s, 1 H), 7.81 (d, J = 8.4 Hz, 1 H), 7.72 (d, J = 8.1 Hz, 1 H), 7.66 (d, J = 7.8 Hz, 1 H), 7.45−7.37 (m, 4 H), 7.35 (m, 2 H), 7.23 (d, J = 7.8 Hz, 1 H), 7.15 (s, 1 H), 7.01 (d, J = 7.5 Hz, 1 H), 6.79 (d, J = 7.5 Hz, 1 H), 6.66 (d, J = 7.8 Hz, 1 H), 4.00 (t, J = 6.6 Hz, 2 H), 3.92 (s, 3 H), 2.34 (t, J = 6.6 Hz, 2 H), 2.18 (s, 6 H), 1.96−1.90 (m, 2 H). ^{13}C NMR (100 MHz, Acetone-d_6) δ 160.85, 154.67, 146.86, 138.79, 130.76, 130.51, 130.06, 129.44, 128.85, 128.63, 128.59, 127.50, 125.64, 125.09, 124.37, 121.67, 121.31, 110.21, 108.66, 57.00, 56.01, 53.91, 45.36, 39.57, 26.88. HRMS (ESI-TOF, m/z): calcd for $C_{29}H_{31}N_2O_4$ [M+H]$^+$ 467.2442; found: 467.2445.

General Procedure for the Synthesis of Compounds ***16c-e****:*

To a solution of **14a-b** in carbon tetrachloride (6 mL) NBS (0.19g, 0.80 mmol) and benzoyl peroxide was added. After heated to reflux at 60 °C for 2 h, the mixture was filtered and concentrated. Then the residue was added to a mixture of anhydrous potassium carbonate (6.65 g, 48.15 mmol), substituted aniline (32.1 mmol) in DMF and stirred at 50 °C for 3 h. Water (300 mL) was added to quench the reaction. The mixture was extracted by dichloromethane (10 mL×3) and the combined organic layers were concentrated. Then the residue was purified by flash column chromatography to afford the desired product.

N1-(2-(Dimethylamino)ethyl)-N2-((2-methoxyquinolin-3-yl) (phenyl)methyl)benzene -1, 2-diamine(16c).

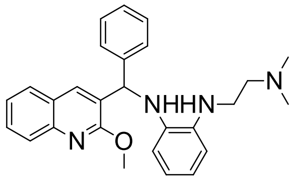

The residue was purified by flash column chromatography with dichloromethane/methanol (25:1). Yellow solid (52.8%). Mp 123-124 ºC. ^{1}H NMR (400 MHz, Acetone-d_6) δ 8.27 (s, 1 H), 7.81−7.75 (m, 2 H), 7.62 (t, J = 7.8 Hz, 1 H), 7.50 (d, J = 7.5 Hz, 2 H), 7.39 (t, J = 7.2 Hz, 1 H), 7.33 (d, J = 7.5 Hz, 2 H), 7.25 (t, J = 7.2 Hz, 1 H), 6.70−6.64 (m, 2 H), 6.52−6.45 (m, 2 H), 5.92 (d, J = 4.8 Hz, 1 H), 4.72 (d, J = 3.6 Hz, 1 H), 4.05 (s, 3 H), 3.21−3.15 (m, 2 H), 2.56−2.50 (m, 2 H), 2.18 (s, 6 H). ^{13}C NMR (100 MHz, Acetone-d_6) δ 160.92, 146.58, 143.16, 139.05, 136.78, 136.56, 129.97, 129.25, 128.55, 128.45, 128.19, 128.07, 127.57, 126.37, 125.00, 120.23, 119.09, 114.14, 112.85, 59.04, 57.96, 53.83, 45.54, 42.88. HRMS (ESI-TOF, m/z): calcd for $C_{27}H_{31}N_4O$ $[M+H]^+$ 427.2492; found: 427.2490.

N-((6-Bromo-2-methoxyquinolin-3-yl) (phenyl)methyl)-2-(2-(dimethylamino)ethoxy) aniline (16d).

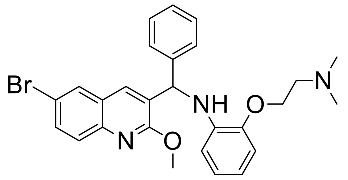

The residue was purified by flash column chromatography with dichloromethane/methanol (25:1). Yellow solid (54.3%). Mp 68-69 ºC. ^{1}H NMR (300 MHz, Acetone-d_6) δ 8.25 (s, 1 H), 8.04 (s, 1 H), 7.75−7.69 (m, 2 H), 7.46 (d, J = 7.2 Hz, 2 H), 7.35 (t, J = 6.9 Hz, 2 H), 7.27 (t,

J = 7.2 Hz, 1 H), 6.90 (d, *J* = 8.1 Hz, 1 H), 6.69 (d, *J* = 7.2 Hz, 1 H), 6.57 (d, *J* = 7.5 Hz, 1 H), 6.42 (d, *J* = 7.8 Hz, 1 H), 5.89 (d, *J* = 5.1 Hz, 1 H), 5.68 (d, *J* = 5.1 Hz, 1 H), 4.15−4.08 (m, 2 H), 4.03 (s, 3 H), 2.60 (t, *J* = 5.1 Hz, 2 H), 2.11 (s, 6 H). ^{13}C NMR (100 MHz, Acetone-d_6) δ 161.29, 147.32, 145.26, 142.59, 139.14, 135.38, 132.96, 130.50, 129.50, 129.30, 129.13, 128.44, 128.20, 127.67, 122.71, 117.75, 117.58, 114.38, 112.07, 68.89, 59.12, 57.85, 53.98, 45.80. HRMS (ESI-TOF, m/z): calcd for $C_{27}H_{29}BrN_3O_2$ $[M+H]^+$ 506.1438; found: 506.1454.

1-((6-Bromo-2-methoxyquinolin-3-yl) (phenyl)methyl)-4-methyl-1, 2, 3, 4-tetrahydro-quinoxaline (16e)

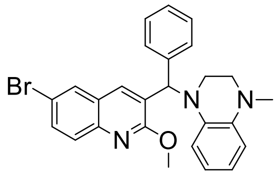

The residue was purified by flash column chromatography with petroleum ether/ethyl acetate (10:1). White solid (52.7%). Mp 144-145 ºC. ^{1}H NMR (400 MHz, Acetone-d_6) δ 8.07 (s, 1 H), 7.99 (s, 1 H), 7.79−7.72 (m, 2 H), 7.40−7.34 (m, 2 H), 7.34 (t, *J* = 6.4 Hz, 1 H), 7.23 (d, *J* = 7.2 Hz, 2 H), 6.57−6.51 (m, 2 H), 6.46 (td, *J* = 7.2, 2.0 Hz, 1 H), 6.37 (d, *J* = 8.0 Hz, 1 H), 6.29 (s, 1 H), 3.95 (s, 3 H), 3.41−3.35 (m, 1 H), 3.21 (m, 2 H), 3.16− 3.10(m, 1 H), 2.84 (s, 3 H). ^{13}C NMR (100 MHz, Acetone-d_6) δ 161.80, 145.54, 140.10, 137.91, 137.20, 136.26, 133.20, 130.77, 129.85, 129.62, 129.42, 128.42, 127.56, 127.41, 119.22, 118.27, 117.65, 112.04, 111.63, 61.90, 54.15, 50.49, 45.52, 39.64. HRMS (ESI-TOF, m/z): calcd for $C_{26}H_{25}BrN_3O$ $[M+H]^+$ 474.1176; found: 474.1163.

1-(1-((6-Bromo-2-methoxyquinolin-3-yl) (phenyl)methyl)-1H-indol-3-yl)-N, N-dimethylmethanamine (16f)

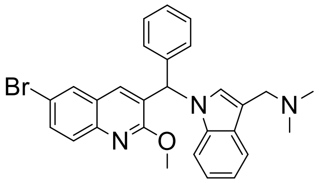

To a solution of **14b** (651 mg, 1.46 mmol) in carbon tetrachloride (5 mL) NBS (274 mg, 1.54 mmol) and benzoyl peroxide (8 mg, 0.04 mmol) was added. After heated to reflux at 60 °C for 2 h, the mixture was filtered and concentrated. Then the residue was added to a mixture of anhydrous caesium carbonate (573 mg, 1.76 mmol), 1-(indolin-3-yl)-N, N-

dimethylmethanamine (310 mg, 1.76 mmol) in DMF. After stirring at 50 °C for 3 h, the mixture was filtered and concentrated. The residue was purified by flash column chromatography with petroleum ether/ethyl acetate/triethylamine (40: 5: 1) to afford 1-(1-((6-bromo-2-methoxyquinolin-3-yl)(phenyl)methyl)indolin-3-yl)-N, N-dimethylmethanamine (382 mg, 52.2%) (m/z[M+H]$^+$: 502.1521), which was used directly for the next reaction.

To a solution of the compound described above (200 mg, 0.40 mmol) in tetrahydrofuran (6 mL) was added the solution of DDQ (110 mg, 0.48 mmol) in tetrahydrofuran (4 mL) under argon and stirred at room temperature over night. Then saturated $NaHCO_3$ solution (10 mL) was added to quench the reaction. The mixture was extracted by ethyl acetate (10 mL×3) and the combined organic layers were concentrated. The residue was purified by flash column chromatography with petroleum ether/ethyl acetate/triethylamine (40: 4 : 1) to give **16f** as a yellow solid (188 mg 94.16%. Mp 173-174 ºC. ^{1}H NMR (300 MHz, Acetone-d_6) δ 7.90 (s, 1 H), 8.02 (s, 1 H), 7.80−7.74 (m, 2 H), 7.73 (dd, J = 6.0, 0.6 Hz, 1 H), 7.54 (s, 1 H), 7.43−7.35 (m, 3 H), 7.32 (d, J = 7.5 Hz, 1 H), 7.29−7.23 (m, 3 H), 7.10−7.04 (m, 1 H), 6.90 (s, 1 H), 3.97 (s, 3 H), 3.53 (s, 2 H), 2.16 (s, 6 H). 13C NMR (100 MHz, Acetone-d_6) δ 161.16, 145.74, 139.58, 137.95, 137.38, 133.68, 130.80, 129.79, 129.67, 129.20, 129.03, 127.26, 127.07, 126.56, 122.56, 120.84, 120.18, 117.85, 113.91, 110.88, 58.67, 55.57, 54.33, 45.49. HRMS (ESI-TOF, m/z): calcd for $C_{28}H_{27}BrN_3O$ [M+H]$^+$ 500.1332; found: 500.1381.

Series 3

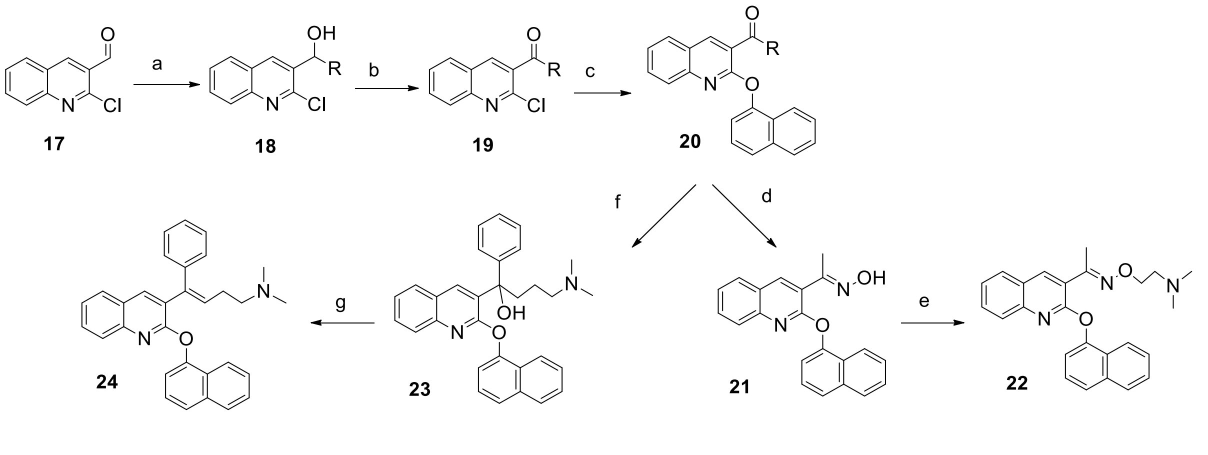

Scheme 4. Synthesis of compounds 22-23 of series 3. Reagents and conditions: (a) CH_3MgI or phenylmagnesium bromide, THF, 0°C; (b) Dess-Martin periodinane, CH_2Cl_2, RT; (c)1-naphthol, K_2CO_3, DMSO, 100°C; (d) Hydroxylamine hydrochloride, Et_3N, EtOH, RT; (e) 2-Dimethylaminoethyl chloride hydrochloride, NaH, THF; (f) (3-(dimethylamino)propyl)magnesium chloride; (g) HCl/EtOH, reflux.

*General Procedure for the Synthesis of Compounds **18a-b**:*

The corresponding Grignard reagent (1.8 eq) prepared in sodium-dried ether was added dropwise to the 2-chloroquinoline-3-carbaldehyde **19** (1 eq) in anhydrous tetrahydrofuran at 0 °C, and then stirred at room temperature for 1 h. Then the reaction was quenched by saturated NH_4Cl solution, extracted by dichloromethane, dried over anhydrous Na_2SO_4, filtered and concentrated. The residue was purified by flash column chromatography with petroleum ether/ethyl acetate (10 : 1) to afford the corresponding product **18a-b**.

1-(2-Chloroquinolin-3-yl)ethan-1-ol (18a)

Yellow solid (78.5%). ^{1}H NMR (300 MHz, $CDCl_3$) δ 8.42 (s, 1 H), 8.05 (d, *J* = 8.4 Hz, 1 H), 7.86 (d, *J* = 8.1 Hz, 1 H), 7.73 (d, *J* = 7.5 Hz, 1 H), 7.58 (d, *J* = 7.5 Hz, 1 H), 5.37 (q, *J* = 6.0 Hz, 1 H), 2.33 (br s, 1 H), 1.62 (d, *J* = 6.3 Hz, 3 H). HRMS (ESI, m/z)$[M+H]^+$: 208.0562.

(2-Chloroquinolin-3-yl) (phenyl)methanol (18b)

White solid (76.4%). ^{1}H NMR (300 MHz, Acetone-d_6) δ 8.70 (s, 1 H), 8.08 (d, *J* = 8.4 Hz, 1 H), 7.94 (d, *J* = 8.1 Hz, 1 H), 7.80 (d, *J* = 7.5 Hz, 1 H), 7.66 (t, *J* = 7.2 Hz, 1 H), 7.44 (t, *J* = 7.2 Hz, 2 H), 7.33 (d, *J* = 7.5 Hz, 2 H), 7.27 (t, *J* = 7.2 Hz, 1 H), 6.23 (s, 1 H), 5.30 (d, *J* = 4.2 Hz, 1 H). HRMS (ESI, m/z)$[M+H]^+$: 270.0689.

*General Procedure for the Synthesis of Compounds **19a-b**:*

To a solution of (2-chloroquinolin-3-yl)methanol (1 equiv) in 30 mL of anhydrous dichloromethane Dess-Martin periodinane (1.5 equiv) was added. The resulting mixture was stirred at room temperature for 1 h and then was quenched with saturated aqueous sodium thiosulfate (40 mL) and saturated aqueous sodium bicarbonate (40 mL). The organic layer was separated and the aqueous phase was extracted with dichloromethane (30 mL×2). The combined organic layers were dried over anhydrous Na_2SO_4, filtered and concentrated. The residue was purified by flash column chromatography with petroleum ether/ethyl acetate (20 : 1) to afford the desired product.

1-(2-Chloroquinolin-3-yl)ethan-1-one (19a)

White solid (92.7%). ^{1}H NMR (300 MHz, $CDCl_3$) δ 8.40 (s, 1 H), 8.06 (d, J = 8.4 Hz, 1 H), 7.90 (d, J = 7.8 Hz, 1 H), 7.84 (d, J = 7.5 Hz, 1 H), 7.63 (d, J = 7.5 Hz, 1 H), 2.78 (s, 3 H). HRMS (ESI, m/z)$[M+H]^+$: 206.0380

(2-Chloroquinolin-3-yl) (phenyl)methanone (19b)

White solid (95.5%). ^{1}H NMR (300 MHz, Acetone-d_6) δ 8.55 (s, 1 H), 8.13 (d, J = 8.4 Hz, 1 H), 8.07 (d, J = 8.7 Hz, 1 H), 7.98−7.90 (m, 3 H), 7.79−7.71 (m, 2 H), 7.59 (d, J = 7.5 Hz, 2 H). HRMS (ESI, m/z)$[M+H]^+$: 268.0530

General Procedure for the Synthesis of Compounds ***20a-b****:*

To a solution of 2-chloroquinoline (1 equiv) and 1-naphthol (1.5 equiv) in 15 mL of anhydrous DMSO K_2CO_3 (3 equiv) was added and the reaction mixture was stirred at 100 °C for 4 h. After cooling down to room temperature, the mixture was poured into water (40 mL), extracted with dichloromethane (30 mL×2), dried with anhydrous Na_2SO_4, filtered and concentrated. The residue was purified by flash column chromatography with petroleum ether/ethyl acetate (15 : 1) to afford the corresponding product.

1-(2-(Naphthalen-1-yloxy)quinolin-3-yl)ethan-1-one (20a)

Yellow solid (86.9%). ^{1}H NMR (300 MHz, Acetone-d_6) δ 8.81 (s, 1 H), 8.11−8.02 (m, 3 H), 7.90 (d, J = 8.4 Hz, 1 H), 7.67 (d, J = 7.5 Hz, 1 H), 7.60−7.48 (m, 6 H), 2.92 (s, 3 H). HRMS (ESI, m/z)$[M+H]^+$: 314.1139.

(2-(Naphthalen-1-yloxy)quinolin-3-yl) (phenyl)methanone (20b)

Yellow solid (92.9%). ^{1}H NMR (300 MHz, Acetone-d_6) δ 8.59 (s, 1 H), 8.12−8.04 (m, 3 H), 7.95 (d, J = 8.4 Hz, 1 H), 7.82 (d, J = 7.5 Hz, 1 H), 7.74−7.68 (m, 2 H), 7.66−7.59 (m, 2 H), 7.59−7.49 (m, 4 H), 7.48−7.38 (m, 3 H). HRMS (ESI, m/z)$[M+H]^+$: 376.1305.

(E)-1-(2-(Naphthalen-1-yloxy)quinolin-3-yl)ethan-1-one oxime (21)

To a solution of **20a** (0.88 g, 2.81 mmol) and Hydroxylamine hydrochloride (0.29g, 4.23 mmol) in 10 mL of anhydrous ethanol was added Et_3N (0.43 g, 4.23 mmol). The reaction mixture was stirred at room temperature overnight and concentrated. Water (10 mL) was added and the mixture was extracted with dichloromethane (20 mL×2), dried with Na_2SO_4, filtered and concentrated. The residue was recrystallized from petroleum ether and ethyl

acetate to afford a white solid (780 mg, 84.6%). Mp 89−90 ºC. ^{1}H NMR (300 MHz, DMSO-d_6) δ 11.48 (s, 1 H), 8.43 (s, 1 H), 8.06−7.98 (m, 2 H), 7.87 (d, J = 8.7 Hz, 1 H), 7.82 (d, J = 7.8 Hz, 1 H), 7.61−7.52 (m, 3 H), 7.50−7.38 (m, 4 H), 2.36 (s, 3 H). HRMS (ESI, m/z)[M+H]$^+$: 329.1286.

(E)-1-(2-(Naphthalen-1-yloxy)quinolin-3-yl)ethan-1-one O-(2-(dimethylamino)ethyl) oxime (22)

To a solution of **21** (250 mg, 0.75 mmol) in 5 mL of DMF NaH (100 mg, 2.5 mmol) was added. The reaction mixture was stirred at room temperature for 10 min and 2-Dimethylaminoethyl chloride hydrochloride (125mg, 0.85 mmol) was added. The reaction mixture was stirred at room temperature for 5 h. Water (10 mL) was added and the mixture was extracted with dichloromethane (10 mL×2), dried with Na_2SO_4, filtered and concentrated. The residue was purified by flash column chromatography with dichloromethane/methanol (20:1) to afford a yellow oil (200 mg, 66.8%). Mp 213−214 ºC. ^{1}H NMR (300 MHz, Acetone-d_6) δ 8.41 (s, 1 H), 8.05−7.96 (m, 3 H), 7.87 (d, J = 8.4 Hz, 1 H), 7.61−7.55 (m, 3 H), 7.55−7.45 (m, 4 H), 4.34 (t, J = 6.0 Hz, 2 H), 2.67 (t, J = 6.0 Hz, 2 H), 2.48 (s, 3 H), 2.26 (s, 6 H). ^{13}C NMR (100 MHz, Acetone-d_6) δ 160.39, 154.34, 150.46, 146.88, 139.77, 135.84, 131.00, 128.82, 128.45, 127.76, 127.10, 126.95, 126.61, 126.50, 126.01, 125.87, 123.72, 122.63, 118.97, 73.43, 58.87, 46.14, 16.00. HRMS (ESI-TOF, m/z): calcd for $C_{25}H_{26}N_3O_2$ [M+H]$^+$ 400.20195; found: 400.2027.

4-(Dimethylamino)-1-(2-(naphthalen-1-yloxy)quinolin-3-yl)-1-phenylbutan-1-ol (23)

The (3-(dimethylamino)propyl)magnesium chloride (1 g, 8.3 mmol) prepared in sodium-dried ether was added dropwise to Compound **20b** (600 mg, 1.60 mmol) in anhydrous tetrahydrofuran at 0 °C and stirred at room temperature for 2h. Then the reaction was quenched by 20 mL saturated NH_4Cl, extracted by ethyl acetate (20 mL×3), dried over anhydrous Na_2SO_4, filtered and concentrated. The residue was purified by flash column chromatography with dichloromethane/methanol (20:1) to afford a yellow solid (707 mg, 95.7%). ^{1}H NMR (300 MHz, Acetone-d_6) δ 8.91 (s, 1 H), 8.04 (d, J = 8.1 Hz, 1 H), 7.92 (d, J = 8.1 Hz, 1 H), 7.78 (d, J = 8.1 Hz, 1 H), 7.61 (d, J = 7.2 Hz, 2 H), 7.56−7.47 (m, 4 H), 7.37−7.27 (m, 4 H), 7.19 (d, J = 7.5 Hz, 1 H), 7.02−6.94 (m, 2 H), 3.32−3.19 (m, 1 H), 2.66−2.55 (m, 1 H), 2.55−2.37 (m, 2 H), 2.21 (s, 6 H), 1.79−1.61 (m, 2 H). ^{13}C NMR (100 MHz, Acetone-d_6) δ 160.25, 150.52, 148.19, 146.34, 137.81, 135.75, 133.04, 130.01, 128.79,

128.76, 128.52, 127.76, 127.45, 127.30, 127.08, 126.97, 126.55, 126.51, 125.61, 125.58, 122.99, 118.92, 76.27, 60.80, 45.30, 38.33, 23.35. HRMS (ESI-TOF, m/z): calcd for $C_{31}H_{31}N_2O_2$ $[M+H]^+$ 463.2380; found: 463.2367.

(E)-N, N-Dimethyl-4-(2-(naphthalen-1-yloxy)quinolin-3-yl)-4-phenylbut-3-en-1-amine hydrochloride (24)

Compound **23** (0.60 g, 5.62 mmol) was added to HCl/EtOH (15 mL) and the mixture was heated to reflux at 90 °C oil bath for 4 h. The reaction mixture was concentrated, purified by flash column chromatography with dichloromethane/methanol (20:1) and recrystallized from acetone to afford a white solid (0.42 g, 72.7%). Mp 62−63 ºC. ^{1}H NMR (300 MHz, CD3OD) δ 8.29 (s, 1 H), 7.91 (d, J = 8.1 Hz, 1 H), 7.80 (d, J = 7.8 Hz, 1 H), 7.67 (d, J = 8.1 Hz, 1 H), 7.60−7.51 (m, 2 H), 7.45 (d, J = 5.7 Hz, 1 H), 7.41−7.29 (m, 7 H), 7.19−7.08 (m, 3 H), 6.29 (d, J = 7.5 Hz, 1 H), 3.31 (d, J = 7.5 Hz, 2 H), 2.81 (s, 6 H), 2.77−2.63 (m, 2 H). ^{13}C NMR (100 MHz, CD3OD) δ 161.05, 150.61, 147.34, 142.29, 142.12, 142.08, 136.35, 131.35, 129.71, 129.08, 128.84, 128.60, 128.07, 127.90, 127.32, 127.23, 126.87, 126.55, 126.02, 125.86, 124.80, 122.69, 118.39, 58.21, 43.56, 26.94. HRMS (ESI-TOF$^+$): m/z calcd for $C_{31}H_{29}NO_2$ $[M+H]^+$ 445.2274; found: 445.2278.

Series 4

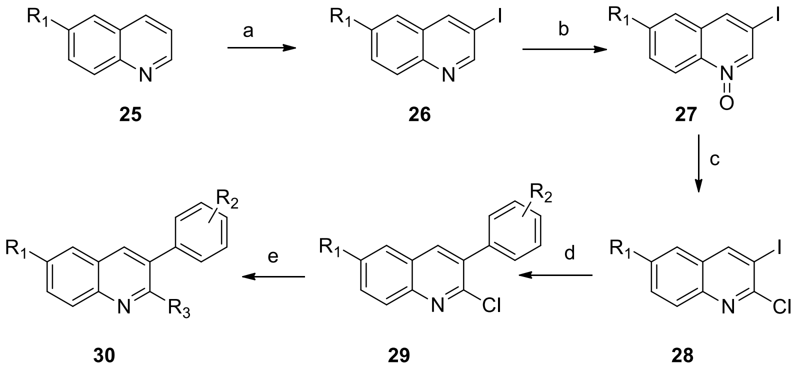

Scheme 5. Synthesis of series 4 compounds. Reagents and conditions: (a) NIS, AcOH, 100 °C, 28 h; (b) mCPBA, $CHCl_3$, 3 h, RT; (c) $POCl_3$, $CHCl_3$, 3 h, reflux; (d) (4-((dimethylamino)methyl)phenyl) boronic acid, $Pd(PPh_3)_4$, Na_2CO_3, toluene/H_2O, 14 h; (e) NaH , THF, RT, 6 h.

*General Procedure for the Synthesis of Compounds **26a-b**:*

To a solution of quinoline **25** (1 equiv) in 40 mL of acetic acid NIS (1.5 equiv) was added in batches and the reaction mixture was stirred at 100 °C for 20 h. After cooling down to room temperature, the mixture was poured into water (150 mL) and extracted with dichloromethane (50 mL×3), The combined organic phase was washed with 20 mL saturated $NaHCO_3$, dried with anhydrous Na_2SO_4, filtered and concentrated. The residue was purified by flash column chromatography with petroleum ether/ethyl acetate (3:1) to afford the corresponding product.

6-Bromo-3-iodoquinoline (26a)

Yellow solid (47.4%). ^{1}H NMR (300 MHz, $CDCl_3$) δ 9.03 (d, J = 2.1 Hz, 1 H), 8.45 (d, J = 1.5 Hz, 1 H), 7.93 (d, J = 9.0 Hz, 1 H), 7.87 (d, J = 2.1 Hz, 1 H), 7.79 (dd, J = 9.0, 2.1 Hz, 1 H). HRMS (ESI, m/z)$[M+H]^+$: 333.8715.

3-Iodo-6-methoxyquinoline (26b)

Yellow solid (46.9%). ^{1}H NMR (400 MHz, Acetone-d_6) δ 8.76 (d, J = 3.4 Hz, 1 H), 8.43 (d, J = 8.6 Hz, 1 H), 8.10 (d, J = 9.2 Hz, 1 H), 7.68 (d, J = 9.2 Hz, 1 H), 7.55 (dd, J = 8.5, 4.0 Hz, 1 H), 4.08 (s, 3 H). HRMS (ESI, m/z)$[M+H]^+$: 285.9707.

*General Procedure for the Synthesis of Compounds **27a-b**:*

To a solution of 3-iodoquinoline (1 equiv) in 40 mL of chloroform 3-Chloroperbenzoic acid (1.5 equiv) was added in batches and the reaction mixture was stirred at room temperature for 6 h. Then 100 mL saturated $NaHCO_3$ solution were added. After stirring for 30 min, 2N NaOH solution (50 mL) was added. The organic layer was separated and the aqueous layer extracted with dichloromethane (20 mL×2), dried with anhydrous Na_2SO_4 and concentrated to afford the corresponding product.

6-Bromo-3-iodoquinoline 1-oxide (27a)

White solid (92.7%). ^{1}H NMR (400 MHz, $CDCl_3$) δ 8.73 (s, 1 H), 8.52 (d, J = 9.2 Hz, 1 H), 7.99 (s, 1 H), 7.92 (s, 1 H), 7.81 (d, J = 9.2 Hz, 1 H,) . HRMS (ESI, m/z)$[M+H]^+$: 349.8666.

3-Iodo-6-methoxyquinoline 1-oxide (27b)

Yellow solid (90.6%). ^{1}H NMR (400 MHz, Acetone-d_6) δ 8.70 (d, J = 9.6 Hz, 1 H), 8.41 (d, J = 6.0 Hz, 1 H), 7.93 (d, J = 8.9 Hz, 1 H), 7.65 (d, J = 9.6 Hz, 1 H), 7.51 (dd, J = 8.6, 6.2 Hz, 1 H), 4.11 (s, 3 H). HRMS (ESI, m/z)[M+H]$^+$: 301.9654.

*General Procedure for the Synthesis of Compounds **28a-b**:*

The **2-chloro-3-iodoquinoline 28c** was purchased from was purchased from a commercial source.

To a solution of 3-iodoquinoline 1-oxide (1 equiv) in 40 mL of chloroform was added $POCl_3$ (2 equiv) and the reaction mixture was stirred in reflux for 6 h. Then 100 mL saturated $NaHCO_3$ was added, After 30 min of stirring, the organic layer was separated and the aqueous layer extracted with dichloromethane (40 mL×2), dried with anhydrous Na_2SO_4 and concentrated. The crude product was purified by flash column chromatography with petroleum ether / dichloromethane (4:1) to afford the corresponding product.

6-Bromo-2-chloro-3-iodoquinoline (28a)

White solid (72.9%). ^{1}H NMR (400 MHz, $CDCl_3$) δ 8.58 (s, 1 H), 7.89 (s, 1 H), 7.86 (d, J = 8.8 Hz, 1 H), 7.81 (d, J = 8.8 Hz, 1 H,) . HRMS (ESI, m/z)[M+H]$^+$: 367.8326.

2-Chloro-3-iodo-6-methoxyquinoline (28b)

White solid (85.7%). ^{1}H NMR (400 MHz, Acetone-d_6) δ 8.47 (d, J = 8.9 Hz, 1 H), 8.00 (d, J = 9.2 Hz, 1 H), 7.71 (d, J = 9.3 Hz, 1 H), 7.56 (d, J = 8.9 Hz, 1 H), 4.09 (s, 3 H). HRMS (ESI, m/z)[M+H]$^+$: 319.9317.

6-Bromo-2-chloro-3-(3-((dimethylamino)methyl)phenyl)quinoline (29a)

To a solution of 6-bromo-2-chloro-3-iodoquinoline **28a** (128 mg, 0.35 mmol) in the mixture of 3 mL toluene and 2mL H_2O Pd $(PPh_3)_4$ (12 mg, 0.01 mmol), Na_2CO_3 (73 mg, 0.69 mmol) and (3-((dimethylamino)methyl)phenyl)boronic acid (124 mg, 0.69 mmol) was added. After stirring at 80 °C for 10 h, water (10 mL) was added and the mixture was extracted with dichloromethane (10 mL×2), dried with Na_2SO_4 and concentrated. The residue was purified by flash column chromatography with dichloromethane/methanol (20:1) to give a yellow solid (111 mg, 84.8%). ^{1}H NMR (400 MHz, Acetone-d_6) δ 8.36 (s, 1 H), 8.30 (s, 1 H), 7.95 (s,

2 H), 7.56 (s, 1 H), 7.51−7.41 (m, 3 H), 3.53 (s, 2 H), 2.24 (s, 6 H) . HRMS (ESI, m/z)[M+H]$^+$: 375.0266.

6-Bromo-2-chloro-3-(3-((dimethylamino)ethyl)phenyl)quinoline (29b)

The procedure used to synthesize **29a** was repeated using 6-bromo-2-chloro-3-iodoquinoline **28a** and (3-((dimethylamino)ethyl)phenyl)boronic acid to afford compound **29b** as a yellow solid in 88.5% yield. The residue was purified by flash column chromatography with dichloromethane/methanol (20:1). ^{1}H NMR (400 MHz, Acetone-d_6) δ 8.34 (s, 1 H), 8.29 (s, 1 H), 7.94 (s, 2 H), 7.48 (s, 1 H), 7.46−7.40 (m, 2 H), 7.37(d, J = 6.8 Hz, 1 H), 2.86 (t, J = 7.6 Hz, 2 H), 2.57 (t, J = 7.6 Hz, 2 H), 2.24 (s, 6 H). HRMS (ESI, m/z)[M+H]$^+$: 389.0419.

2-Chloro-6-methoxy-3-(3-((dimethylamino)methyl)phenyl)quinoline (29c)

The procedure used to synthesize **29a** was repeated using 2-chloro-3-iodo-6-methoxyquinoline **28b** and (3-((dimethylamino)methyl)phenyl)boronic acid to afford compound **29c** as a yellow solid in 76.3% yield. The residue was purified by flash column chromatography with dichloromethane/methanol (20:1). ^{1}H NMR (400 MHz, Acetone-d_6) δ 8.48 (s, 1 H), 7.96−7.84 (m, 2 H), 7.74 (d, J = 7.0 Hz, 1 H), 7.66 (d, J = 7.5 Hz, 1 H), 7.56 (t, J = 7.7 Hz, 1 H), 7.51−7.40 (m, 2 H), 4.15 (s, 2 H), 3.96 (s, 3 H), 2.63 (s, 6 H). HRMS (ESI, m/z)[M+H]$^+$: 327.1251.

2-Chloro-3-(3-((dimethylamino)methyl)phenyl)quinoline(29d)

The procedure used to synthesize **29a** was repeated using 2-chloro-3-iodoquinoline **28c** and (3-((dimethylamino)methyl)phenyl)boronic acid to afford compound **29d** as a yellow oil in 88.5% yield. The residue was purified by flash column chromatography with dichloromethane/methanol (20:1). ^{1}H NMR (400 MHz, Acetone-d_6) δ 8.35 (s, 1 H), 8.06 (d, J = 8.1 Hz, 1 H), 8.00 (d, J = 8.5 Hz, 1 H), 7.87−7.80 (m, 1 H), 7.68 (dd, J = 11.1, 4.0 Hz, 1 H), 7.55 (s, 1 H), 7.47 (d, J = 5.2 Hz, 2 H), 7.43 (dd, J = 7.9, 3.9 Hz, 1 H), 3.51 (s, 2 H), 2.23 (s, 6 H). HRMS (ESI, m/z)[M+H]$^+$: 297.1171.

2-Chloro-3-(3-(dimethylcarbamoyl)phenyl)quinoline (29e)

The procedure used to synthesize **29a** was repeated using **28c** and (3-(dimethylcarbamoyl)phenyl)boronic acid to afford compound **29e** as a white solid in 82.0% yield. The residue was purified by flash column chromatography with petroleum ether/ethyl

acetate (3:1). ^{1}H NMR (400 MHz, Acetone-d_6) δ 8.42 (s, 1 H), 8.08 (d, J = 8.4 Hz, 1 H), 8.02 (d, J = 8.4 Hz, 1 H), 7.86 (t, J = 7.6 Hz, 1 H), 7.72−7.64 (m, 3 H), 7.60 (t, J = 7.6 Hz, 1 H), 7.55 (d, J = 7.6 Hz, 1 H), 3.07 (s, 6 H). HRMS (ESI, m/z)$[M+H]^+$: 311.0941.

6-bromo-2-chloro-3-phenylquinoline (29f)

The procedure used to synthesize **29a** was repeated using **28a** and phenylboronic acid to afford compound **29f** as a white solid in 90.8% yield. The residue was purified by flash column chromatography with petroleum ether/ethyl acetate (10:1). ^{1}H NMR (400 MHz, Acetone-d_6) δ 8.34 (d, J = 1.3 Hz, 1H), 8.29 (d, J = 1.2 Hz, 1H), 7.94 (s, 2H), 7.63−7.57 (m, 2H), 7.57−7.46 (m, 3H). HRMS (ESI, m/z)$[M+H]^+$: 319.9641.

6-Bromo-2-chloro-3-(3-(pyridin-2-yloxy)phenyl)quinoline (29g)

The procedure used to synthesize **29a** was repeated using **28a** and (3-(pyridin-2-yloxy)phenyl)boronic acid to afford compound **29g** as a white solid in 80.4% yield. The residue was purified by flash column chromatography with petroleum ether/ethyl acetate (8:1). ^{1}H NMR ((400 MHz, Acetone-d_6) δ 8.39 (s, 1 H), 8.29 (s, 1 H), 8.17 (dd, J = 4.8, 1.6 Hz, 1 H), 7.94 (d, J = 1.2 Hz, 2 H), 7.89−7.83 (m, 1 H), 7.58 (t, J = 7.9 Hz, 1 H), 7.44 (d, J = 7.7 Hz, 1 H), 7.40 (t, J = 2.0 Hz, 1 H), 7.28 (ddd, J = 8.2, 2.2, 0.8 Hz, 1 H), 7.13 (dd, J = 6.8, 5.3 Hz, 1 H), 7.06 (d, J = 8.3 Hz, 1 H). HRMS (ESI, m/z)$[M+H]^+$: 412.9872.

6-Bromo-2-chloro-3-(3-(oxazol-2-yl)phenyl)quinoline (29h)

The procedure used to synthesize **29a** was repeated using **28a** and (3-(oxazol-2-yl)phenyl)boronic acid to afford compound **29h** as a white solid in 91.5% yield. The residue was purified by flash column chromatography with petroleum ether/ethyl acetate (5:1). ^{1}H NMR (400 MHz, Acetone-d_6) δ 8.47 (s, 1 H), 8.34 (s, 1 H), 8.26 (s, 1 H), 8.17 (dt, J = 7.6, 1.5 Hz, 2 H), 8.08 (s, 1 H), 7.98 (s, 1 H), 7.97 (s, 1 H), 7.76 (dt, J = 7.7, 1.5 Hz, 2 H), 7.71 (t, J = 7.7 Hz, 1 H), 7.35 (s, 1 H).m/z$[M+H]^+$: 384.9736.

6-Bromo-3-(3-((dimethylamino)methyl)phenyl)-2-(naphthalen-1-ylmethoxy)quinoline (30a)

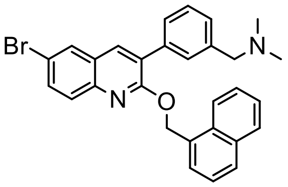

To a solution of **29a** (113 mg, 0.30 mmol) and 1-Naphthalenemethanol (68 mg, 0.43 mmol) in 4 mL DMSO was added Cs_2CO_3 (140 mg, 0.43 mmol). After stirring at 80 °C for 8 h, water (20 mL) was added and the mixture was extracted with dichloromethane (10 mL×3), dried with Na_2SO_4 and concentrated. The residue was purified by flash column chromatography with petroleum ether/ethyl acetate/triethylamine (40:15:1) to afford **30a** as a white solid (127 mg, 88.9%). Mp 87−88 ºC. ^{1}H NMR (400 MHz, Acetone-*d*$_6$) δ 8.20 (d, *J* = 8.2 Hz, 1 H), 8.17 (s, 1 H), 8.10 (d, *J* = 2.0 Hz, 1 H), 7.95 (d, *J* = 7.5 Hz, 1 H), 7.89 (d, *J* = 8.3 Hz, 1 H), 7.85 (d, *J* = 8.9 Hz, 1 H), 7.77 (dd, *J* = 8.9, 2.1 Hz, 1 H), 7.71 (d, *J* = 6.9 Hz, 1 H), 7.61−7.53 (m, 3 H), 7.50−7.41 (m, 2 H), 7.29 (t, *J* = 7.5 Hz, 1 H), 7.25 (d, *J* = 7.6 Hz, 1 H), 6.03 (s, 2 H), 3.23 (s, 2 H), 2.02 (s, 6 H). ^{13}C NMR (100 MHz, Acetone-*d*$_6$) δ 160.22, 145.27, 140.14, 138.15, 136.68, 134.77, 133.57, 133.29, 132.76, 130.86, 130.63, 129.67, 129.66, 129.45, 129.29, 128.82, 128.76, 128.39, 128.26, 127.98, 127.26, 126.71, 126.15, 124.91, 117.89, 67.16, 64.50, 45.38. HRMS(ESI-TOF, m/z): calcd for $C_{29}H_{26}BrN_2O$ $[M+H]^+$ 497.1223; found 497.1230.

3-(3-(2-(Dimethylamino)ethyl)phenyl-)-6-bromo-2-(naphthalen-1-ylmethoxy)quinoline (30b)

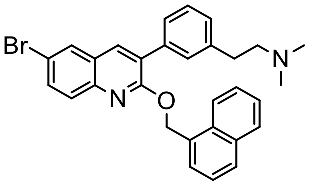

The procedure used to synthesize **30a** was repeated using **29b** and 1-naphthalenemethanol to afford compound **30b** as a white solid in 83.3% yield. Mp 90−91 ºC. ^{1}H NMR (400 MHz, Acetone-*d*$_6$) δ 8.25 (d, *J* = 7.8 Hz, 1 H), 8.21 (s, 1 H), 8.14 (s, 1 H), 7.96 (d, *J* = 8.0 Hz, 1 H), 7.91 (d, *J* = 8.4 Hz, 1 H), 7.88 (d, *J* = 8.8 Hz, 1 H), 7.80 (dd, *J* = 8.8, 1.6 Hz, 1 H), 7.74 (d, *J* = 7.5 Hz, 1 H), 7.62 (t, *J* = 7.6 Hz, 1 H), 7.56 (t, *J* = 7.6 Hz, 1 H), 7.52−7.46 (m, 2 H), 7.44 (d, *J* = 7.6 Hz, 1 H), 7.26 (t, *J* = 7.6 Hz, 1 H), 7.17 (d, *J* = 7.6 Hz, 1 H), 6.06 (s, 2 H), 2.62 (t, *J* = 8.0 Hz, 2 H), 2.32 (t, *J* = 7.6 Hz, 2 H), 2.09 (s, 6 H). ^{13}C NMR (150 MHz, Acetone-*d*$_6$) δ 160.25, 145.30, 140.55, 138.25, 136.96, 134.79, 133.65, 133.34, 132.81, 130.76, 130.65,

129.78, 129.70, 129.50, 129.27, 129.07, 128.43, 128.39, 128.04, 128.01, 127.32, 126.83, 126.23, 124.99, 117.91, 67.18, 60.97, 44.65, 33.60. HRMS (ESI-TOF, m/z): calcd for $C_{30}H_{28}{}^{81}BrN_2O$ $[M+H]^+$ 513.1359; found: 513.1364.

6-Bromo-3-(3-((dimethylamino)methyl)phenyl)-2-(2-(naphthalen-1-yl)ethoxy)quinoline (30c)

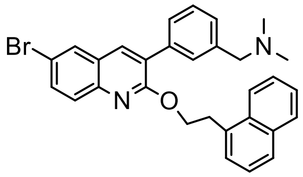

The procedure used to synthesize **30a** was repeated using **29a** and 1-naphthaleneethanol to afford compound **30c** as a yellow oil in 89.2% yield. Mp 77−78 ºC. ^{1}H NMR (300 MHz, Acetone-d_6) δ 8.40 (d, J = 8.3 Hz, 1 H), 8.17 (s, 1 H), 8.11 (d, J = 1.6 Hz, 1 H), 7.92 (d, J = 7.9 Hz, 1 H), 7.83−7.75 (m, 3 H), 7.55−7.43 (m, 8 H), 4.86 (t, J = 7.1 Hz, 2 H), 3.64 (t, J = 7.1 Hz, 2 H), 3.43 (s, 2 H), 2.21 (s, 6 H). ^{13}C NMR (75 MHz, Acetone-d_6) δ 160.44, 146.61, 140.36, 138.06, 136.88, 135.48, 134.97, 133.28, 133.15, 130.76, 130.64, 129.60, 129.57, 129.30, 129.01, 128.82, 128.43, 128.24, 128.05, 127.89, 126.91, 126.47, 126.43, 124.88, 117.76, 67.27, 64.68, 45.62, 32.92. HRMS (ESI-TOF, m/z): calcd for $C_{30}H_{28}BrN_2O$ $[M+H]^+$ 511.1380; found: 511.1389.

6-Bromo-3-(3-((dimethylamino)methyl)phenyl)-2-(naphthalen-1-ylamino)quinoline (30d)

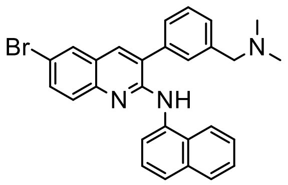

The procedure used to synthesize **30a** was repeated using **29a** and naphthalen-1-amine to afford compound **30d** as a yellow oil in 82.1% yield. Mp 55−56 ºC. ^{1}H NMR (400 MHz, Acetone-d_6) δ 8.66−8.58 (m, 1 H), 8.00 (d, J 1.2 Hz, 1 H), 7.99 (s, 1 H), 7.90 (d, J = 8.1 Hz, 1 H), 7.74 (s, 2 H), 7.66−7.60 (m, 6 H), 7.53 (t, J = 8.0 Hz, 2 H), 7.47 (t, J 7.4 Hz, 1 H), 7.40 (t, J = 7.6 Hz, 1 H), 3.55 (s, 2 H), 2.25 (s, 6 H). ^{13}C NMR (125 MHz, Acetone-d_6) δ 153.73, 146.59, 142.12, 137.62, 136.66, 136.35, 135.23, 133.14, 130.40, 130.37, 130.33, 130.15, 129.52, 129.45, 128.83, 128.82, 128.05, 126.72, 126.64, 126.57, 126.51, 124.40, 121.71,

119.31, 116.34, 64.55, 45.72. HRMS (ESI-TOF, m/z): calcd for $C_{28}H_{25}BrN_3$ $[M+H]^+$ 482.1226; found: 482.1220.

3-(3-((Dimethylamino)methyl)phenyl)-6-methoxy-2-(naphthalen-1-ylmethoxy)quinoline (30e)

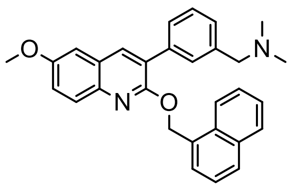

The procedure used to synthesize **30a** was repeated using **29c** and 1-naphthalenemethanol to afford compound **30e** as a yellow oil in 69.1% yield. Mp 63−64 °C. ^{1}H NMR (400 MHz, Acetone-d_6) δ 8.22 (d, J = 8.2 Hz, 1 H), 8.13 (s, 1 H), 7.95 (d, J = 7.9 Hz, 1 H), 7.89 (d, J = 8.3 Hz, 1 H), 7.85 (d, J = 9.8 Hz, 1 H), 7.72 (d, J = 6.9 Hz, 1 H), 7.60−7.49 (m, 4 H), 7.46 (t, J = 7.6 Hz, 1 H), 7.37−7.31 (m, 2 H), 7.29 (t, J = 7.5 Hz, 1 H), 7.24 (d, J = 7.5 Hz, 1 H), 6.02 (s, 2 H), 3.92 (s, 3 H), 3.25 (s, 2 H), 2.04 (s, 6 H). ^{13}C NMR (100 MHz, Acetone-d_6) δ 158.37, 157.45, 141.96, 140.01, 138.25, 137.34, 134.76, 134.03, 132.79, 130.89, 129.50, 129.41, 128.97, 128.95, 128.73, 128.06, 127.33, 127.31, 127.18, 126.66, 126.16, 124.96, 122.02, 107.19, 66.71, 64.54, 55.86, 45.38. HRMS (ESI-TOF, m/z): calcd for $C_{30}H_{29}N_2O_2$ $[M+H]^+$ 449.2224; found: 449.2218.

3-(3-((Dimethylamino)methyl)phenyl)-2-(naphthalen-1-yloxy)quinoline (30f)

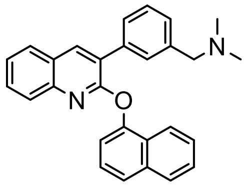

The procedure used to synthesize **30a** was repeated using **29d** and 1-naphthol to afford compound **30f** as a white solid in 78.9% yield. Mp 71−72 °C. ^{1}H NMR (400 MHz, Acetone-d_6) δ 8.43 (s, 1 H), 7.81−7.95 (m, 2 H), 7.94 (d, J = 8.5 Hz, 1 H), 7.90 (s, 1 H), 7.83 (t, J = 7.8 Hz, 2 H), 7.61−7.55 (m, 2 H), 7.55−7.45 (m, 5 H), 7.45−7.39 (m, 2 H), 3.53 (s, 2 H), 2.22 (s, 6 H). ^{13}C NMR (100 MHz, Acetone-d_6) δ 160.38, 151.04, 146.43, 140.23, 137.32, 135.92, 130.86, 130.51, 129.37, 129.08, 129.05, 128.86, 128.67, 128.57, 127.83, 127.51, 127.35, 127.12, 126.88, 126.71, 125.95, 125.64, 122.88, 118.89, 64.64, 45.56. MS(ESI-TOF, m/z): calcd for $C_{28}H_{25}N_2O$ $[M+H]^+$ 405.1961; found: 405.1960.

3-(3-(dimethylcarbamoyl)phenyl)-2-(naphthalen-1-yloxy)quinoline (30g)

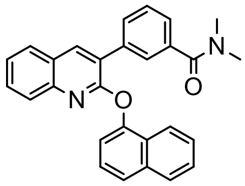

The procedure used to synthesize **30a** was repeated using **29e** and 1-naphthol to afford compound **30g** as a white solid in 99.3% yield. Mp 159−160 ºC. ^{1}H NMR (400 MHz, Acetone-d_6) δ 8.47 (s, 1 H), 8.05−7.96 (m, 4 H), 7.92 (d, J = 8.4 Hz, 1 H), 7.82 (d, J = 8.2 Hz, 1 H), 7.63−7.55 (m, 3 H), 7.54−7.44 (m, 5 H), 7.42 (t, J = 7.3 Hz, 1 H), 3.01 (s, 6 H). ^{13}C NMR (100 MHz, Acetone-d_6) δ 170.93, 160.24, 150.92, 146.55, 140.51, 138.12, 137.31, 135.88, 131.26, 130.70, 129.27, 129.16, 128.87, 128.75, 128.53, 127.81, 127.70, 127.26, 127.12, 126.94, 126.72, 126.61, 126.00, 125.77, 122.82, 119.00, 39.55, 35.19. HRMS (ESI-TOF, m/z): calcd for $C_{28}H_{23}N_2O_2$ $[M+H]^+$ 419.1754; found: 419.1758.

6-Bromo-2-(naphthalen-1-yloxy)-3-phenylquinoline (30h)

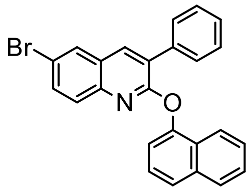

The procedure used to synthesize **30a** was repeated using **29f** and 1-naphthol to afford compound **30h** as a white solid in 70.6% yield. Mp 150-151 ºC. ^{1}H NMR (400 MHz, Acetone-d_6) δ 8.42 (s, 1 H), 8.20 (d, J = 2.0 Hz, 1 H), 8.00-7.95 (m, 3 H), 7.89 (d, J = 8.4 Hz, 1 H), 7.85 (d, J = 8.0 Hz, 1 H), 7.67 (dd, J = 8.8 2.4 Hz, 1 H), 7.60−7.54 (m, 3 H), 7.52−7.42 (m, 4 H), 7.39 (d, J = 8.8 Hz, 1 H). ^{13}C NMR (100 MHz, Acetone-d_6) δ 160.88, 150.78, 145.12, 139.40, 137.01, 135.92, 133.57, 130.69, 130.47, 129.84, 129.36, 129.23, 129.16, 128.92, 128.60, 128.50, 127.18, 127.04, 126.74, 125.97, 122.76, 119.13, 118.67. MS (ESI-TOF^{+}) : m/z calcd for $C_{25}H_{17}BrN_2O$: 426.0488; found: 426.0476.

6-Bromo-3-(3-((dimethylamino)methyl)phenyl)-2-(pyridin-4-yloxy)quinoline (30i)

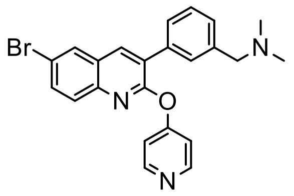

The procedure used to synthesize **30a** was repeated using **29a** and 4-Pyridinol to afford compound **30i** as a yellow solid in 84.8% yield. Mp 176−177 ºC. ^{1}H NMR (400 MHz, Acetone-d_6) δ 8.57 (s, 1 H), 8.38 (d, J = 2.0 Hz, 1 H), 8.03 (d, J = 9.0 Hz, 1 H), 7.99 (dd, J = 9.0, 2.1 Hz, 1 H), 7.81−7.75 (m, 1H), 7.75−7.71 (m, 1H), 7.46 (t, J = 7.6 Hz, 1 H), 7.43−7.36 (m, 3 H), 6.10− 6.06 (m, 1H), 6.06−6.02 (m, 1H), 3.42 (s, 2 H), 2.17 (s, 6 H). ^{13}C NMR (125 MHz, Acetone-d_6) δ 178.64, 152.25, 145.55, 141.01, 140.17, 137.00, 134.91, 131.72, 131.42, 130.92, 130.08, 129.96, 129.92, 128.32, 121.79, 118.16, 64.45, 45.57. HRMS (ESI-TOF, m/z): calcd for $C_{23}H_{21}BrN_3O$ [M+H]$^+$ 434.0863; found: 434.0866.

6-Bromo-3-(3-((dimethylamino)methyl)phenyl)-2-((3-methoxybenzyl)oxy)quinoline (30j)

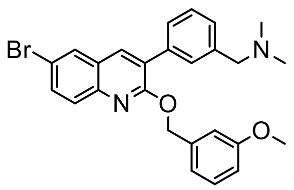

The procedure used to synthesize **30a** was repeated using **29a** and 3-methoxybenzyl alcohol to afford compound **30j** as a colorless oil in 74.3% yield. Mp 48-49 ºC. ^{1}H NMR (300 MHz, Acetone-d_6) δ 8.21 (s, 1 H), 8.13 (d, J = 1.7 Hz, 1 H), 7.81 (d, J = 8.9 Hz, 1 H), 7.77 (dd, J = 8.9, 2.0 Hz, 1 H), 7.69 (s, 1 H), 7.60 (d, J = 7.4 Hz, 1 H), 7.42 (t, J = 7.5 Hz, 1 H), 7.35 (d, J 7.6 Hz, 1 H), 7.27 (t, J = 8.1 Hz, 1 H), 7.08 (d, J = 6.6 Hz, 2 H), 6.86 (dd, J = 9.1, 1.7 Hz, 1 H), 5.57 (s, 2 H), 3.77 (s, 3 H), 3.45 (s, 2 H), 2.18 (s, 6 H). ^{13}C NMR (150 MHz, Acetone-d_6) δ 160.75, 160.24, 145.29, 140.44, 139.71, 138.14, 136.87, 133.30, 130.81, 130.66, 130.22, 129.62, 129.32, 128.89, 128.85, 128.43, 127.97, 120.79, 117.88, 114.16, 114.02, 68.58, 64.71, 55.46, 45.60. HRMS (ESI-TOF, m/z): calcd for $C_{26}H_{26}BrN_2O_2$ [M+H]$^+$ 477.1172; found: 477.1164.

6-Bromo-3-(3-((dimethylamino)methyl)phenyl)-2-((3-fluorobenzyl)oxy)quinoline (30k)

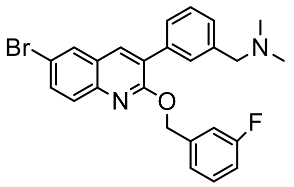

The procedure used to synthesize **30a** was repeated using **29a** and 3-fluorobenzyl alcohol to afford compound **30k** as a colorless oil in 78.5% yield. Mp 73-74 ºC. ^{1}H NMR (300 MHz, Acetone-d_6) δ 8.21 (s, 1 H), 8.13 (d, *J* = 1.2 Hz, 1 H), 7.84−7.73 (m, 2 H), 7.71 (s, 1 H), 7.59 (d, *J* = 7.4 Hz, 1 H), 7.48−7.32 (m, 4 H), 7.29 (d, *J* = 10.0 Hz, 1 H), 7.06 (t, *J* = 8.6 Hz, 1 H), 5.60 (s, 2 H), 3.46 (s, 2 H), 2.19 (s, 6 H). ^{13}C NMR (125 MHz, Acetone-d_6) δ 164.63, 162.69, 160.01, 145.19, 141.13, 141.07, 140.39, 138.23, 136.78, 133.34, 131.08, 131.02, 130.80, 130.65, 129.61, 129.38, 128.96, 128.82, 128.32, 128.00, 124.45, 124.43, 117.99, 115.39, 115.23, 115.21, 115.06, 67.90, 67.89, 64.68, 45.58. HRMS (ESI-TOF, m/z): calcd for $C_{25}H_{23}BrFN_2O$ [M+H]$^+$ 465.0972; found: 465.0975.

6-Bromo-3-(3-((dimethylamino)methyl)phenyl)-2-((3-hydroxyphenyl)thio)quinoline (30l)

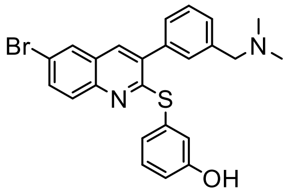

The procedure used to synthesize **30a** was repeated using **29a** and 3-mercaptophenol to afford compound **30l** as a yellow solid in 68.0% yield. Mp 95−96 ºC. ^{1}H NMR (400 MHz, Acetone-d_6) δ 8.15 (s, 1 H), 8.03 (s, 1 H), 7.75 (d, *J* = 8.9 Hz, 1 H), 7.61−7.56 (m, 2 H), 7.51−7.45 (m, 3 H), 7.27 (t, *J* = 7.9 Hz, 1 H), 7.11 (s, 1 H), 7.03 (d, *J* = 7.6 Hz, 1 H), 6.92 (d, *J* = 8.2 Hz, 1 H), 3.54 (s, 2 H), 2.27 (s, 6 H). ^{13}C NMR (100 MHz, Acetone-d_6) 160.18, 158.58, 146.61, 140.61, 138.26, 136.37, 135.62, 133.56, 132.15, 130.80, 130.74, 130.73, 130.55, 129.96, 129.24, 129.00, 128.59, 126.95, 122.72, 119.60, 116.84, 64.47, 45.55. HRMS (ESI-TOF, m/z): calcd for $C_{24}H_{22}BrN_2OS$ [M+H]$^+$ 465.0631; found: 465.0642.

6-Bromo-2-((3-fluorobenzyl)oxy)-3-(3-(pyridin-2-yloxy)phenyl)quinoline (30m)

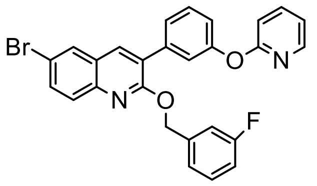

The procedure used to synthesize **30a** was repeated using **29g** and 3-fluorobenzyl alcohol to afford compound **30m** as a white solid in 91.8% yield. Mp 133−134 ºC. ^{1}H NMR (400 MHz, Acetone-d_6) δ 8.29 (s, 1 H), 8.15 (s, 1 H), 8.12 (dd, J = 4.9, 1.9 Hz, 1 H), 7.86−7.80 (m, 1 H), 7.80−7.75 (m, 2 H), 7.59 (d, J = 7.7 Hz, 1 H), 7.56−7.49 (m, 2 H), 7.40−7.31 (m, 2 H), 7.28 (d, J = 10.2 Hz, 1 H), 7.24−7.17 (m, 1 H), 7.10 (dd, J = 7.2, 5.0 Hz, 1 H), 7.08−6.97 (m, 2 H), 5.63 (s, 2 H). ^{13}C NMR (100 MHz, Acetone-d_6) δ 164.84, 164.40, 162.42, 159.84, 155.30, 148.39, 145.29, 141.15, 141.07, 140.63, 138.57, 138.39, 133.56, 131.11, 131.03, 130.76, 130.21, 129.63, 127.94, 127.50, 126.30, 124.41, 124.38, 123.17, 121.86, 119.74, 118.06, 115.40, 115.26, 115.18, 115.05, 112.49, 67.89.

HRMS (ESI-TOF, m/z): calcd for $C_{27}H_{19}BrFN_2O_2[M+H]^+$ 501.0597; found: 501.0616.

6-Bromo-2-((3-fluorobenzyl)oxy)-3-(3-(oxazol-2-yl)phenyl)quinoline (30n)

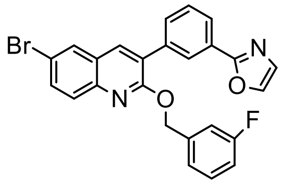

The procedure used to synthesize **30a** was repeated using **29h** and 3-fluorobenzyl alcohol to afford compound **30n** as a white solid in 87.6% yield. Mp 154−155 ºC. ^{1}H NMR (400 MHz, Acetone-d_6) δ 8.46 (s, 1 H), 8.37 (s, 1 H), 8.20 (s, 1 H), 8.10 (d, J = 7.8 Hz, 1 H), 8.06 (s, 1 H), 7.87 (d, J = 7.8 Hz, 1 H), 7.85−7.78 (m, 2 H), 7.65 (t, J = 7.8 Hz, 1 H), 7.45−7.37 (m, 2 H), 7.37−7.28 (m, 2 H), 7.11−6.98 (m, 1 H), 5.66 (s, 2 H). ^{13}C NMR (125 MHz, Acetone-d_6) δ 164.68, 162.74, 162.14, 159.88, 145.48, 141.07, 141.01, 140.50, 138.68, 137.74, 133.72, 132.19, 131.13, 131.07, 130.86, 129.94, 129.72, 129.53, 128.65, 128.12, 128.00, 127.37, 126.56, 124.52, 124.49, 118.16, 115.47, 115.29, 115.12, 68.06, 68.04. HRMS (ESI-TOF, m/z): calcd for $C_{25}H_{17}O_2N_2BrF$ $[M+H]^+$ 475.0452; found: 475.0445.

Second round of optimization

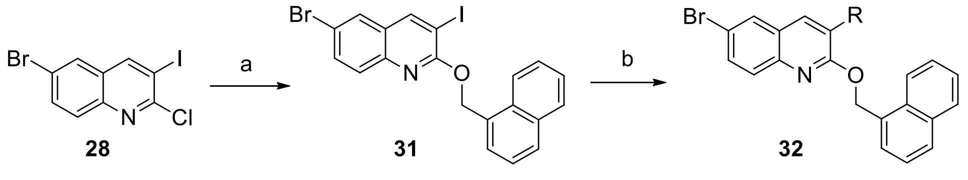

28 31 32

Scheme 6. Synthesis of compounds of 32. Reagents and conditions: (a) 1-Naphthalenemethanol, NaH , THF, RT, 6 h; (b) for Boric acid: $Pd(PPh_3)_4$, Na_2CO_3, toluene/H_2O, 14 h; for boronic acid pinacol cyclic ester: $Pd(dppf)Cl_2·CH_2Cl_2$, Na_2CO_3, toluene/H_2O, 14 h.

6-Bromo-3-iodo-2-(naphthalen-1-ylmethoxy)quinoline (31)

A solution of **28a** (1.00 g, 2.72 mmol) and 1-naphthalenemethanol (0.452 mg 2.86 mmol) in THF (20 mL) was treated with NaH (0.150 g, 4.08 mmol) and stirred at room temperature for 2h. Subsequently it was quenched with water (10 mL), the residue was extracted by dichloromethane (200 mL×3) and the combined organic layers were dried over anhydrous Na_2SO_4, filtered and concentrated. The residue was washed with methanol (10 mL) to give a white solid (1.24g, 93.41%).^{1}H NMR (400 MHz, DMSO) *δ* 8.86 (s, 1H), 8.15 (d, *J* = 7.5 Hz, 1H), 8.13 (s, 1H), 7.98 (d, *J* = 7.3 Hz, 1H), 7.95 (d, *J* = 8.4 Hz, 1H), 7.88–7.72 (m, 3H), 7.56 (dd, *J* = 13.0, 8.0 Hz, 3H), 5.97 (s, 2H). HRMS (ESI, m/z)$[M+H]^+$: 489.9322.

6-Bromo-3-(4-((dimethylamino)methyl)phenyl)-2-(naphthalen-1-ylmethoxy)quinoline (32a)

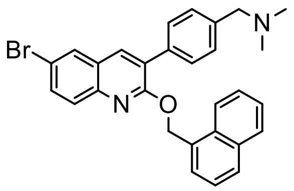

A mixture of 6-bromo-3-iodo-2-(naphthalen-1-ylmethoxy)quinoline **31** (100mg, 0.20 mmol), tetrakis(triphenylphosphine)platinum (12 mg, 0.01 mmol), Na_2CO_3 (43 mg, 0.41 mmol) and 3-((dimethylamino)methyl) phenylboronic acid (39 mg, 0.22 mmol) in the mixture of toluene (4 mL) and water (2mL) was stirred at 80 °C for 10 h. Water (10 mL) was added and the mixture was extracted with dichloromethane (10 mL×3), dried with Na_2SO_4 and concentrated. The residue was purified by flash column chromatography with petroleum ether/ethyl acetate/triethylamine (40:15:1) to afford **32a** as a white solid (87 mg, 87.3%). Mp 85−86 ºC. ^{1}H NMR (400 MHz, Acetone-d_6) *δ* 8.23 (d, *J* = 8.2 Hz, 1 H), 8.18 (s, 1 H), 8.11 (s, 1 H), 7.94 (d, *J* = 7.8 Hz, 1 H), 7.88 (d, *J* = 8.4 Hz, 1 H), 7.85 (d, *J* = 8.9 Hz, 1 H), 7.78 (dd, *J* = 8.8, 1.6 Hz, 1 H), 7.72 (d, *J* = 6.9 Hz, 1 H), 7.61−7.56 (m, 3 H), 7.54 (t, *J* = 6.8 Hz, 1 H), 7.46 (t, *J* = 7.6 Hz, 1 H), 7.29 (d, *J* = 7.9 Hz, 2 H), 6.07 (s, 2 H), 3.38 (s, 2 H), 2.17 (s, 6 H). ^{13}C NMR (100 MHz, Acetone-d_6) *δ* 160.22, 145.21, 140.31, 138.10, 135.47, 134.74, 133.66,

133.26, 132.68, 130.60, 130.07, 129.66, 129.64, 129.43, 129.33, 128.30, 128.11, 128.00, 127.13, 126.73, 126.15, 124.94, 117.89, 67.02, 64.33, 45.56. HRMS (ESI-TOF, m/z): calcd for $C_{29}H_{26}BrN_2O$ $[M+H]^+$ 497.1223; found: 497.1223.

3-(3-(2-(Dimethylamino)ethyl)phenyl-)-6-bromo-2-(naphthalen-1-ylmethoxy)quinoline (31b)

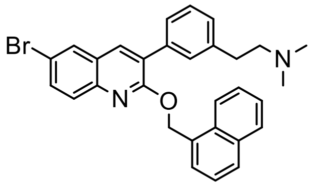

A mixture of 6-bromo-3-iodo-2-(naphthalen-1-ylmethoxy)quinoline **31** (100mg, 0.20 mmol), 1, 1'-Bis(diphenylphosphino)ferrocene-palladium(II)dichloride dichloromethane complex (8 mg, 0.01 mmol), Na_2CO_3 (43 mg, 0.41 mmol) and (3-(2-(dimethylamino)ethyl)phenyl) boronic acid pinacol ester (61 mg, 0.22 mmol) in the mixture of toluene (4 mL) and water (2mL) was stirred at 80 °C for 10 h. Water (10 mL) was added and the mixture was extracted with dichloromethane (10 mL×3), dried with Na_2SO_4 and concentrated. The residue was purified by flash column chromatography with dichloromethane/methanol (15:1) to afford **32c** (85 mg, 83.3%) as a white solid. Mp 76−77 ºC. ^1H NMR (400 MHz, Acetone-d_6) δ 8.25 (d, J = 7.8 Hz, 1 H), 8.21 (s, 1 H), 8.14 (s, 1 H), 7.96 (d, J = 8.0 Hz, 1 H), 7.91 (d, J = 8.4 Hz, 1 H), 7.88 (d, J = 8.8 Hz, 1 H), 7.80 (dd, J = 8.8, 1.6 Hz, 1 H), 7.74 (d, J = 7.5 Hz, 1 H), 7.62 (t, J = 7.6 Hz, 1 H), 7.56 (t, J = 7.6 Hz, 1 H), 7.51−7.46 (m, 2 H), 7.44 (d, J = 7.6 Hz, 1 H), 7.26 (t, J = 7.6 Hz, 1 H), 7.17 (d, J = 7.6 Hz, 1 H), 6.06 (s, 2 H), 2.62 (t, J = 8.0 Hz, 2 H), 2.32 (t, J = 7.6 Hz, 2 H), 2.09 (s, 6 H). ^{13}C NMR (150 MHz, Acetone-d_6) δ 160.25, 145.30, 140.55, 138.25, 136.96, 134.79, 133.65, 133.34, 132.81, 130.76, 130.65, 129.78, 129.70, 129.50, 129.27, 129.07, 128.43, 128.39, 128.04, 128.01, 127.32, 126.83, 126.23, 124.99, 117.91, 67.18, 60.97, 44.65, 33.60. HRMS (ESI-TOF, m/z): calcd for $C_{30}H_{28}{}^{81}BrN_2O$ $[M+H]^+$ 513.1359; found: 513.1364.

6-Bromo-3-(4-(2-(dimethylamino)ethyl)phenyl)-2-(naphthalen-1-ylmethoxy)quinoline (32b)

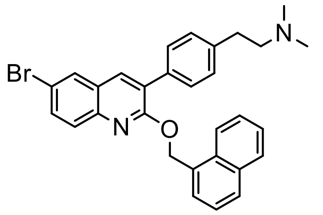

6-bromo-3-iodo-2-(naphthalen-1-ylmethoxy)quinoline **31** (100mg, 0.20 mmol), 1, 1'-Bis(diphenylphosphino)ferrocene-palladium(II)dichloride dichloromethane complex (8 mg, 0.01 mmol), Na_2CO_3 (43 mg, 0.41 mmol) and (3-(2-(dimethylamino)ethyl)phenyl)boronic acid pinacol ester (61 mg, 0.22 mmol) were mixed in the solvent of toluene (4 mL) and water (2mL), and the mixtures were stirred at 80 °C for 10 h. After the reaction, water (10 mL) was added and the mixture was extracted with dichloromethane (10 mL×3), dried with Na_2SO_4 and concentrated. The residue was purified by flash column chromatography with dichloromethane/methanol (15:1) to afford **32b** (89 mg, 87.3%) as a white solid. Mp 76−77 ºC. ^{1}H NMR (400 MHz, Acetone-*d*$_6$) *δ* 8.24 (d, *J* = 8.2 Hz, 1 H), 8.19 (s, 1 H), 8.12 (s, 1 H), 7.95 (d, *J* = 8.0 Hz, 1 H), 7.90 (d, *J* = 8.3 Hz, 1 H), 7.86 (d, *J* = 8.8 Hz, 1 H), 7.79 (d, *J* = 8.9 Hz, 1 H), 7.74 (d, *J* = 6.9 Hz, 1 H), 7.61 (t, *J* = 6.8 Hz, 1 H), 7.59−7.56 (m, 3 H), 7.48 (t, *J* = 7.6 Hz, 1 H), 7.22 (d, *J* = 7.9 Hz, 2 H), 6.09 (s, 2 H), 2.74 (t, *J* = 7.8 Hz, 2 H), 2.47 (t, *J* = 7.8 Hz, 2 H), 2.20 (s, 6 H). ^{13}C NMR (150 MHz, Acetone-*d*$_6$) *δ* 160.30, 145.19, 141.78, 138.08, 134.78, 134.45, 133.71, 133.25, 132.74, 130.60, 130.18, 129.68, 129.47, 129.37, 128.43, 128.20, 128.07, 127.18, 126.77, 126.20, 124.98, 117.90, 67.02, 61.93, 45.59, 34.40. HRMS (ESI-TOF, m/z): calcd for $C_{30}H_{28}{}^{81}BrN_2O$ $[M+H]^+$ 513.1359; found: 513.1367.

6-Bromo-3-(4-(2-(diethylamino)ethyl)phenyl)-2-(naphthalen-1-ylmethoxy)quinoline (32c)

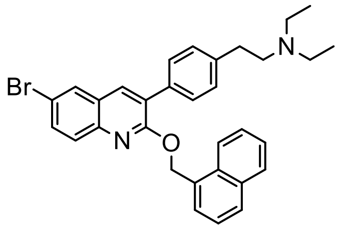

The procedure used to synthesize **32b** was repeated using **31** and N, N-diethyl-2-(4-(4, 4, 5, 5-tetramethyl-1, 3, 2-dioxaborolan-2-yl)phenyl)ethanamine to afford compound **32c** as a white solid in 71.1% yield. Mp 90−91 ºC. ^{1}H NMR (400 MHz, Acetone-*d*$_6$) *δ* 8.25 (d, *J* = 8.4 Hz, 1 H), 8.19 (s, 1 H), 8.13 (s, 1 H), 7.96 (d, *J* = 8.1 Hz, 1 H), 7.90 (d, *J* = 8.3 Hz, 1 H), 7.86 (d, *J* = 8.9 Hz, 1 H), 7.79 (d, *J* = 9.1 Hz, 1 H), 7.75 (d, *J* = 6.9 Hz, 1 H), 7.61 (t, *J* = 7.0 Hz, 1 H), 7.59–7.51 (m, 3 H), 7.48 (t, *J* = 7.6 Hz, 1 H), 7.22 (d, *J* = 7.8 Hz, 2 H), 6.09 (s, 2 H),

2.78−2.70 (m, 2 H), 2.70−2.62 (m, 2 H), 2.56 (q, J = 6.9 Hz, 4 H), 1.00 (t, J = 7.1 Hz, 6 H). ^{13}C NMR (150 MHz, Acetone-d_6) δ 160.31, 145.20, 141.98, 138.05, 134.79, 134.41, 133.72, 133.25, 132.73, 130.61, 130.18, 129.69, 129.67, 129.47, 129.44, 128.46, 128.17, 128.07, 127.18, 126.76, 126.20, 124.98, 117.90, 67.02, 55.51, 47.56, 33.95, 12.47. HRMS (ESI-TOF, m/z): calcd for $C_{32}H_{32}{}^{81}BrN_2O$ [M+H]$^+$ 541.1672; found: 541.1675.

6-Bromo-3-(4-(4-methylpiperazin-1-yl)phenyl)-2-(naphthalen-1-ylmethoxy)quinoline (32d)

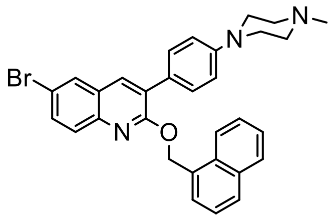

The procedure used to synthesize **32a** was repeated using **31** and (4-(4-methylpiperazin-1-yl)phenyl)boronic acid to afford compound **32d** as a white solid in 79.1% yield. Mp 132−133 ºC. ^{1}H NMR (400 MHz, Acetone-d_6) δ 8.26 (d, J = 8.5 Hz, 1 H), 8.14 (s, 1 H), 8.10 (d, J = 2.2 Hz, 1 H), 7.96 (d, J = 7.6 Hz, 1 H), 7.91 (d, J = 8.3 Hz, 1 H), 7.84 (d, J = 8.9 Hz, 1 H), 7.80−7.71 (m, 2 H), 7.65−7.59 (m, 1 H), 7.59−7.52 (m, 3 H), 7.49 (dd, J = 8.2, 7.1 Hz, 1 H), 6.95− 6.85 (m, 2 H), 6.09 (s, 2 H), 3.24−3.13 (m, 4 H), 2.51−2.41 (m, 4 H), 2.24 (s, 3 H). ^{13}C NMR (100 MHz, Acetone-d_6) δ 160.43, 152.09, 144.80, 136.96, 134.77, 133.80, 132.80, 132.73, 131.02, 130.39, 129.62, 129.61, 129.46, 128.44, 128.25, 128.15, 127.19, 126.81, 126.75, 126.22, 124.95, 117.79, 115.59, 66.92, 55.79, 48.99, 46.39. HRMS (ESI-TOF, m/z): calcd for $C_{31}H_{29}BrN_3O$ [M+H]$^+$ 538.1485; found: 538.1482.

6-Bromo-3-(4-(4-(2-(dimethylamino)ethyl)piperazin-1-yl)phenyl)-2-(naphthalen-1-ylmethoxy)quinoline (32e)

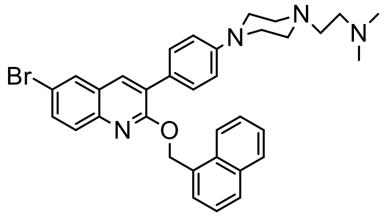

The procedure used to synthesize **32b** was repeated using **31** and (4-(4-(2-(dimethylamino)ethyl)piperazin-1-yl)phenyl)boronic acid pinacol ester to afford compound **32e** as a yellow solid in 56.1% yield. Mp 110−111 ºC. ^{1}H NMR (600 MHz, Acetone-d_6) δ

8.25 (d, *J* = 8.0 Hz, 1 H), 8.13 (s, 1 H), 8.09 (d, *J* = 2.2 Hz, 1 H), 7.96 (d, *J* = 8.1 Hz, 1 H), 7.90 (d, *J* = 8.3 Hz, 1 H), 7.83 (d, *J* = 8.8 Hz, 1 H), 7.78–7.73 (m, 2 H), 7.61 (ddd, *J* = 8.4, 6.8, 1.3 Hz, 1 H), 7.58–7.52 (m, 3 H), 7.48 (dd, *J* = 8.2, 7.0 Hz, 1 H), 6.89 (d, *J* = 8.9 Hz, 2 H), 6.09 (s, 2 H), 3.18 (t, *J* = 5.0 Hz, 4 H), 2.58 (t, *J* = 5.0 Hz, 4 H), 2.52−2.46 (m, 2 H), 2.46−2.37 (m, 2 H), 2.21 (s, 6 H). ^{13}C NMR (150 MHz, Acetone-d_6) δ 160.43, 152.11, 144.80, 136.96, 134.78, 133.81, 132.81, 132.73, 131.03, 130.40, 129.63, 129.62, 129.47, 128.45, 128.26, 128.14, 127.20, 126.79, 126.76, 126.23, 124.95, 117.80, 115.55, 66.91, 57.93, 57.22, 54.29, 49.12, 46.01. HRMS (ESI-TOF, m/z): calcd for $C_{34}H_{36}{}^{81}BrN_4O$ [M+H]$^+$ 597.2047; found: 597.2037.

6-Bromo-3-(4-((4-methylpiperazin-1-yl)methyl)phenyl)-2-(naphthalen-1-ylmethoxy)quinoline (32f)

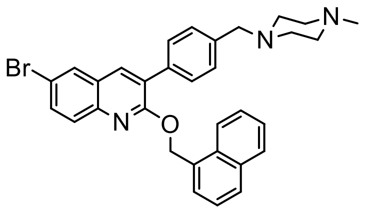

The procedure used to synthesize **32a** was repeated using **31** and (4-((4-methylpiperazin-1-yl)methyl)phenyl)boronic acid to afford compound **32f** as a yellow solid in 68.2% yield. Mp 61−62 ºC. ^{1}H NMR (400 MHz, Acetone-d_6) δ 8.24 (d, *J* = 8.1 Hz, 1 H), 8.19 (s, 1 H), 8.12 (s, 1 H), 7.95 (d, *J* = 7.7 Hz, 1 H), 7.89 (d, *J* = 8.2 Hz, 1 H), 7.86 (d, *J* = 9.0 Hz, 1 H), 7.79 (dt, *J* = 8.0, 1.6 Hz, 1 H), 7.73 (d, *J* = 7.0 Hz, 1 H), 7.67−7.51 (m, 4 H), 7.47 (t, *J* = 7.6 Hz, 1 H), 7.30 (d, *J* = 7.7 Hz, 2 H), 6.08 (s, 2 H), 3.46 (s, 2 H), 2.46−2.34 (m, 8 H), 2.18 (s, 3 H). ^{13}C NMR (150 MHz, Acetone-d_6) δ 160.25, 145.23, 139.72, 138.14, 135.51, 134.77, 133.67, 133.30, 132.70, 130.62, 130.11, 129.68, 129.68, 129.44, 128.33, 128.14, 128.02, 127.15, 126.75, 126.18, 124.98, 117.92, 67.04, 63.05, 55.97, 53.85, 46.32. HRMS (ESI-TOF, m/z): calcd for $C_{32}H_{31}BrN_3O$ [M+H]$^+$ 552.1645; found: 552.1653.

6-Bromo-3-(4-((4-benzylpiperazin-1-yl)methyl)phenyl)-2-(naphthalen-1-ylmethoxy)quinoline (32g)

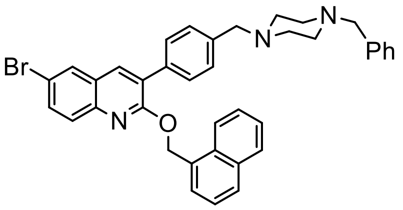

The procedure used to synthesize **32a** was repeated using **31** and (4-((4-benzylpiperazin-1-yl)methyl)phenyl)boronic acid to afford compound **32g** as a white solid in 55.7% yield. Mp 107−108 ºC. ^{1}H NMR (600 MHz, Acetone-d_6) δ 8.22 (d, J = 8.4 Hz, 1 H), 8.18 (s, 1 H), 8.11 (d, J = 1.8 Hz, 1 H), 7.93 (d, J = 8.1 Hz, 1 H), 7.88 (d, J = 8.3 Hz, 1 H), 7.85 (d, J = 8.8 Hz, 1 H), 7.78 (dd, J = 8.8, 1.9 Hz, 1 H), 7.72 (d, J = 7.0 Hz, 1 H), 7.63−7.57 (m, 3 H), 7.53 (t, J = 7.4 Hz, 1 H), 7.45 (t, J = 7.6 Hz, 1 H), 7.38−7.27 (m, 6 H), 7.23 (t, J = 6.8 Hz, 1 H), 6.07 (s, 2 H), 3.47 (s, 2 H), 3.46 (s, 2 H), 2.41 (br.s, 8 H). ^{13}C NMR (150 MHz, Acetone-d_6) δ 160.25, 145.24, 139.74, 139.67, 138.12, 135.50, 134.76, 133.67, 133.29, 132.71, 130.62, 130.11, 129.71, 129.69, 129.67, 129.44, 128.96, 128.34, 128.15, 128.02, 127.69, 127.14, 126.75, 126.17, 124.99, 117.91, 67.05, 63.49, 63.07, 53.97. HRMS (ESI-TOF, m/z): calcd for $C_{38}H_{35}{}^{81}BrN_3O$ $[M+H]^+$ 630.1943; found: 630.1937.

6-Bromo-3-(4-((4-(2-(dimethylamino)ethyl)piperazin-1-yl)methyl)phenyl)-2-(naphthalen-1-ylmethoxy)quinoline (32h)

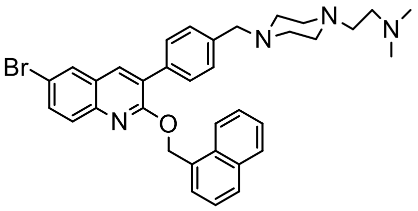

The procedure used to synthesize **32a** was repeated using **31** and (4-((4-(2-(dimethylamino)ethyl)piperazin-1-yl)methyl)phenyl)boronic acid to afford compound **32h** as a yellow oil in 67.2% yield. Mp 95−96 ºC. ^{1}H NMR (400 MHz, Acetone-d_6) δ 8.24 (d, J = 8.2 Hz, 1 H), 8.20 (s, 1 H), 8.12 (s, 1 H), 7.95 (d, J = 7.8 Hz, 1 H), 7.89 (d, J = 8.3 Hz, 1 H), 7.86 (d, J = 9.0 Hz, 1 H), 7.79 (d, J = 8.9 Hz, 1 H), 7.73 (d, J = 6.9 Hz, 1 H), 7.66−7.50 (m, 4 H), 7.47 (t, J = 7.7 Hz, 1 H), 7.30 (d, J = 7.9 Hz, 2 H), 6.08 (s, 2 H), 3.45 (s, 2 H), 2.63−2.27 (m, 12 H), 2.16 (s, 6 H). ^{13}C NMR (150 MHz, Acetone-d_6) δ 160.26, 145.24, 139.71, 138.14, 135.50, 134.77, 133.68, 133.30, 132.71, 130.62, 130.11, 129.69, 129.68, 129.45, 128.35, 128.15, 128.03, 127.15, 126.76, 126.18, 124.99, 117.92, 67.05, 63.11, 58.02, 57.42, 54.47,

54.03, 46.09. HRMS (ESI-TOF, m/z): calcd for $C_{35}H_{38}{}^{81}BrN_4O$ $[M+H]^+$ 611.2203; found: 611.2185.

6-Bromo-3-(4-(((2-(dimethylamino)ethyl)amino)methyl)phenyl)-2-(naphthalen-1-ylmethoxy)quinoline (32i)

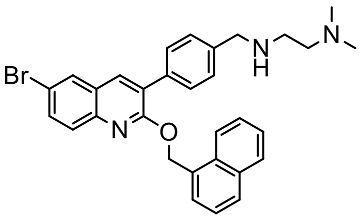

The procedure used to synthesize **32a** was repeated using **31** and (4-(((2-(dimethylamino)ethyl)amino)methyl)phenyl)boronic acid to afford compound **32i** as a yellow oil in 55.1% yield. Mp 85−86 ºC. ^{1}H NMR (400 MHz, Acetone-d_6) δ 8.24 (d, J = 8.3 Hz, 1 H), 8.18 (s, 1 H), 8.11 (s, 1 H), 7.95 (d, J = 7.9 Hz, 1 H), 7.89 (d, J = 8.3 Hz, 1 H), 7.85 (d, J = 8.9 Hz, 1 H), 7.78 (dd, J = 8.9, 2.1 Hz, 1 H), 7.73 (d, J = 6.9 Hz, 1 H), 7.67−7.51 (m, 4 H), 7.47 (t, J = 7.6 Hz, 1 H), 7.33 (d, J = 8.0 Hz, 2 H), 6.07 (s, 2 H), 3.77 (s, 2 H), 2.63 (t, J = 6.1 Hz, 2 H), 2.39 (t, J = 6.1 Hz, 2 H), 2.15 (s, 6 H). ^{13}C NMR (150 MHz, Acetone-d_6) δ 160.27, 145.21, 142.00, 138.10, 135.20, 134.76, 133.67, 133.26, 132.72, 130.60, 130.11, 129.67, 129.67, 129.45, 128.62, 128.39, 128.19, 128.03, 127.17, 126.76, 126.19, 124.95, 117.90, 67.04, 59.73, 53.90, 47.28, 45.64. HRMS (ESI-TOF, m/z): calcd for $C_{31}H_{31}{}^{81}BrN_3O$ $[M+H]^+$ 542.1625; found: 542.1635.

1-(4-(6-Bromo-2-(naphthalen-1-ylmethoxy)quinolin-3-yl)phenyl)-*N, N, N*-trimethylmethanaminium iodide (32j)

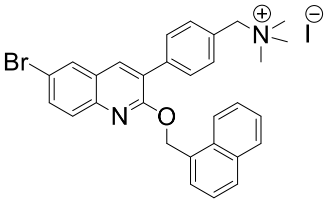

A solution of **32a** (100 mg, 0.20 mmol) in acetone (3 mL) was treated with MeI (0.10 mL, 1.6 mmol) and stirred at room temperature for 4 h. Then the suspension was concentrated. The residue was purified by flash column chromatography with dichloromethane/methano l (20:1) to afford **32j** as a white solid (102 mg, 79.9%). Mp 165−166 ºC. ^{1}H NMR (600 MHz, DMSO-d_6) δ 8.34 (s, 1 H), 8.25 (s, 1 H), 8.17 (d, J = 8.1 Hz, 1 H), 7.97 (d, J = 7.9 Hz, 1 H),

7.92 (d, *J* = 8.2 Hz, 1 H), 7.85 (s, 2 H), 7.74 (d, *J* = 7.8 Hz, 2 H), 7.68 (d, *J* = 6.9 Hz, 1 H), 7.62−7.53 (m, 2 H), 7.53−7.43 (m, 3 H), 6.02 (s, 2 H), 4.49 (s, 2 H), 2.99 (s, 9 H). ^{13}C NMR (150 MHz, DMSO-d_6) δ 158.65, 143.98, 138.01, 137.41, 133.26, 132.97, 132.54, 132.25, 131.25, 129.92, 129.68, 128.82, 128.65, 128.47, 127.99, 127.35, 126.63, 126.35, 125.98, 125.77, 125.35, 124.07, 116.99, 67.33, 66.19, 51.79. HRMS (ESI-TOF, m/z): calcd for $C_{30}H_{28}BrN_2O$ [M]$^+$ 511.1380; found: 511.1394.

6-Bromo-3-(4-(((3s, 5s, 7s)-adamantan-1-ylamino)methyl)phenyl)-2-(naphthalen-1-ylmethoxy)quinoline (32k)

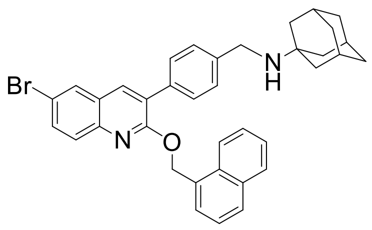

The procedure used to synthesize **32a** was repeated using **31** and (4-(((3s, 5s, 7s)-adamantan-1-ylamino)methyl)phenyl)boronic acid to afford compound **32k** as a yellow solid in 55.1% yield. Mp 72−73 ºC. ^{1}H NMR (400 MHz, Acetone-d_6) δ 8.25 (d, *J* = 8.1 Hz, 1 H), 8.18 (s, 1 H), 8.12 (d, *J* = 1.6 Hz, 1 H), 7.95 (d, *J* = 8.0 Hz, 1 H), 7.90 (d, *J* = 8.4 Hz, 1 H), 7.86 (d, *J* = 8.9 Hz, 1 H), 7.78 (dd, *J* = 8.8, 1.8 Hz, 1 H), 7.74 (d, *J* = 6.8 Hz, 1 H), 7.61 (t, *J* = 7.6 Hz, 1 H), 7.58−7.51 (m, 3 H), 7.48 (t, *J* = 7.6 Hz, 1 H), 7.34 (d, *J* = 7.9 Hz, 2 H), 6.08 (s, 2 H), 3.74 (s, 2 H), 2.77 (br s, 1 H), 1.92−1.45 (m, 15 H). ^{13}C NMR (150 MHz, Acetone-d_6) δ 160.33, 145.20, 143.69, 138.07, 134.90, 134.79, 133.70, 133.24, 132.76, 130.60, 129.97, 129.70, 129.69, 129.47, 128.64, 128.50, 128.26, 128.06, 127.20, 126.78, 126.21, 124.98, 117.90, 67.07, 51.12, 45.09, 43.65, 37.56, 30.59. HRMS (ESI-TOF, m/z): calcd for $C_{37}H_{36}{}^{81}BrN_2O$ [M+H]$^+$ 605.1985; found: 605.1.992

6-Bromo-3-(4-((dimethylamino)methyl)-3-fluorophenyl)-2-(naphthalen-1-ylmethoxy)quinoline (32l)

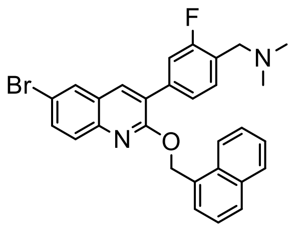

The procedure used to synthesize **32b** was repeated using **31** and (4-((dimethylamino) methyl)-3-fluorophenyl)boronic acid pinacol ester to afford compound **32l** as a white solid in 71.4% yield. Mp 106−107 ºC. ^{1}H NMR (400 MHz, Acetone-*d*$_6$) *δ* 8.30−8.21 (m, 2 H), 8.14 (s, 1 H), 7.96 (d, *J* = 7.8 Hz, 1 H), 7.91 (d, *J* = 8.3 Hz, 1 H), 7.88 (d, *J* = 9.2 Hz, 1 H), 7.82 (d, *J* = 8.9 Hz, 1 H), 7.75 (d, *J* = 6.9 Hz, 1 H), 7.60 (t, *J* = 6.9 Hz, 1 H), 7.55 (t, *J* = 6.9 Hz, 1 H), 7.51−7.44 (m, 2 H), 7.43−7.34 (m, 2 H), 6.10 (s, 2 H), 3.44 (s, 2 H), 2.19 (s, 6 H). ^{13}C NMR (150 MHz, Acetone-*d*$_6$) *δ* 162.47, 160.85, 159.97, 145.43, 138.56, 137.69, 137.63, 134.80, 133.65, 133.56, 132.76, 131.95, 131.92, 130.77, 129.79, 129.72, 129.48, 128.33, 127.88, 127.26, 126.97, 126.96, 126.79, 126.53, 126.43, 126.18, 125.88, 125.86, 124.94, 118.05, 117.01, 116.85, 67.20, 56.63, 56.62, 45.45. HRMS (ESI-TOF, m/z): calcd for $C_{29}H_{25}BrFN_2{}^{81}O$ [M+H]$^+$ 517.1108; found: 517.1117.

6-Bromo-3-(4-((dimethylamino)methyl)-2-fluorophenyl)-2-(naphthalen-1-ylmethoxy)quinoline (32m)

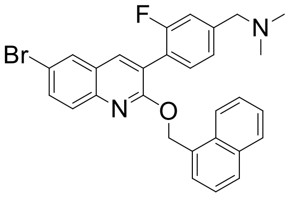

The procedure used to synthesize **32b** was repeated using **31** and (4-((dimethylamino)methyl)-2-fluorophenyl)boronic acid pinacol ester to afford compound **32m** as a white solid in 73.6% yield. Mp 99−100 ºC. ^{1}H NMR (400 MHz, Acetone-*d*$_6$) *δ* 8.21 (s, 1 H), 8.18−8.12 (m, 2 H), 7.92 (d, *J* = 7.2 Hz, 1 H), 7.90−7.85 (m, 2 H), 7.85−7.78 (m, 1 H), 7.66 (d, *J* = 6.9 Hz, 1 H), 7.59−7.49 (m, 2 H), 7.43 (t, *J* = 7.9 Hz, 2 H), 7.14 (d, *J* = 8.9 Hz, 2 H), 6.05 (s, 2 H), 3.42 (s, 2 H), 2.19 (s, 6 H). ^{13}C NMR (150 MHz, Acetone-*d*$_6$) *δ* 161.73, 160.43, 160.10, 145.73, 143.78, 143.73, 139.59, 139.58, 134.69, 133.75, 133.59, 132.60, 132.42, 132.40, 130.70, 129.84, 129.57, 129.35, 127.63, 127.52, 127.06, 126.71, 126.12, 125.07, 125.05, 124.87, 123.22, 123.12, 123.08, 118.03, 116.26, 116.11, 67.11, 63.83, 63.82, 45.56. HRMS (ESI-TOF, m/z): calcd for $C_{29}H_{25}{}^{81}BrFN_2O$ [M+H]$^+$ 517.1108; found: 517.1113.

6-Bromo-3-(4-((dimethylamino)methyl)-3-methoxyphenyl)-2-(naphthalen-1-ylmethoxy)quinoline (32n)

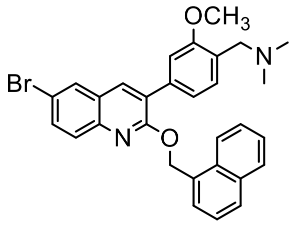

The procedure used to synthesize **32b** was repeated using **31** and (4-((dimethylamino)methyl)-3-methoxyphenyl)boronic acid pinacol ester to afford compound **32n** as a white solid in 78.5% yield. Mp 106−107 ºC. ^{1}H NMR (600 MHz, Acetone-d_6) δ 8.26 (s, 1 H), 8.23 (d, J = 8.3 Hz, 1 H), 8.14 (d, J = 2.2 Hz, 1 H), 7.97 (d, J = 7.5 Hz, 1 H), 7.93 (d, J = 8.3 Hz, 1 H), 7.88 (d, J = 8.8 Hz, 1 H), 7.80 (dd, J = 8.8, 2.3 Hz, 1 H), 7.75 (d, J = 6.8 Hz, 1 H), 7.62−7.57 (m, 1 H), 7.57−7.53 (m, 1 H), 7.49 (dd, J = 8.2, 7.0 Hz, 1 H), 7.34 (d, J = 7.6 Hz, 1 H), 7.22−7.15 (m, 2 H), 6.06 (s, 2 H), 3.35 (s, 2 H), 3.27 (s, 3 H), 2.16 (s, 6 H). ^{13}C NMR (150 MHz, Acetone-d_6) δ 160.26, 157.97, 145.24, 138.11, 136.43, 134.82, 133.66, 133.33, 132.91, 130.67, 130.46, 129.90, 129.69, 129.53, 128.70, 128.21, 128.07, 128.04, 127.44, 126.80, 126.27, 124.92, 121.98, 117.92, 112.64, 67.25, 57.55, 55.24, 45.74. HRMS (ESI-TOF, m/z): calcd for $C_{30}H_{28}BrN_2O_2$ [M+H]$^+$ 527.1329; found: 527.1323.

6-Bromo-3-(4-((dimethylamino)methyl)-2-methoxyphenyl)-2-(naphthalen-1-ylmethoxy)quinoline (32o)

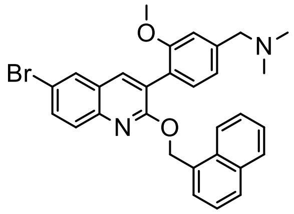

The procedure used to synthesize **32b** was repeated using **31** and (4-((dimethylamino)methyl)-2-methoxyphenyl)boronic acid pinacol ester to afford compound **32o** as a yellow solid in 71.8% yield. Mp 65−66 ºC. ^{1}H NMR (600 MHz, Acetone-d_6) δ 8.12 (dd, J = 8.0, 1.0 Hz, 1 H), 8.08 (d, J = 2.2 Hz, 1 H), 8.04 (s, 1 H), 7.91 (dd, J = 7.5, 1.9 Hz, 1 H), 7.85 (d, J = 6.1 Hz, 1 H), 7.84 (d, J = 6.7 Hz, 1 H), 7.77 (dd, J = 8.9, 2.3 Hz, 1 H), 7.63 (dd, J = 6.9, 0.6 Hz, 1 H), 7.57−7.49 (m, 2 H), 7.42 (dd, J = 8.2, 7.1 Hz, 1 H), 7.23 (d, J = 7.5 Hz, 1 H), 6.98 (s, 1 H), 6.92−6.86 (m, 1 H), 6.01 (s, 2H), 3.56 (s, 3 H), 3.39 (s, 2 H), 2.19 (s, 6 H). ^{13}C NMR (150 MHz, Acetone-d_6) δ 161.08, 158.17, 145.43, 142.42, 138.88, 134.66, 133.89, 133.09, 132.58, 131.46, 130.44, 129.72, 129.36, 129.33, 127.76, 127.42, 127.06,

126.62, 126.50, 126.12, 124.88, 124.83, 121.30, 117.64, 111.83, 66.56, 64.71, 55.65, 45.62. HRMS (ESI-TOF, m/z): calcd for $C_{30}H_{28}BrN_2O_2$ $[M+H]^+$ 527.1329; found: 527.1325.

6-Bromo-3-(6-(4-methylpiperazin-1-yl)pyridin-3-yl)-2-(naphthalen-1-ylmethoxy)quinoline (32p)

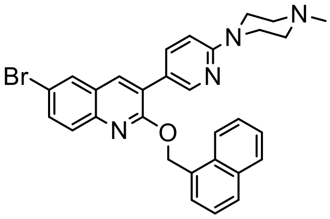

The procedure used to synthesize **32b** was repeated using **31** and (6-(4-methylpiperazin-1-yl)pyridin-3-yl)boronic acid pinacol ester to afford compound **32p** as a white solid in 81.2% yield. Mp 130−131 ºC. ^{1}H NMR (400 MHz, Acetone-d_6) δ 8.42 (d, J = 1.8 Hz, 1 H), 8.23 (d, J = 8.2 Hz, 1 H), 8.18 (s, 1 H), 8.10 (d, J = 1.8 Hz, 1 H), 7.96 (d, J = 7.8 Hz, 1 H), 7.91 (d, J = 8.3 Hz, 1 H), 7.87−7.79 (m, 2 H), 7.79−7.70 (m, 2 H), 7.64−7.52 (m, 2 H), 7.49 (t, J = 7.6 Hz, 1 H), 6.70 (d, J = 8.9 Hz, 1 H), 6.09 (s, 2 H), 3.53 (t, J = 5.0 Hz, 4 H), 2.39 (t, J = 5.0 Hz, 4 H), 2.23 (s, 3 H). ^{13}C NMR (150 MHz, Acetone-d_6) δ 160.38, 159.64, 149.00, 144.96, 139.05, 136.75, 134.79, 133.67, 133.01, 132.78, 130.44, 129.76, 129.66, 129.50, 128.32, 128.18, 127.28, 126.79, 126.24, 125.84, 124.85, 121.44, 117.92, 106.54, 67.06, 55.58, 46.43, 45.55. HRMS (ESI-TOF, m/z): calcd for $C_{30}H_{28}{}^{81}BrN_4O$ $[M+H]^+$ 541.1421; found: 541.1427.

6-Bromo-3-(4-((1H-imidazol-1-yl)methyl)phenyl)-2-(naphthalen-1-ylmethoxy)quinoline (32q)

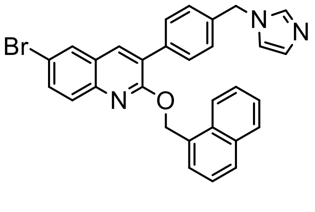

The procedure used to synthesize **32a** was repeated using **31** and (4-((1H-imidazol-1-yl)methyl)phenyl)boronic acid to afford compound **32q** as a yellow oil in 41.1% yield. Mp 177−178 ºC. ^{1}H NMR (600 MHz, Acetone-d_6) δ 8.25−8.18 (m, 2 H), 8.12 (d, J = 2.1 Hz, 1 H), 7.99−7.93 (m, 1 H), 7.91 (d, J = 8.3 Hz, 1 H), 7.86 (d, J = 8.8 Hz, 1 H), 7.80 (dd, J = 8.8, 2.2 Hz, 1 H), 7.72 (d, J = 6.9 Hz, 1 H), 7.68−7.60 (m, 3 H), 7.60−7.52 (m, 2 H), 7.47 (t, J = 7.8 Hz, 1 H), 7.22 (d, J = 8.0 Hz, 2 H), 7.09 (s, 1 H), 6.94 (s, 1 H), 6.07 (s, 2 H), 5.25 (s, 2 H).

^{13}C NMR (150 MHz, Acetone-d_6) δ 160.15, 145.34, 138.59, 138.39, 138.32, 136.48, 134.78, 133.61, 133.46, 132.75, 130.66, 130.09, 129.75, 129.71, 129.46, 128.30, 128.01, 127.95, 127.89, 127.19, 126.79, 126.20, 124.95, 120.09, 117.97, 67.12, 50.44. HRMS (ESI-TOF, m/z): calcd for $C_{30}H_{23}{}^{81}BrN_3O$ $[M+H]^+$ 522.0999; found: 522.0997.

6-Bromo-3-(4-(morpholinomethyl)phenyl)-2-(naphthalen-1-ylmethoxy)quinoline (32r)

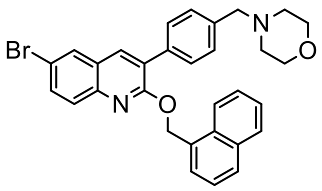

The procedure used to synthesize **32a** was repeated using **31** and (4-(morpholinomethyl)phenyl)boronic acid to afford compound **32r** as a white solid in 86.8% yield. Mp 55−56 ºC. ^1H NMR (600 MHz, Acetone-d_6) δ 8.22 (d, J = 8.4 Hz, 1 H), 8.17 (s, 1 H), 8.09 (d, J = 2.3 Hz, 1 H), 7.94 (dd, J = 8.3, 0.9 Hz, 1 H), 7.88 (d, J = 8.3 Hz, 1 H), 7.85 (d, J = 8.9 Hz, 1 H), 7.78 (dd, J = 8.8, 2.3 Hz, 1 H), 7.71 (d, J = 6.5 Hz, 1 H), 7.61−7.56 (m, 3 H), 7.56−7.52 (m, 1 H), 7.45 (dd, J = 8.2, 7.0 Hz, 1 H), 7.30 (d, J = 8.3 Hz, 2 H), 6.06 (s, 2 H), 3.59 (t, J = 4.6 Hz, 4 H), 3.45 (s, 2 H), 2.36 (br.s, 4 H). ^{13}C NMR (150 MHz, Acetone-d_6) δ 160.22, 145.23, 139.17, 138.12, 135.61, 134.76, 133.65, 133.30, 132.70, 130.60, 130.14, 129.67, 129.53, 129.44, 128.27, 128.14, 127.99, 127.13, 126.74, 126.16, 124.98, 117.92, 67.45, 67.05, 63.45, 54.46. HRMS (ESI-TOF, m/z): calcd for $C_{31}H_{28}BrN_2O_2$ $[M+H]^+$ 539.1329; found: 539.1323.

6-Bromo-2-(naphthalen-1-ylmethoxy)- 3-(1-(tetrahydro-2H-pyran-2-yl)-1H-pyrazol-4-yl) quinoline (32s)

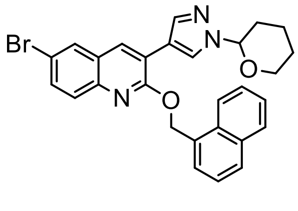

A mixture of 6-bromo-3-iodo-2-(naphthalen-1-ylmethoxy)quinoline **31** (100mg, 0.20 mmol), 1, 1'-Bis(diphenylphosphino)ferrocene-palladium(II)dichloride dichloromethane complex (8 mg, 0.01 mmol), Na_2CO_3 (43 mg, 0.41 mmol) and 1-(2-tetrahydropyranyl)-1H-pyrazole-4-boronic acid pinacol ester (39 mg, 0.22 mmol) in the mixture of toluene (4 mL) and water (2mL) was stirred at 80 °C for 10 h. Water (10 mL) was added and the mixture was extracted

with dichloromethane (10 mL×3), dried with Na_2SO_4 and concentrated. The residue was purified by flash column chromatography with petroleum ether/ethyl acetate (4:1) to afford **32s** as a white solid (82 mg, 79.9%). Mp 150−151 ºC. ^{1}H NMR (400 MHz, Acetone-d_6) δ 8.50 (s, 1 H), 8.34 (d, J = 7.9 Hz, 1 H), 8.10−8.04 (m, 2 H), 8.03−7.97 (m, 3 H), 7.85−7.79 (m, 2 H), 7.74 (d, J = 8.8 Hz, 1 H), 7.65−7.60 (m, 2 H), 7.55 (t, J = 7.6 Hz, 1 H), 6.16−6.10 (m, 2 H), 5.22 (d, J = 9.1 Hz, 1 H), 3.82 (d, J = 11.5 Hz, 1 H), 3.54 (td, J = 11.3, 2.5 Hz, 1 H), 1.84−1.73 (m, 2 H), 1.60−1.48 (m, 4 H). ^{13}C NMR (100 MHz, Acetone-d_6) δ 159.51, 144.25, 138.61, 134.99, 133.62, 133.47, 132.98, 132.65, 130.14, 130.08, 129.66, 129.56, 128.91, 128.90, 128.06, 127.54, 126.85, 126.34, 125.11, 119.80, 117.93, 117.09, 88.23, 67.78, 67.52, 30.99, 25.68, 22.85. HRMS(ESI-TOF, m/z): calcd for $C_{28}H_{25}BrN_3O_2$ [M+H]$^+$ 514.1125; found: 514.1169.

6-Bromo-2-(naphthalen-1-ylmethoxy)- 3-(4-(2-(dimethylamino)-2-oxoethyl)phenyl) quinoline (32t)

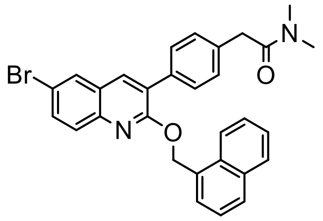

The procedure used to synthesize **32s** was repeated using **31** and N, N-dimethyl-2-(4-(4, 4, 5, 5-tetramethyl-1, 3, 2-dioxaborolan-2-yl)phenyl)acetamide to afford compound **32t** as a white solid in 74.3% yield. Mp 135−136 ºC. ^{1}H NMR (400 MHz, DMSO-d_6) δ 8.28 (s, 1 H), 8.20 (s, 1 H), 8.17 (d, J = 8.1 Hz, 1 H), 7.96 (d, J = 7.6 Hz, 1 H), 7.91 (d, J = 8.3 Hz, 1 H), 7.84−7.78 (m, 2 H), 7.68 (d, J = 6.9 Hz, 1 H), 7.63−7.55 (m, 2 H), 7.53 (d, J = 8.0 Hz, 2 H), 7.47 (t, J = 7.6 Hz, 1 H), 7.17 (d, J = 8.0 Hz, 2 H), 6.01 (s, 2 H), 3.66 (s, 2 H), 2.96 (s, 3 H), 2.80 (s, 3 H). ^{13}C NMR (150 MHz, DMSO-d_6) δ 170.02, 158.90, 143.71, 137.54, 135.95, 133.52, 133.29, 132.56, 132.38, 131.26, 129.78, 129.10, 128.95, 128.77, 128.63, 128.51, 127.31, 126.84, 126.63, 126.42, 126.02, 125.38, 124.04, 116.86, 66.03, 37.15, 35.01. HRMS (ESI-TOF, m/z): calcd for $C_{30}H_{26}{}^{81}BrN_2O_2$ [M+H]$^+$ 527.1152; found: 527.1153.

6-Bromo-2-(naphthalen-1-ylmethoxy)-3-(4-(pyrrolidin-1-yl)phenyl)quinoline (32u)

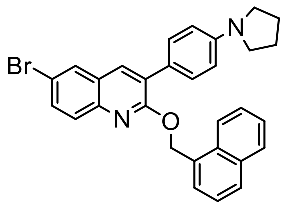

The procedure used to synthesize **32s** was repeated using **31** and 4-(1-pyrrolidinyl)benzeneboronic acid pinacol ester to afford compound **32u** as a white solid in 85.5% yield. Mp 131−132 °C. ^{1}H NMR (400 MHz, Acetone-d_6) δ 8.27 (d, J = 8.4 Hz, 1 H), 8.19−8.02 (m, 2 H), 7.96 (d, J = 7.6 Hz, 1 H), 7.91 (d, J = 8.3 Hz, 1 H), 7.82 (d, J = 8.7 Hz, 1 H), 7.77 (d, J = 6.9 Hz, 1 H), 7.73 (d, J = 8.8 Hz, 1 H), 7.62 (t, J = 7.3 Hz, 1 H), 7.60−7.39 (m, 4 H), 6.51 (d, J = 8.7 Hz, 2 H), 6.09 (s, 2 H), 3.25 (t, J = 6.4 Hz, 4 H), 2.06−1.94 (m, 4 H). ^{13}C NMR (150 MHz, Acetone-d_6) δ 160.57, 148.74, 144.53, 136.20, 134.79, 133.91, 132.76, 132.47, 131.13, 130.25, 129.60, 129.58, 129.47, 129.01, 128.43, 128.09, 127.19, 126.76, 126.24, 124.97, 123.19, 117.72, 112.09, 66.86, 48.12, 26.03. HRMS (ESI-TOF, m/z): calcd for $C_{30}H_{26}{}^{81}BrN_2O$ [M+H]$^+$ 511.1203; found: 511.1210.

6-Bromo-3-(2-(dimethylamino)quinazolin-6-yl)-2-(naphthalen-1-ylmethoxy)quinoline (32v)

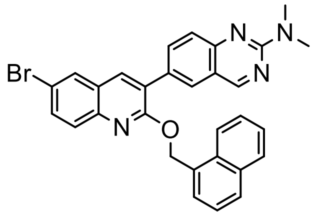

The procedure used to synthesize **32s** was repeated using **31** and (2-(dimethylamino)quinazolin-6-yl)boronic acid pinacol ester to afford compound **32v** as a yellow solid in 73.6% yield. Mp 165−166 °C. ^{1}H NMR (600 MHz, Acetone-d_6) δ 8.87 (s, 1 H), 8.37−8.31 (m, 2 H), 8.17 (d, J = 2.2 Hz, 1 H), 8.06 (d, J = 2.0 Hz, 1 H), 8.03−7.95 (m, 2 H), 7.95−7.86 (m, 2 H), 7.82 (dd, J = 8.9, 2.2 Hz, 1 H), 7.77 (d, J = 6.9 Hz, 1 H), 7.70−7.63 (m, 1 H), 7.62−7.57 (m, 1 H), 7.48 (dd, J = 8.1, 7.1 Hz, 1 H), 7.45 (d, J = 8.8 Hz, 1 H), 6.12 (s, 2 H), 3.27 (s, 6 H). ^{13}C NMR (150 MHz, Acetone-d_6) δ 162.36, 161.01, 160.26, 152.92, 145.29, 138.02, 136.10, 134.84, 133.62, 133.41, 132.82, 130.66, 130.28, 129.83, 129.75, 129.55, 129.31, 128.45, 128.11, 127.60, 127.29, 126.83, 126.25, 125.90, 125.13, 119.70, 118.03, 67.26, 37.23. HRMS (ESI-TOF, m/z): calcd for $C_{30}H_{24}{}^{81}BrN_4O$ [M+H]$^+$ 537.1108; found: 537.1099.

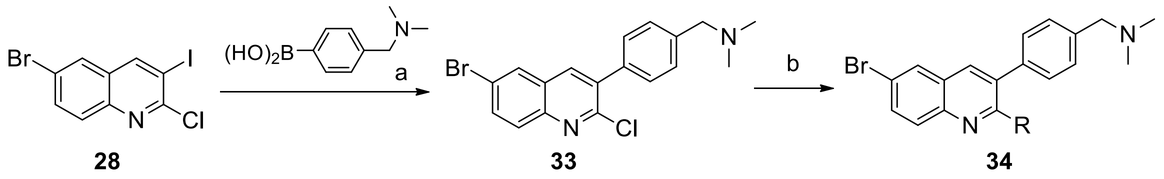

Scheme 7. Synthesis of compound 34. Reagents and conditions: (a) (4-((dimethylamino)methyl)phenyl)boronic acid, $Pd(PPh_3)_4$, Na_2CO_3, toluene/H_2O, 14 h; (b) NaH , THF, RT, 6 h. (v) MeI, acetone, 4 h, RT.

6-Bromo-2-chloro-3-(4-((dimethylamino)methyl)phenyl)quinoline(33a)

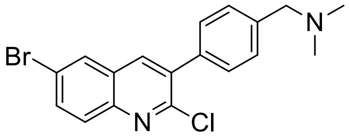

To a solution of 6-bromo-2-chloro-3-iodoquinoline **28a** (400 mg, 1.09 mmol) in the mixture of 4 mL toluene and 3mL H_2O Pd $(PPh_3)_4$ (57 mg, 0.05 mmol), Na_2CO_3 (73 mg, 0.69 mmol) and (4-((dimethylamino)methyl)phenyl)boronic acid (254 mg, 1.42 mmol) was added. After stirring at 80 °C for 10 h, water (10 mL) was added and the mixture was extracted with dichloromethane (10 mL×3), dried with Na_2SO_4 and concentrated. The residue was purified by flash column chromatography with petroleum ether/ethyl acetate/triethylamine (40:15:1) to give a white solid (321 mg, 85.8%). ^{1}H NMR (400 MHz, Acetone-d_6) δ 8.35 (s, 1 H), 8.29 (s, 1 H), 7.94 (s, 2 H), 7.56 (d, J = 7.9 Hz, 2 H), 7.49 (d, J = 7.9 Hz, 2 H), 3.50 (s, 2 H), 2.24 (s, 6 H).. HRMS (ESI, m/z)[M+H]$^+$: 375.0248.

2-Chloro-3-(4-((dimethylamino) methyl)phenyl)quinoline (33b)

The procedure used to synthesize **33a** was repeated using **28c** and (4-((dimethylamino)methyl)phenyl)boronic acid to afford compound **33b** as a white solid in 81.5% yield. ^{1}H NMR (400 MHz, Acetone-d_6) δ 8.35 (s, 1 H), 8.05 (d, J = 8.1 Hz, 1 H), 8.00 (d, J = 8.5 Hz, 1 H), 7.86–7.82 (m, 1 H), 7.67 (dd, J = 11.1, 4.0 Hz, 1 H), 7.46 (d, J = 7.9 Hz, 2 H), 7.43 (d, J = 7.9 Hz, 2 H), 3.50 (s, 2 H), 2.22 (s, 6 H). HRMS (ESI, m/z)[M+H]$^+$: 297.1178.

3-(4-((Dimethylamino)methyl)phenyl)-2-(naphthalen-1-ylmethoxy)quinoline (34a)

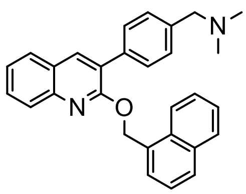

To a solution of **33b** (100 mg, 0.34 mmol) and 1-Naphthalenemethanol (68 mg, 0.43 mmol) in 4 mL DMF was added Cs_2CO_3 (140 mg, 0.43 mmol). After stirring at 80 °C for 8 h, water (20 mL) was added and the mixture was extracted with dichloromethane (10 mL×3), dried with Na_2SO_4 and concentrated. The residue was purified by flash column chromatography with petroleum ether/ethyl acetate/triethylamine (40:15:1) to afford **34a** as a white solid (128.9 mg, 91.2%). Mp 93−95 ºC. ^{1}H NMR (600 MHz, Acetone-d_6) δ 8.25 (d, J = 8.4 Hz, 1 H), 8.21 (s, 1 H), 7.98−7.89 (m, 3 H), 7.88 (d, J = 8.3 Hz, 1 H), 7.74 (d, J = 6.9 Hz, 1 H), 7.69 (t, J = 7.6 Hz, 1 H), 7.65−7.58 (m, 3 H), 7.55 (t, J = 7.4 Hz, 1 H), 7.46 (t, J = 7.6 Hz, 2 H), 7.29 (d, J = 7.9 Hz, 2 H), 6.09 (s, 2 H), 3.39 (s, 2 H), 2.17 (s, 6 H). ^{13}C NMR (150 MHz, Acetone-d_6) δ 159.80, 146.56, 139.94, 139.07, 136.01, 134.76, 133.94, 132.73, 130.31, 130.09, 129.56, 129.43, 129.32, 128.62, 128.04, 127.65, 127.22, 127.11, 126.72, 126.70, 126.18, 125.30, 124.99, 66.77, 64.35, 45.55. HRMS (ESI-TOF, m/z): calcd for $C_{29}H_{27}N_2O$ $[M+H]^+$ 419.2118; found: 419.2110.

6-Bromo-3-(4-((dimethylamino)methyl)phenyl)-2-phenoxyquinoline (34b)

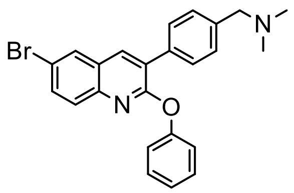

The procedure used to synthesize **34a** was repeated using **33a** and phenol to afford compound **34b** as a white solid in 85.8% yield. Mp 75−76 ºC. ^{1}H NMR (400 MHz, Acetone-d_6) δ 8.35 (s, 1 H), 8.18 (s, 1 H), 7.78 (d, J = 8.0 Hz, 2 H), 7.73 (dd, J = 8.9, 2.1 Hz, 1 H), 7.55 (d, J = 8.9 Hz, 1 H), 7.50−7.38 (m, 4 H), 7.33−7.21 (m, 3 H), 3.48 (s, 2 H), 2.22 (s, 6 H). ^{13}C NMR (150 MHz, Acetone-d_6) δ 160.43, 154.80, 144.95, 140.66, 139.03, 135.47, 133.48, 130.62, 130.26, 130.17, 129.85, 129.64, 128.59, 128.48, 125.64, 122.84, 118.61, 64.43, 45.63. HRMS (ESI-TOF, m/z): calcd for $C_{24}H_{22}BrN_2O$ $[M+H]^+$ 433.0910; found: 433.0905.

6-Bromo-3-(4-((dimethylamino)methyl)phenyl)-2-benzyloxyquinoline (34c)

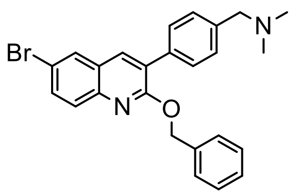

The procedure used to synthesize **34a** was repeated using **33a** and benzyl alcohol to afford compound **34c** as a white solid in 84.2% yield. Mp 66−67 ºC. ^{1}H NMR (400 MHz, Acetone-*d*$_6$) *δ* 8.21 (s, 1 H), 8.12 (s, 1 H), 7.80 (d, *J* = 8.9 Hz, 1 H), 7.77 (d, *J* = 9.9 Hz, 1 H), 7.68 (d, *J* = 7.8 Hz, 2 H), 7.52 (d, *J* = 7.6 Hz, 2 H), 7.41 (d, *J* = 7.9 Hz, 2 H), 7.36 (t, *J* = 7.5 Hz, 2 H), 7.29 (t, *J* = 7.0 Hz, 1 H), 5.62 (s, 2 H), 3.46 (s, 2 H), 2.22 (s, 6 H). ^{13}C NMR (150 MHz, Acetone-*d*$_6$) *δ* 160.29, 145.23, 140.40, 138.30, 138.10, 135.61, 133.28, 130.62, 130.14, 129.63, 129.47, 129.20, 128.77, 128.53, 128.24, 128.01, 117.88, 68.63, 64.41, 45.59. HRMS (ESI-TOF, m/z): calcd for $C_{25}H_{24}BrN_2O$ $[M+H]^+$ 447.1067; found: 447.1079.

6-Bromo-3-(4-((dimethylamino)methyl)phenyl)-2-phenethoxyquinoline (34d)

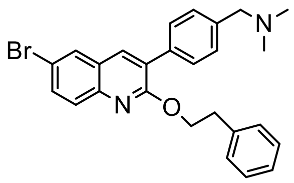

The procedure used to synthesize **34a** was repeated using **33a** and phenethyl alcohol to afford compound **34d** as a white solid in 88.4% yield. Mp 55−56 ºC. ^{1}H NMR (400 MHz, Acetone-*d*$_6$) *δ* 8.15 (s, 1 H), 8.09 (s, 1 H), 7.81−7.69 (m, 2 H), 7.52 (d, *J* = 8.1 Hz, 2 H), 7.40 (d, *J* = 7.9 Hz, 2 H), 7.35−7.25 (m, 4 H), 7.25−7.20 (m, 1 H), 4.72 (t, *J* = 6.7 Hz, 2 H), 3.49 (s, 2 H), 3.12 (t, *J* = 6.6 Hz, 2 H), 2.25 (s, 6 H). ^{13}C NMR (150 MHz, Acetone-*d*$_6$) *δ* 160.47, 145.33, 139.82, 137.81, 135.60, 133.15, 130.56, 130.20, 130.04, 129.62, 129.47, 129.16, 128.15, 127.87, 127.12, 117.70, 68.08, 64.40, 45.56, 35.84. HRMS (ESI-TOF, m/z): calcd for $C_{26}H_{26}BrN_2O$ $[M+H]^+$ 461.1223; found: 461.1239.

6-Bromo-3-(4-((dimethylamino)methyl)phenyl)-2-((2-methoxybenzyl)oxy) quinoline (34e)

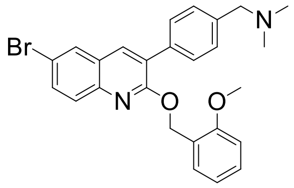

The procedure used to synthesize **34a** was repeated using **33a** and 2-methoxybenzyl alcohol to afford compound **34e** as a yellow oil in 81.5% yield. Mp 96−97 ºC. ^{1}H NMR (600 MHz, Acetone-d_6) δ 8.20 (s, 1 H), 8.11 (d, J = 2.2 Hz, 1 H), 7.81 (d, J = 8.8 Hz, 1 H), 7.76 (dd, J = 8.9, 2.2 Hz, 1 H), 7.73−7.64 (m, 2 H), 7.44 (dd, J = 7.5, 1.4 Hz, 1 H), 7.40 (d, J = 8.2 Hz, 2 H), 7.29 (td, J = 8.2, 1.7 Hz, 1 H), 7.03 (d, J = 8.1 Hz, 1 H), 6.90 (td, J = 7.4, 0.7 Hz, 1 H), 5.60 (s, 2 H), 3.91 (s, 3 H), 3.44 (s, 2 H), 2.21 (s, 6 H). ^{13}C NMR (150 MHz, Acetone-d_6) δ 160.48, 158.37, 145.31, 140.42, 137.92, 135.64, 133.20, 130.59, 130.14, 129.93, 129.87, 129.64, 129.41, 128.28, 127.97, 126.10, 121.04, 117.77, 111.32, 64.49, 64.44, 55.81, 45.61. HRMS (ESI-TOF, m/z): calcd for $C_{26}H_{26}BrN_2O_2$ [M+H]$^+$ 477.1172; found: 477.1166.

6-Bromo-3-(4-((dimethylamino)methyl)phenyl)-2-((3-methoxybenzyl)oxy) quinoline (34f)

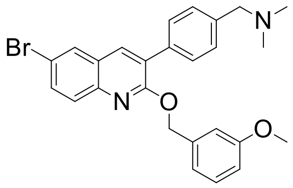

The procedure used to synthesize **34a** was repeated using **33a** and 3-methoxybenzyl alcohol to afford compound **34f** as a colorless oil in 92.4% yield. Mp 52−53 ºC. ^{1}H NMR (600 MHz, Acetone-d_6) δ 8.20 (s, 1 H), 8.11 (d, J = 2.1 Hz, 1 H), 7.80 (d, J = 8.8 Hz, 1 H), 7.76 (dd, J = 8.9, 2.2 Hz, 1 H), 7.69 (d, J = 8.2 Hz, 2 H), 7.42 (d, J = 8.2 Hz, 2 H), 7.26 (t, J = 7.9 Hz, 1 H), 7.09 (d, J = 1.7 Hz, 1 H), 7.06 (d, J = 7.5 Hz, 1 H), 6.84 (dd, J = 8.1, 2.4 Hz, 1 H), 5.58 (s, 2 H), 3.76 (s, 3 H), 3.45 (s, 2 H), 2.22 (s, 6 H). ^{13}C NMR (150 MHz, Acetone-d_6) δ 160.75, 160.22, 145.22, 140.45, 139.79, 138.06, 135.60, 133.26, 130.60, 130.20, 130.14, 129.61, 129.46, 128.24, 127.98, 120.55, 117.88, 114.23, 113.68, 68.46, 64.42, 55.45, 45.61. HRMS (ESI-TOF, m/z): calcd for $C_{26}H_{26}BrN_2O_2$ [M+H]$^+$ 477.1172; found: 477.1167.

6-Bromo-3-(4-((dimethylamino)methyl)phenyl)-2-((4-methoxybenzyl)oxy)quinoline (34g)

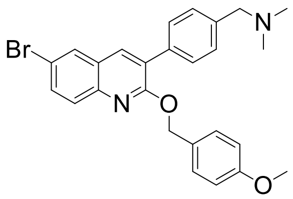

The procedure used to synthesize **34a** was repeated using **33a** and 4-methoxybenzyl alcohol to afford compound **34g** as a yellow solid in 82.4% yield. Mp 46−47 ºC. ^{1}H NMR (600 MHz,

Acetone-d_6) δ 8.19 (s, 1 H), 8.11 (d, J = 2.2 Hz, 1 H), 7.81 (d, J = 8.8 Hz, 1 H), 7.77 (dd, J = 8.9, 2.2 Hz, 1 H), 7.66 (d, J = 8.2 Hz, 2 H), 7.47 (d, J = 8.7 Hz, 2 H), 7.40 (d, J = 8.1 Hz, 2 H), 7.27 (d, J = 8.7 Hz, 1 H), 6.91 (d, J = 8.7 Hz, 2 H), 6.87 (d, J = 8.7 Hz, 1 H), 5.54 (s, 2 H), 3.78 (s, 3 H), 3.45 (s, 2 H), 2.21 (s, 6 H). ^{13}C NMR (150 MHz, Acetone-d_6) δ 160.39, 160.35, 145.24, 140.35, 138.00, 135.64, 133.21, 130.68, 130.58, 130.09, 129.60, 129.43, 128.22, 127.94, 117.77, 114.54, 114.32, 68.46, 64.40, 55.48, 45.60. HRMS (ESI-TOF, m/z): calcd for $C_{26}H_{26}BrN_2O_2$ [M+H]$^+$ 477.1172; found: 477.1171.

6-Bromo-3-(4-((dimethylamino)methyl)phenyl)-2-((3-fluorobenzyl)oxy)quinoline (34h)

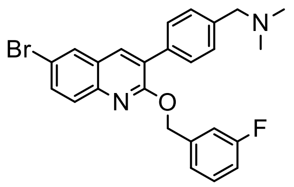

The procedure used to synthesize **34a** was repeated using **33a** and 3-fluorobenzyl alcohol to afford compound **34h** as a yellow solid in 85.4% yield. Mp 82−83 ºC. ^{1}H NMR (600 MHz, Acetone-d_6) δ 8.21 (s, 1 H), 8.12 (d, J = 2.1 Hz, 1 H), 7.79 (d, J = 8.8 Hz, 1 H), 7.76 (dd, J = 8.9, 2.1 Hz, 1 H), 7.71−7.65 (m, 2 H), 7.43 (d, J = 8.3 Hz, 2 H), 7.39 (td, J = 7.9, 5.9 Hz, 1 H), 7.33 (d, J = 7.7 Hz, 1 H), 7.30−7.24 (m, 1 H), 7.09−7.01 (m, 1 H), 5.62 (s, 2 H), 3.46 (s, 2 H), 2.22 (s, 6 H). ^{13}C NMR (150 MHz, Acetone-d_6) δ 164.48, 162.86, 160.04, 145.14, 141.25, 141.20, 140.52, 138.17, 135.50, 133.33, 131.10, 131.04, 130.63, 130.13, 129.62, 129.48, 128.21, 128.04, 124.33, 124.31, 118.00, 115.27, 115.19, 115.12, 115.05, 67.79, 67.78, 64.42, 45.60. . HRMS (ESI-TOF, m/z): calcd for $C_{25}H_{23}BrFN_2O$ [M+H]$^+$ 465.0972; found: 465.0974.

6-Bromo-2-((4-chlorobenzyl)oxy) -3-(4-((dimethylamino)methyl)phenyl)quinoline (34i)

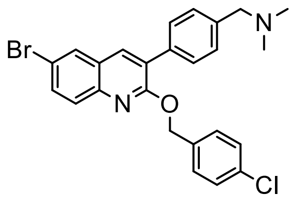

The procedure used to synthesize **34a** was repeated using **33a** and 4-chlorobenzyl alcohol to afford compound **34i** as a yellow solid in 82.4% yield. Mp 120−121 ºC. ^{1}H NMR (600 MHz, Acetone-d_6) δ 8.21 (s, 1 H), 8.12 (d, J = 2.0 Hz, 1 H), 7.79 (d, J = 8.8 Hz, 1 H), 7.76 (dd, J = 8.9, 2.1 Hz, 1 H), 7.70−7.64 (m, 2 H), 7.56−7.52 (m, 2 H), 7.42 (d, J = 8.2 Hz, 2 H),

7.40−7.35 (m, 2 H), 5.59 (s, 2 H), 3.45 (s, 2 H), 2.22 (s, 6 H). ^{13}C NMR (150 MHz, Acetone-d_6) δ 160.08, 145.14, 140.53, 138.16, 137.29, 135.50, 133.83, 133.32, 130.63, 130.55, 130.11, 129.61, 129.48, 129.25, 128.19, 128.03, 117.97, 67.83, 64.43, 45.63. HRMS (ESI-TOF, m/z): calcd for $C_{25}H_{23}{}^{81}BrClN_2O$ [M+H]$^+$ 483.0656; found: 483.0639.

6-Bromo-2-((2, 6-difluorobenzyl)oxy)-3-(4-((dimethylamino)methyl)phenyl)quinoline (34j)

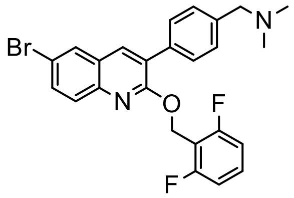

The procedure used to synthesize **34a** was repeated using **33a** and 2, 6-difluorobenzyl alcohol to afford compound **34j** as a yellow solid in 91.2% yield. Mp 81−82 ºC. ^{1}H NMR (600 MHz, Acetone-d_6) δ 8.22 (s, 1 H), 8.13 (d, J = 2.1 Hz, 1 H), 7.82 (d, J = 8.8 Hz, 1 H), 7.79 (dd, J = 8.9, 2.2 Hz, 1 H), 7.63 (d, J = 8.2 Hz, 2 H), 7.51−7.44 (m, 1 H), 7.35 (d, J = 8.1 Hz, 2 H), 7.12−7.02 (m, 2 H), 5.68 (s, 2 H), 3.41 (s, 2 H), 2.19 (s, 6 H). ^{13}C NMR (150 MHz, Acetone-d_6) δ 163.72, 163.66, 162.06, 162.01, 159.82, 145.02, 140.48, 138.25, 135.33, 133.35, 132.13, 132.06, 131.99, 130.63, 129.93, 129.63, 129.40, 128.09, 127.89, 118.05, 113.91, 113.78, 113.66, 112.40, 112.37, 112.26, 112.23, 64.37, 56.86, 56.83, 56.81, 45.61. HRMS (ESI-TOF, m/z): calcd for $C_{25}H_{22}BrF_2N_2O$ [M+H]$^+$ 483.0878; found: 483.0887.

2-([1, 1'-Biphenyl]-4-ylmethoxy)-6-bromo-3-(4-((dimethylamino)methyl)phenyl)-quinoline (34k)

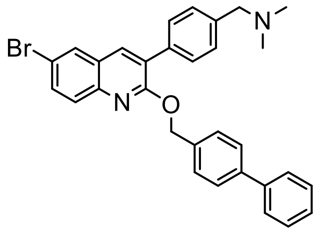

The procedure used to synthesize **34a** was repeated using **33a** and [1, 1'-biphenyl]-4-ylmethanol to afford compound **34k** as a yellow solid in 89.8% yield. Mp 96−97 ºC. ^{1}H NMR (600 MHz, Acetone-d_6) δ 8.22 (s, 1 H), 8.13 (d, J = 2.2 Hz, 1 H), 7.82 (d, J = 8.9 Hz, 1 H),

7.77 (dd, *J* = 8.9, 2.2 Hz, 1 H), 7.70 (d, *J* = 8.2 Hz, 2 H), 7.68−7.63 (m, 4 H), 7.61 (d, *J* = 8.3 Hz, 2 H), 7.48−7.43 (t, *J* = 7.8 Hz, 2 H), 7.42 (d, *J* = 8.2 Hz, 2 H), 7.35 (t, *J* = 7.4 Hz, 1 H), 5.66 (s, 2 H), 3.45 (s, 2 H), 2.21 (s, 6 H).^{13}C NMR (150 MHz, Acetone-d_6) δ 160.27, 145.22, 141.46, 141.26, 140.48, 138.11, 137.44, 135.59, 133.28, 130.62, 130.14, 129.70, 129.63, 129.47, 129.38, 128.25, 128.21, 128.02, 127.71, 127.69, 117.89, 68.36, 64.44, 45.62. HRMS (ESI-TOF, m/z): calcd for $C_{31}H_{28}BrN_2O$ [M+H]$^+$ 523.1380; found: 523.1389.

2-(4-(Benzyloxy)phenoxy)-6-bromo-3-(4-((dimethylamino)methyl)phenyl)quinoline (34l)

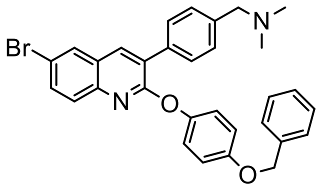

The procedure used to synthesize **34a** was repeated using **33a** and 4-(benzyloxy)phenol to afford compound **34l** as a yellow solid in 79.9% yield. Mp 103−104 ºC. ^{1}H NMR (600 MHz, Acetone-d_6) δ 8.31 (s, 1 H), 8.15 (s, 1 H), 7.77 (d, *J* = 7.8 Hz, 2 H), 7.71 (dd, *J* = 8.9, 1.3 Hz, 1 H), 7.55 (d, *J* = 8.9 Hz, 1 H), 7.51 (d, *J* = 7.6 Hz, 2 H), 7.46 (d, *J* = 7.9 Hz, 2 H), 7.41 (t, *J* = 7.5 Hz, 2 H), 7.34 (t, *J* = 7.3 Hz, 1 H), 7.20 (d, *J* = 8.8 Hz, 2 H), 7.08 (d, *J* = 8.8 Hz, 2 H), 5.14 (s, 2 H), 3.47 (s, 2 H), 2.22 (s, 6 H). ^{13}C NMR (150 MHz, Acetone-d_6) δ 160.75, 156.90, 148.20, 144.98, 140.61, 138.87, 138.41, 135.53, 133.41, 130.58, 130.17, 129.79, 129.61, 129.29, 128.65, 128.49, 128.36, 123.79, 118.44, 116.20, 70.85, 64.43, 45.64. HRMS (ESI-TOF, m/z): calcd for $C_{31}H_{28}BrN_2O_2$ [M+H]$^+$ 539.1329; found: 539.1334.

6-Bromo-3-(4-((dimethylamino)methyl)phenyl)-2-(pyridin-4-ylmethoxy)quinoline (34m)

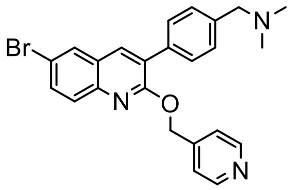

The procedure used to synthesize **34a** was repeated using **33a** and 4-pyridylcarbinol to afford compound **34m** as a white solid in 84.7% yield. Mp 86−87 ºC. ^{1}H NMR (600 MHz, Acetone-d_6) δ 8.54 (dd, *J* = 4.4, 1.5 Hz, 2 H), 8.24 (s, 1 H), 8.13 (s, 1 H), 7.76 (d, *J* = 1.3 Hz, 2 H), 7.71 (d, *J* = 8.2 Hz, 2 H), 7.47 (d, *J* = 8.0 Hz, 2 H), 7.44 (d, *J* = 5.9 Hz, 2 H), 5.65 (s, 2 H), 3.49 (s, 2 H), 2.24 (s, 6 H). ^{13}C NMR (150 MHz, Acetone-d_6) δ 159.83, 150.65, 147.16, 145.07, 140.45, 138.30, 135.47, 133.38, 130.65, 130.16, 129.62, 129.58, 128.17, 128.09,

122.65, 118.11, 66.97, 64.37, 45.55. HRMS (ESI-TOF, m/z): calcd for $C_{24}H_{23}BrN_3O$ $[M+H]^+$ 448.1019; found: 448.1016.

2-(Benzylthio)-6-bromo-3-(4-((dimethylamino)methyl)phenyl) quinoline (34n)

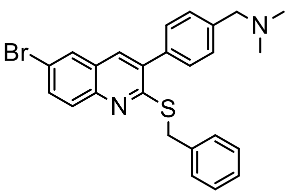

The procedure used to synthesize **34a** was repeated using **33a** and benzyl mercaptan to afford compound **34n** as a yellow solid in 71.4% yield. Mp 67−68 ºC. ^{1}H NMR (600 MHz, Acetone-d_6) δ 8.13 (s, 1 H), 7.98 (d, J = 8.9 Hz, 1 H), 7.96 (s, 1 H), 7.83 (d, J = 8.9 Hz, 1 H), 7.49 (d, J = 7.8 Hz, 2 H), 7.47 (d, J = 8.0 Hz, 2 H), 7.43 (d, J = 7.9 Hz, 2 H), 7.28 (t, J = 7.5 Hz, 2 H), 7.20 (t, J = 7.3 Hz, 1 H), 4.56 (s, 2 H), 3.46 (s, 2 H), 2.21 (s, 6 H). ^{13}C NMR (150 MHz, Acetone-d_6) δ 160.10, 146.51, 141.09, 139.18, 136.75, 136.21, 135.16, 133.62, 130.81, 130.39, 130.21, 130.04, 129.67, 129.19, 128.33, 127.81, 119.27, 64.41, 45.64, 35.22. HRMS (ESI-TOF, m/z): calcd for $C_{25}H_{24}BrN_2S$ $[M+H]^+$ 463.0844; found: 463.0844.

6-Bromo-3-(4-((dimethylamino)methyl)phenyl)- 2-(1H-imidazol-1-yl)quinoline (34o)

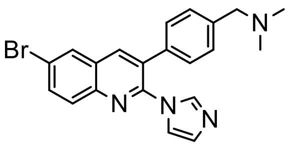

The procedure used to synthesize **34a** was repeated using **33a** and imidazole to afford compound **34o** as a yellow solid in 34.3% yield. Mp 155−156 ºC. ^{1}H NMR (600 MHz, Acetone-d_6) δ 8.48 (s, 1 H), 8.32 (d, J = 2.2 Hz, 1 H), 8.00 (d, J = 9.0 Hz, 1 H), 7.95 (dd, J = 9.0, 2.2 Hz, 1 H), 7.72−7.68 (m, 1 H), 7.41 (d, J = 8.2 Hz, 2 H), 7.33 (d, J = 8.2 Hz, 2 H), 7.22 (t, J = 1.3 Hz, 1 H), 6.95−6.91 (m, 1 H), 3.47 (s, 2 H), 2.22 (s, 6 H). ^{13}C NMR (150 MHz, Acetone-d_6) δ 148.26, 145.66, 141.04, 140.39, 137.91, 136.35, 134.64, 131.37, 131.24, 130.82, 130.21, 129.73, 129.60, 129.51, 121.24, 120.05, 64.30, 45.58. HRMS (ESI-TOF, m/z): calcd for $C_{21}H_{20}BrN_4$ $[M+H]^+$ 407.0866; found: 407.0855.

6-Bromo-3-(4-((dimethylamino)methyl)phenyl)-2-methoxyquinoline (34p)

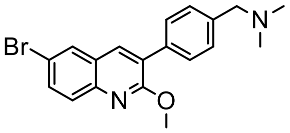

A mixture of **33a** (80 mg, 0.21 mmol), NaH (11 mg, 0.32mmol) and MeI (0.03 mL, 0.5 mmol) in tetrahydrofuran (4 mL) was stirred at 40°C for 4 h. Then water (10 mL) was added to quench the reaction and the mixture was extracted with dichloromethane (10 mL×3), dried with Na_2SO_4 and concentrated. The residue was purified by flash column chromatography with petroleum ether/ethyl acetate/triethylamine (40:15:1) to afford **34p** as a yellow oil (55 mg, 82.6%). Mp 125−126 ºC. ^{1}H NMR (400 MHz, Acetone-d_6) δ 8.16 (s, 1 H), 8.10 (s, 1 H), 7.80−7.77 (m, 2 H), 7.63 (d, J = 7.9 Hz, 2 H), 7.42 (d, J = 7.9 Hz, 2 H), 4.06 (s, 3 H), 3.46 (s, 2 H), 2.22 (s, 6 H). ^{13}C NMR (150 MHz, Acetone-d_6) δ 160.99, 145.37, 137.88, 135.74, 133.21, 130.61, 130.09, 129.65, 129.51, 129.47, 128.25, 127.95, 117.74, 64.44, 54.08, 45.61. HRMS (ESI-TOF, m/z): calcd for $C_{19}H_{20}BrN_2O$ $[M+H]^+$ 371.0751; found: 371.0750.

References:

1. Chandrasekhar, S.; Babu, G. S. K.; Mohapatra, D. K., Practical Syntheses of (2S)-R207910 and (2R)-R207910. *Eur. J. Org. Chem.* **2011,** *11*, 2057-2061.

2. Lu, Y.; Zheng, M.; Wang, B.; Fu, L.; Zhao, W.; Li, P.; Xu, J.; Zhu, H.; Jin, H.; Yin, D.; Huang, H.; Upton, A. M.; Ma, Z., Clofazimine analogs with efficacy against experimental tuberculosis and reduced potential for accumulation. *Antimicrob Agents Chemother* **2011,** *55*, 5185-5193.
